# Phosphine and Selenoether *peri*-Substituted Acenaphthenes and Their Transition-Metal Complexes: Structural and NMR Investigations

**DOI:** 10.1021/acs.inorgchem.3c02255

**Published:** 2023-09-18

**Authors:** Lutao Zhang, Francesca A. Christie, Anna E. Tarcza, Helena G. Lancaster, Laurence J. Taylor, Michael Bühl, Olga L. Malkina, J. Derek Woollins, Cameron L. Carpenter-Warren, David B. Cordes, Alexandra M. Z. Slawin, Brian A. Chalmers, Petr Kilian

**Affiliations:** † EaStChem School of Chemistry, University of St. Andrews, St. Andrews KY16 9ST, Fife, U.K.; ‡ Institute of Wolfberry Science, Ningxia Academy of Agriculture and Forestry Sciences, Yinchuan 750002, China; § Institute of Inorganic Chemistry, Slovak Academy of Sciences, Bratislava 84 536, Slovakia; ∥ Department of Chemistry, Khalifa University, Abu Dhabi 127788, United Arab Emirates

## Abstract

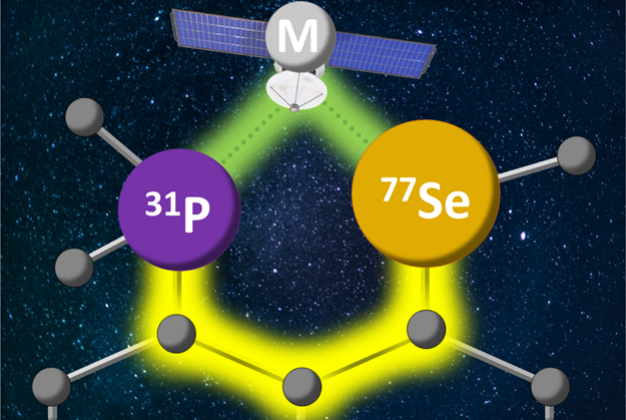

A series of *peri*-substituted acenaphthene-based phosphine selenoether bidentate ligands Acenap(*i*Pr_2_P)(SeAr) (**L1**–**L4**, Acenap = acenaphthene-5,6-diyl, Ar = Ph, mesityl, 2,4,6-trisopropylphenyl and supermesityl) were prepared. The rigid acenaphthene framework induces a forced overlap of the phosphine and selenoether lone pairs, resulting in a large magnitude of through-space ^4^*J*_PSe_ coupling, ranging from 452 to 545 Hz. These rigid ligands **L1**–**L4** were used to prepare a series of selected late d-block metals, mercury, and borane complexes, which were characterized, including by multinuclear NMR and single-crystal X-ray diffraction. The Lewis acidic motifs (BH_3_, Mo(CO)_4_, Ag^+^, PdCl_2_, PtCl_2_, and HgCl_2_) bridge the two donor atoms (P and Se) in all but one case in the solid-state structures. Where the bridging motif contained NMR-active nuclei (^11^B, ^107^Ag, ^109^Ag, ^195^Pt, and ^199^Hg), *J*_PM_ and *J*_SeM_ couplings are observed directly, in addition to the altered *J*_PSe_ in the respective NMR spectra. The solution NMR data are correlated with single-crystal diffraction data, and in the case of mercury(II) complexes, they are also correlated with the solid-state NMR data and coupling deformation density calculations. The latter indicate that the through-space interaction dominates in free **L1**, while in the **L1HgCl**_**2**_ complex, the main coupling pathway is via the metal atom and not through the carbon framework of the acenaphthene ring system.

## Abstract 2

A series of hybrid acenaphthene-based ligands with phosphine and selenoether groups in peri-positions were synthesized alongside with their selected late transition-metal complexes. Multinuclear NMR and single-crystal X-ray diffraction were used to correlate the coordination modes in the solid and solution state with a view to evaluate their (potential) hemilabile nature.

## Introduction

**Figure 1:**
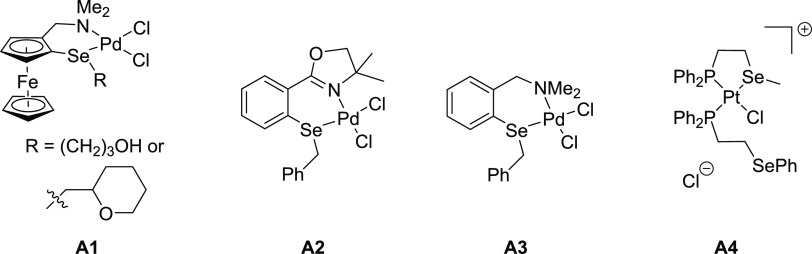
Examples of the hybrid selenoether complexes.

In contrast to anionic chalcogenolates RE^–^, neutral chalcogenoethers RER′ have been traditionally seen as weak donors, showing a reasonable affinity to bind to soft d-block metal centers only,^1^ although a small number of complexes with p-block metals and metalloids have also been reported.^2^ The weakly bonding nature of chalcogenoethers has been utilized in the construction of hybrid hemilabile ligands in which the soft sulfur, selenium, or tellurium donor atom can bind weakly, and reversibly, to the metal centers. Concordantly, another stronger donor atom, such as phosphorus or nitrogen, anchors the metal fragment to the hybrid ligand.^1^ Hemilabile ligands have been used in catalysis,^3−5^ supramolecular chemistry,^6^ and sensing applications.^7^ A large number of hybrid ligand types with selenoether functionality have been developed (see Figure 1). These include N,Se 1,2-ferrocenyl ligands **A1**,^8^ N,Se *ortho*-substituted benzyl backbone ligands **A2**, **A3**,^9^ and P,Se ethylene bridge ligands, such as **A4**,^10^ as some key examples from the literature.

**Figure 2:**
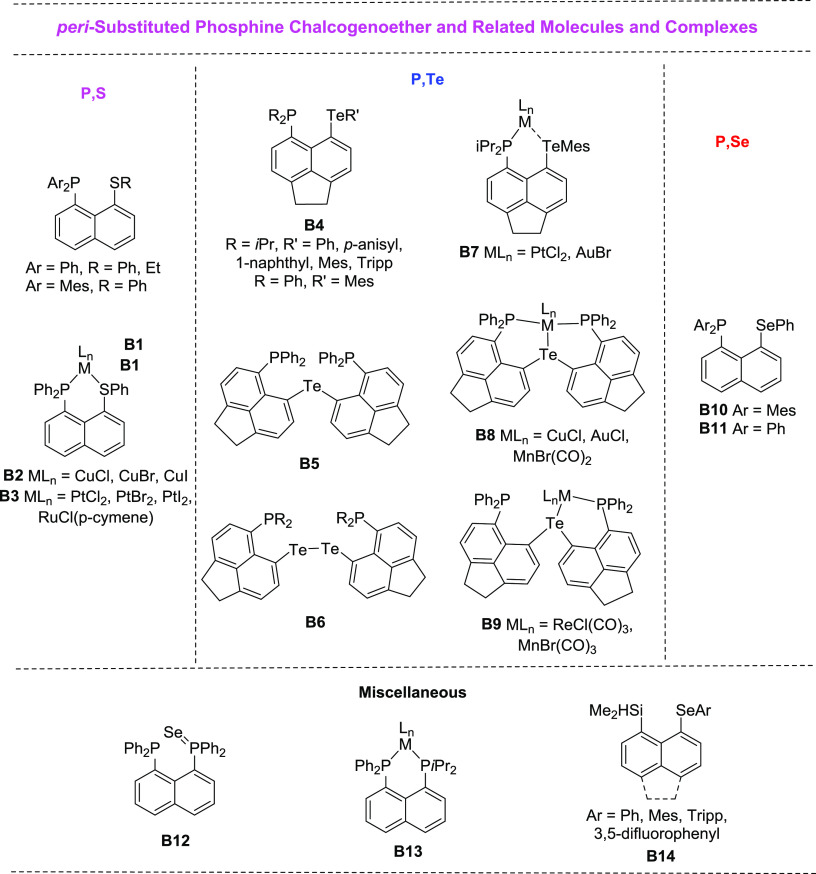
Selected phosphine chalcogenoether *peri*-substituted molecules, their complexes, and related molecules reported in the literature (Mes = 2,4,6-trimethylphenyl; Tripp = 2,4,6-triisopropylphenyl).

In addition to the motifs above, rigid *peri*-substituted naphthalene or acenaphthene backbones have been used as suitable scaffolds for hybrid ligands, with the two donor atoms in the *peri*-positions preorganized to act as a chelating ligand forming a six-membered C_3_PME metallacycle upon coordination to the metal fragment. A few series of *peri*-substituted naphthalene-based molecules with potential hybrid hemilabile ligand characteristics have been reported in the literature alongside their respective metal complexes in some cases. These include phosphine chalcogenoethers with P,S, P,Se, and P,Te *peri*-atom combinations, as shown in Figure 2.

Phosphine-thioether ligands, such as **B1** (Figure 2), were made through the stepwise reactions of lithiated intermediates with respective alkyl or aryl disulfides and chlorophosphines.^11−13^ Only one of these molecules, Nap(PPh_2_)(SPh), has been utilized as a ligand. The Cu(I) complexes (**B2**) formed Cu–(μX)_2_–Cu bridged dimers (X = halogen),^11^ while the Pt(II) and Ru(I) complexes **B3** were mononuclear.^14^

The *peri*-substituted systems with phosphine and telluroether groups have been studied more extensively and include simple hybrid ligands **B4**(15) (Figure 2) as well as geminally dinaphthyl substituted species **B5**(16) and ditelluride **B6**.^17^ One of the **B4** molecules, Acenap(P*i*Pr_2_)(TeMes), was used as a bidentate ligand toward Pt(II) and Au(I) fragments in complexes **B7**.^15^ The dinaphthyl ligand **B5** acted as bidentate or tridentate ligand in mononuclear complexes, κ^2^P,P′,κTe (**B8**) and κP,κTe (**B9**).^18^

Most relevantly to this paper, only a single structural report on phosphine selenoether *peri*-substituted species has been found in the CSD. Compound **B10**, Nap(PMes_2_)(SePh) (Figure 2), has been synthesized with a view of stabilizing a two-center three-electron bonding motif upon single electron oxidation of **B10**.^13^ The arsenic analogue of **B10** (with the PMes_2_ group replaced by an AsMes_2_ group) has also been investigated.^13^ Unfortunately, no ^77^Se NMR parameters have been reported for **B10** or its arsenic analogue.

The phenyl selenoether **B11** has been synthesized and characterized, including ^77^Se NMR data, and displayed a remarkable ^31^P–^77^Se coupling in solution (^4TS^*J*_PSe_ 391 Hz, note TS superscript indicates through-space coupling), as observed in both the ^31^P (as satellites) and ^77^Se NMR (as a doublet) spectra (δ_P_ −12.9; δ_Se_ 439.6 ppm).^12,19^ In contrast to the P,S and P,Te congeners, no metal complexes of any P,Se *peri*-substituted species with phosphine and selenoether functionalities have been reported.

A recent in-depth study of *J*_PP_ and *J*_PSe_ through-space coupling used a related species, **B12**, as a model compound, and highlighted the recent advances in computational methods with respect to determining the relative contributions from through-space and through-bond coupling pathways.^20^

A comprehensive study on heteroleptic bis(phosphine) metal complexes **B13** showed the changes in the through-space *J*_PP_ on coordination to the metal fragments and correlated these with the *peri*-region geometry as observed by single-crystal diffraction.^21^ Several molecular systems **B14** with *peri*-gap Si–H···Se interactions displayed significant magnitudes of through-space *J*_SeH_ and *J*_SeSi_ couplings;^22^ analysis of the bonding in selected examples by computational methods indicated the presence of a chalcogen–hydride bond.^23^

In this paper, we report syntheses of a series of potential hemilabile P,Se *peri*-substituted ligands as well as a number of their complexes. Possessing a combination of two NMR-active (^31^P (*I* = 1/2, 100%) and ^77^Se (*I* = 1/2, 7.6%)) donor atoms, and in several cases also NMR-active (*I* = 1/2) metals, it has been hoped that useful correlations can be made between the solution and solid-state NMR data, and those from single-crystal diffraction, to provide additional insight into the nature of the (hemilabile) bonding in P,Se hybrid ligands.

## Results and Discussion

### Synthesis

#### Ligands **L1–L4**

**Scheme 1:**
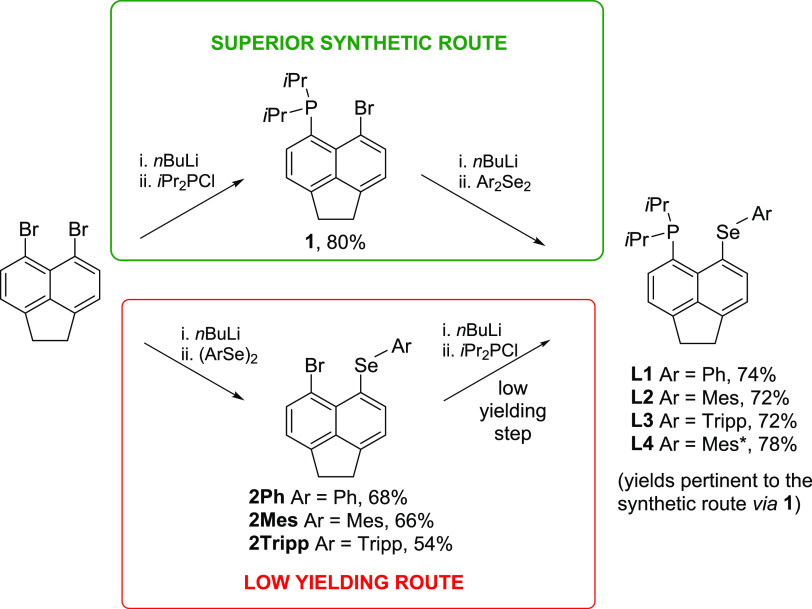
Two Alternative Syntheses of **L1–L4** via Intermediate Compounds **1** and **2** Mes = 2,4,6-trimethylphenyl, Tripp = 2,4,6-tri-i-propylphenyl, and Mes* = 2,4,6-tri-t-butylphenyl.

Phosphino-selanyl acenaphthenes **L1**–**L4** were synthesized via stepwise attachment of phosphine and selenoether functionalities to the 5,6-dibromoacenaphthene starting material. There are two possible synthetic routes to achieve the target Acenap(PR_2_)(SeR) compounds, in which either the phosphino group or the selanyl group is added to the acenaphthene scaffold first, followed by addition of the other group (Scheme 1).

Woollins et al. reported the synthesis and characterization data of 5-bromo-6-(phenylselanyl)acenaphthene (**2Ph**) previously.^24^ We have adopted their method (see Scheme 1) to prepare **2Ph** (68% yield), as well as novel analogues with bulkier aryl groups bound to selenium, **2Mes** (66% yield), and **2Tripp** (54% yield). Compounds **2Ph**, **2Mes**, and **2Tripp** were sufficiently air-stable to allow for purification using column chromatography on silica, and a material of analytical purity was obtained.

In the subsequent step, the diisopropylphosphino group was expected to be attached to **2Ph**, **2Mes**, and **2Tripp**, via lithium-halogen exchange and coupling with *i*Pr_2_PCl, to synthesize the desired acenaphthenes **L1**–**L3** (see Scheme 1). An analogous synthetic path has been used recently by Wang to prepare Acenap(PMes_2_)(SePh) by reacting Acenap(Br)(SePh) with *n*BuLi and subsequently Mes_2_PCl.^13^ However, in our hands, the reactions with *i*Pr_2_PCl gave low yields of the desired products (Scheme 1, bottom). To address this, an alternative synthetic pathway was adopted in which the order of attaching the phosphine and arylselanyl groups was reversed (Scheme 1, top).

In this pathway, the dialkylphosphino moiety was added first to synthesize **1**, Acenap(P*i*Pr_2_)(Br) (80% yield).^25^ The lithium-halogen exchange reaction of **1** to give the intermediate Acenap(P*i*Pr_2_)(Li) was followed by a Se–C coupling reaction with diaryl diselenides affording **L1**–**L4** in yields of 72–78%, making this the preferred synthetic pathway. While the reactions leading to **L1**–**L4** were performed under an inert atmosphere due to the air- and moisture-sensitive nature of the reagents and intermediates, the workup of **L1**–**L4** was performed in air as these compounds showed no signs of decomposition in air at ambient temperature. **L2**–**L4** were purified by column chromatography on silica, whereas **L1** was purified via recrystallization.

**Scheme 2:**
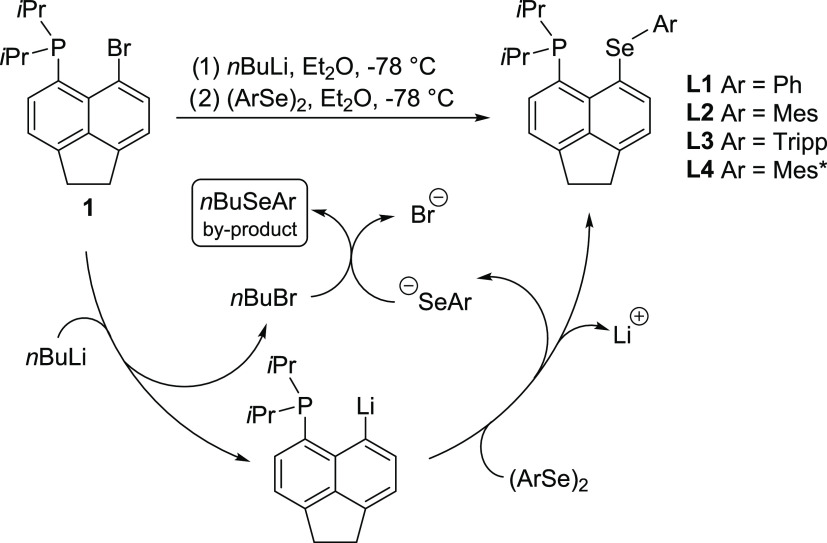
Tentative Mechanism of the Se–C Coupling Reaction with Diaryl Diselenides *n*BuSeAr have been identified as byproducts in this reaction.

The Se–C coupling reactions used to prepare **L1**–**L4** utilize diaryl diselenides and proceed with the formation of aryl(*n*-butyl)selane byproducts (general formula *n*BuSeAr); these have been separated on a chromatography column, and their identity was confirmed by ^1^H, ^13^C{^1^H}, and ^77^Se NMR spectroscopy for Ar = Ph and Mes* and also by single-crystal X-ray crystallography for the latter (see the SI). The tentative mechanism of *n*BuSeAr formation involves the reaction of 1-bromobutane with arylselenoate, as shown in Scheme 2. The aryl(*n*-butyl)selanes are removed efficiently by washing the solid crude product with cold hexane or on the chromatography column.

#### Complexes with **L1–L4** Ligands

Given the presence of the lone pairs on both phosphorus and selenium atoms and their arrangement in the *peri*-region of the acenaphthene backbone, **L1**–**L4** were expected to act as κP,κSe bidentate ligands. However, the selenoether group is a much weaker donor; hence, complexes with **L1**–**L4** acting as a monodentate donor (κP only) were also seen as a viable structural alternative.

**Scheme 3:**
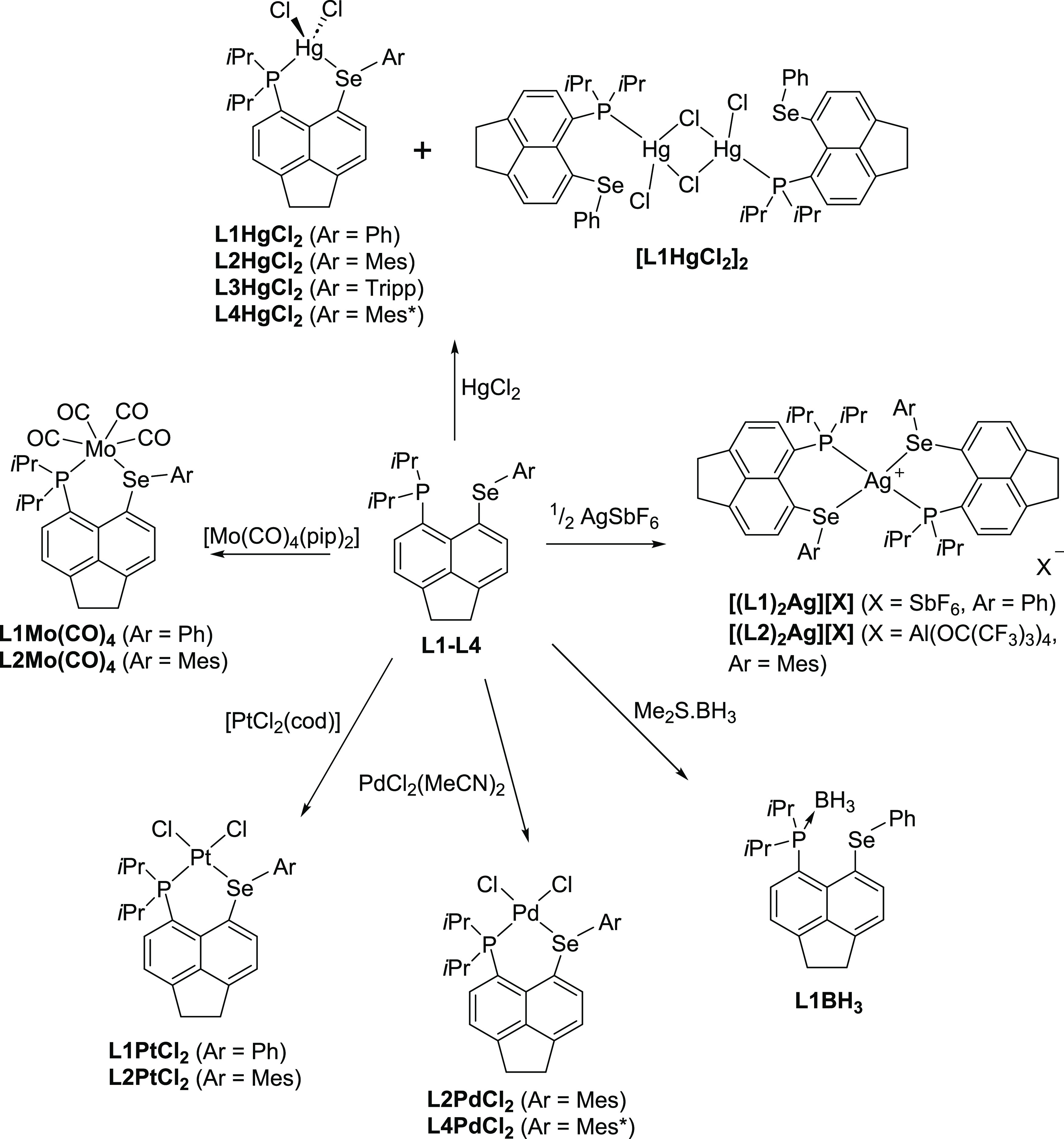
Syntheses of Metal Complexes of Bidentate Phosphine Selenoether Ligands **L1**–**L4** Note: cod = 1,5-cyclooctadiene and pip = piperidine.

We used various transition-metal precursors, such as carbonyls and halides, as starting materials to obtain the coordination complexes shown in Scheme 3. A general complexation reaction procedure involved preparation of a solution or suspension of the ligand (**L1**–**L4**) in dichloromethane (DCM) or ethanol, to which the metal-containing precursor was added as either a solid or a solution at room temperature, and the mixture was stirred overnight. Removal of the volatiles *in vacuo* afforded the desired complexes. Further purification (e.g., column chromatography and hexane wash) was carried out where appropriate. All novel complexes were characterized by multinuclear NMR (^1^H, ^13^C{^1^H} DEPTQ (with the exception of L2PtCl_2_ and L3HgCl_2_), ^31^P{^1^H}, and ^77^Se{^1^H} and where applicable also by ^11^B{^1^H} and ^195^Pt{^1^H}) NMR. All but two complexes (L2PtCl_2_ and L3HgCl_2_) were also characterized by either HRMS (high-resolution mass spectrometry) or elemental microanalysis or both. All complexes prepared in this work were found to be air- and moisture-stable. The silver complexes, **[(L1)**_**2**_**Ag]SbF**_**6**_ and **[(L2)**_**2**_**Ag](Al(OC(CF**_**3**_**)**_**3**_**)**_**4**_**)**, were notably light-sensitive and decomposed as solids and in solution within a matter of hours when exposed to daylight. Attempts to grow diffraction quality crystals of **L4HgCl**_**2**_ gave a small amount of crystals that were shown to have composition of **[L4Hg**_**2**_**Cl**_**4**_**][L4Hg**_**3**_**Cl**_**6**_**]**, i.e., the desired compound with additional weakly coordinated HgCl_2_ (see crystallographic discussion below). Based on the elemental analysis results, the bulk of the material was **L4HgCl**_**2**_, with only a smaller amount of excess HgCl_2_ present.

**Scheme 4:**
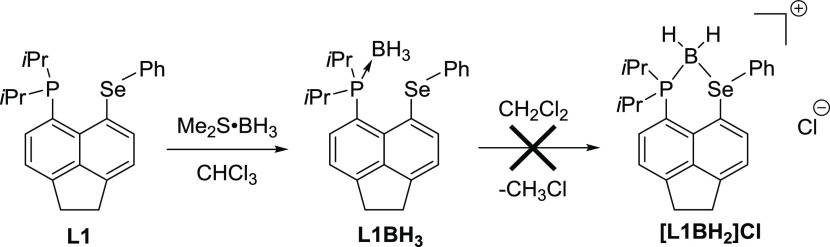
Synthesis of **L1BH**_**3**_ by Complexation of **L1** with Me_2_S·BH_3_ and the Expected Cyclization Reaction

In addition to the metal complexes above, the borane adduct of **L1** was also prepared. The reaction of **L1** with Me_2_S·BH_3_ afforded the phosphine borane **L1BH**_**3**_ in a very good yield of 73% (Scheme 3). We investigated if **L1** would mimic the chemistry of the similar borane adduct Acenap(PPh_2_)(PPh_2_(BH_3_)), which in chlorinated solvents undergoes a cyclization reaction forming boronium salt.^26^ However, the formation of a cyclic boronium salt (such as **[L1BH**_**2**_**]Cl** shown in Scheme 4) was not observed during the reaction of **L1** with excess Me_2_S·BH_3_ in chloroform or dichloromethane, with **L1BH**_**3**_ being the sole product of the reaction.

### NMR Spectroscopy

#### Ligands **L1–L4**

Many of the component elements of species synthesized in this study have NMR-active nonquadrupolar isotopes, and as such NMR provides valuable information with regards to the interactions across the *peri*-gap. The ^31^P{^1^H} NMR spectra of **L1–L4** display singlets within a very narrow range (δ_P_ −6.0 to −6.5 ppm). In all cases, these singlets are equipped with ^77^Se satellites, from which ^4TS^*J*_PSe_ magnitudes ranging from 452.2 to 545.0 Hz were extracted, with the magnitude increasing slightly with the electron donating ability of the aryl group bound to selenium (Table 1). The complementary doublets were observed in the ^77^Se{^1^H} NMR spectra of **L1–L4** with δ_Se_ values ranging from 283.8 to 425.3 ppm. The large magnitudes of ^4TS^*J*_PSe_ in **L1**–**L4** contrast strongly to the ^3^*J*_SeP_ of only 11 Hz observed for the nonrigid phosphine selenoethers RSe(CH_2_)_2_PPh_2_ (R = Me, Ph).^10^ These observations reinforce the notion that a significant interaction between lone pairs of P and Se atoms occurs in **L1–L4**, leading to a large magnitude of the through-space coupling. A detailed study on coupling pathways in related phosphine selenide Nap(PPh_2_(=Se))(PPh_2_), **B12** (see Figure 2), was published recently.^20^ The magnitude of the ^31^P–^77^Se coupling is much smaller in this compound (54.0 Hz) compared to those found in **L1**–**L4**, however this is concomitant with differing geometry and larger P···Se separation observed in the latter (3.41 Å vs 3.06 to 3.14 Å in **L1**–**L4**).^27^

**Table 1:** Selected NMR Data for Free Ligands **L1**–**L4**

	δ_P_ (ppm)	δ_Se_ (ppm)	^4TS^*J*_PSe_ (Hz)
**L1**	–6.5	425.3	452.2
**L2**	–6.5	315.7	466.6
**L3**	–6.4	283.8	476.8
**L4**	–6.0	378.3	545.0

#### Complexes with **L1–L4** Ligands

The multinuclear NMR data of metal complexes reported herein are summarized in Table 2. Upon coordination of **L1** or **L2** to a Mo(CO)_4_ moiety, the phosphorus nuclei in the resulting complexes **L1Mo(CO)**_**4**_ and **L2Mo(CO)**_**4**_ are significantly deshielded (Δδ_P_ 47.3 ppm for **L1Mo(CO)**_**4**_) and the selenium nuclei become more shielded (Δδ_Se_ −31.2 ppm for **L1Mo(CO)**_**4**_) vs the free ligand. There is also a dramatic reduction in the magnitude of *J*_PSe_ from 452.2 to 14.8 Hz in **L1Mo(CO)**_**4**_ and to 37.0 Hz in **L2Mo(CO)**_**4**_ as observed in both the ^31^P{^1^H} and ^77^Se{^1^H} NMR spectra.

**Table 2:** NMR Parameters of the Metal Complexes Reported in This Paper^a^

	δ_P_ (ppm)	δ_Se_ (ppm)	*J*_PSe_ (Hz)	*J*_PM_ (Hz)	*J*_SeM_ (Hz)
**L1PtCl**_**2**_	10.0	332.0	<2	3528.5	656.3
**L2PtCl**_**2**_	10.8	307.4	6.5	3419.2	357.3
**L2PdCl2**	38.1	324.1	<2		
**L4PdCl2**	36.4	411.5	24.5		
**L1Mo(CO)**_**4**_	40.8	394.1	14.7		
**L2Mo(CO)**_**4**_	42.3	284.7	37.0		
**[L1**_**2**_**Ag]SbF**_**6**_^b^	26.2	368.9	195, 14^d^	494.0, 426.2^e^	43, 38^e^
**[L2**_**2**_**Ag][Al(OC(CF**_**3**_**)**_**3**_**)**_**4**_**]**^c^	27.2	256.8	273, 12^d^	506.1, 439.0^e^	55.0, 47.8^e^
**L1HgCl**_**2**_	54.0	378.3	86.7	6610.7	721.3
**[L1HgCl**_**2**_**]**_**2**_ (SS NMR)		351.2	37.5		785.0
**L2HgCl**_**2**_	55.8	279.3	186.9	6264.3	909.3
**L2HgCl**_**2**_ (SS NMR)	56.0	262.8	205.0	6136.6	≈1040
**L3HgCl**_**2**_	55.6	246.6	204.3	6160.1	not observed^f^
**L4HgCl**_**2**_	59.3	325.5	210.8	6276.4	not observed^f^
**L1BH**_**3**_	46.5	412.6	not observed^e^	≈35	not observed^g^

a Both solution and solid-state (CP-MAS SS) NMR data are included.

b ^2^*J*_PP_ 58.2 Hz.

c ^2^*J*_PP_ 73.5 Hz.

d Values for the two P atoms in the complex.

e Values for ^109^Ag and ^107^Ag isotopomers.

f Low signal-to-noise ratio in ^77^Se{^1^H} NMR spectrum precluded observation of ^199^Hg satellites.

g Signal broadening precluded reading of *J* couplings.

An even more dramatic change in the magnitude of *J*_PSe_ takes place on coordination to platinum(II) or palladium(II) centers. In **L1PtCl**_**2**_ and **L2PdCl**_**2**_, no ^77^Se satellites are observed in the ^31^P{^1^H} NMR spectra, indicating the *J*_PSe_ magnitude of less than ca. 2 Hz. This is corroborated by ^77^Se{^1^H} NMR spectra, which show singlets, and thus, there is no observable coupling to ^31^P. Small but detectable ^2^*J*_PSe_ couplings (6.5 and 24.5 Hz) were observed in both ^31^P{^1^H} and ^77^Se{^1^H} spectra of **L2PtCl**_**2**_ and **L4PdCl**_**2**_.

Coordination of platinum(II) or palladium(II) to **L1**, **L2**, or **L4** results in a shift of δ_P_ to high frequency (Δδ_P_ up to 44.6 ppm), while δ_Se_ is shifted to low frequency for the Pt(II) complexes **L1PtCl**_**2**_ and **L2PtCl**_**2**_ (Δδ_Se_ −93.3 ppm for the former) and to higher frequencies for the Pd(II) complexes **L2PdCl**_**2**_ and **L4PdCl**_**2**_ (Δδ_Se_ 33.2 ppm for the latter).

**Figure 3:**
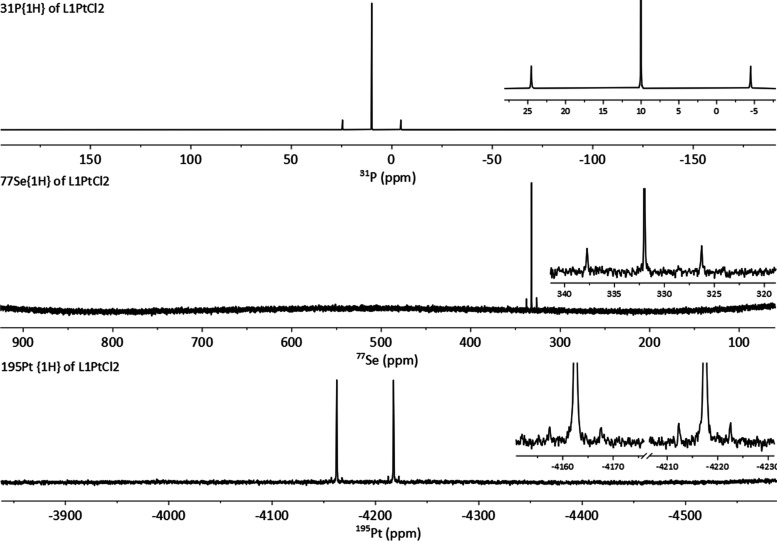
^31^P{^1^H} (top), ^77^Se{^1^H} (center), and ^195^Pt{^1^H} (bottom) NMR spectra (with expansions) of **L1PtCl**_**2**_ recorded at 121.5, 57.3, and 64.2 MHz, respectively, in CDCl_3_.

**Figure 4:**
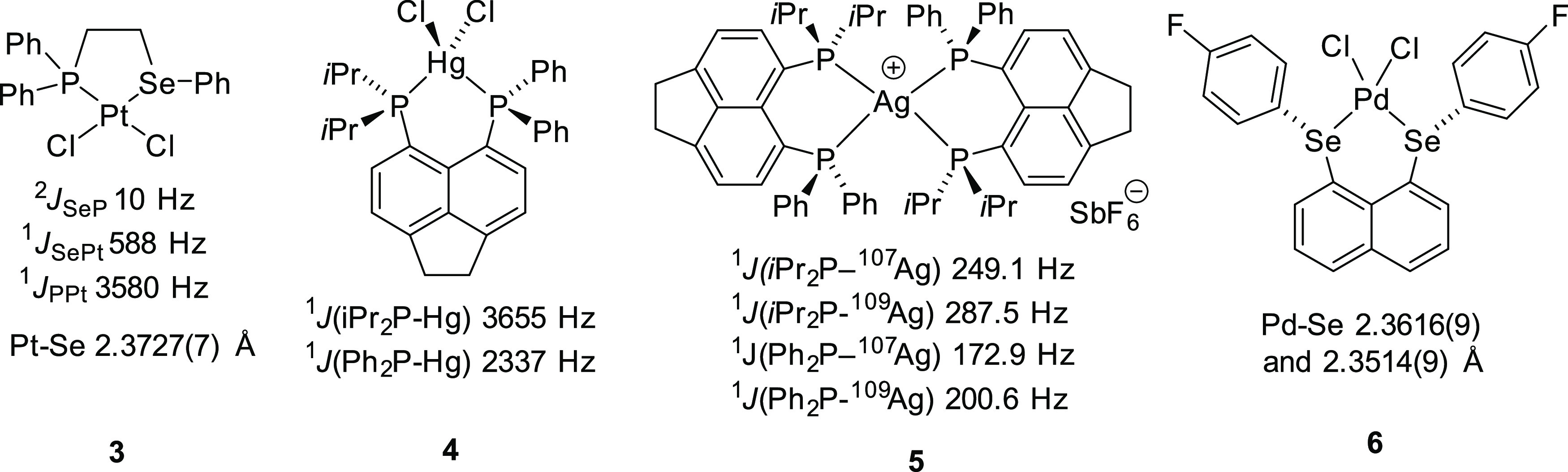
Literature complexes related to the species reported in this paper.

Using **L1PtCl**_**2**_ as an example, both ^31^P{^1^H} and ^77^Se{^1^H} spectra display well-resolved satellite peaks for the ^195^Pt isotopologue (^195^Pt; *I* = 1/2, 34%) with ^1^*J*_PPt_ = 3528.5 Hz and ^1^*J*_SePt_ = 656.6 Hz (Figure 3). Both couplings are complemented in the ^195^Pt{^1^H} NMR spectrum, which displays a doublet with ^77^Se satellites at δ_Pt_ −4190.0 ppm. These are reliably similar to the coupling constants observed for the (structurally verified) Pt chelate complex **3** (^1^*J*_PPt_ = 3580 Hz and ^1^*J*_SePt_ = 588 Hz, see Figure 4).^28^

**Figure 5:**
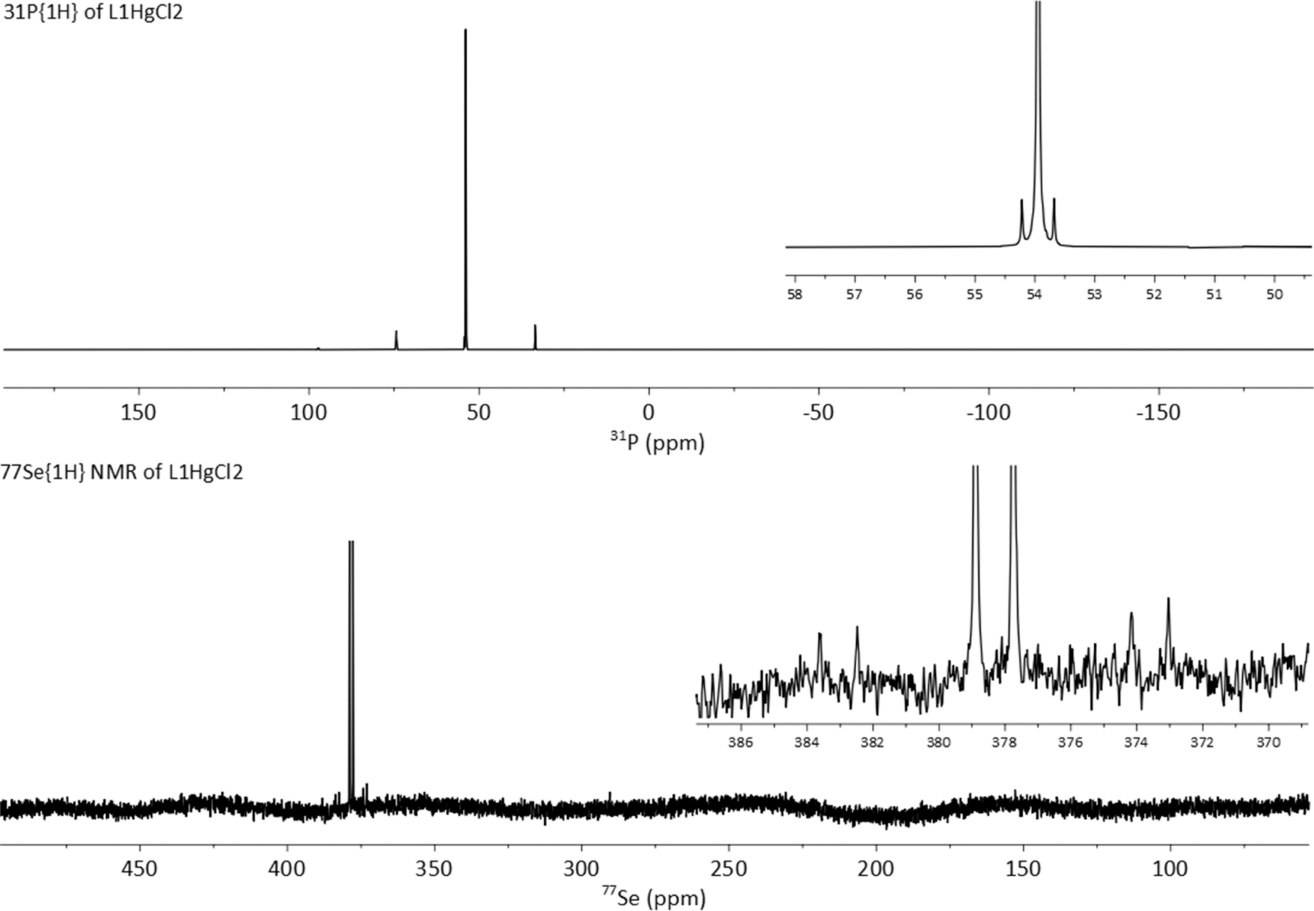
^31^P{^1^H} (top) and ^77^Se{^1^H} (bottom) NMR spectra of **L1HgCl**_**2**_ with expansions recorded at 162.0 and 76.4 MHz, respectively, in CDCl_3_.

Addition of one equivalent of HgCl_2_ to **L1** results in the formation of the complex of composition **L1HgCl**_**2**_. The ^2^*J*_PSe_ coupling was obtained from the solution ^31^P{^1^H} NMR spectrum, which shows ^77^Se satellites of the singlet at δ_P_ 54.0 ppm with *J*_PSe_ of 86.7 Hz and the complementary doublet at δ_Se_ 378.3 ppm in the ^77^Se{^1^H} NMR spectrum (Figure 5). In addition, the ^199^Hg satellites were observed in the ^31^P{^1^H} NMR spectrum showing a remarkably large ^1^*J*_PHg_ of 6,611 Hz (^199^Hg, *I* = 1/2, 16.9%). The ^1^*J*_PHg_ coupling observed in **L1HgCl**_**2**_ vastly exceeds the ^1^*J*_PHg_ magnitudes of 3655 and 2337 Hz observed in the related HgCl_2_ complex **4** (Figure 4).^21^

The ^77^Se{^1^H} NMR spectrum of **L1HgCl**_**2**_ (solution in CDCl_3_) also shows coupling of ^77^Se to ^199^Hg, with well-resolved satellites (*J*_SeHg_ 721.3 Hz) (Figure 5).

Two different structures of the mercury complex with **L1** were observed crystallographically: the monomeric complex **L1HgCl**_**2**_ and a dimeric complex **(L1HgCl**_**2**_**)**_**2**_·CHCl_3_. In the former, the phosphorus atom in **L1** forms a conventional bond to Hg center (2.4082(6) Å, cf. ∑*r*_covalent_ 2.39 Å),^29^ while the Se–Hg bond is rather elongated (2.8084(5) Å, cf. ∑*r*_covalent_ 2.52 Å).^29^ Despite this, it is well within the ∑*r*_vdW_, which is 3.95 Å.^30^

In the latter complex, **(L1HgCl**_**2**_**)**_**2**_·CHCl_3_, the P–Hg contact remains short (P–Hg 2.4305(9) Å); however, the selenium atom is coordinated even more loosely to the mercury center (P···Se 3.2100(4) Å). The Hg···Se distance is thus still within the ∑*r*_vdW_ (3.95 Å)^30^ although the interaction is significantly weakened compared to the other Hg complexes in this work.

**Figure 6:**
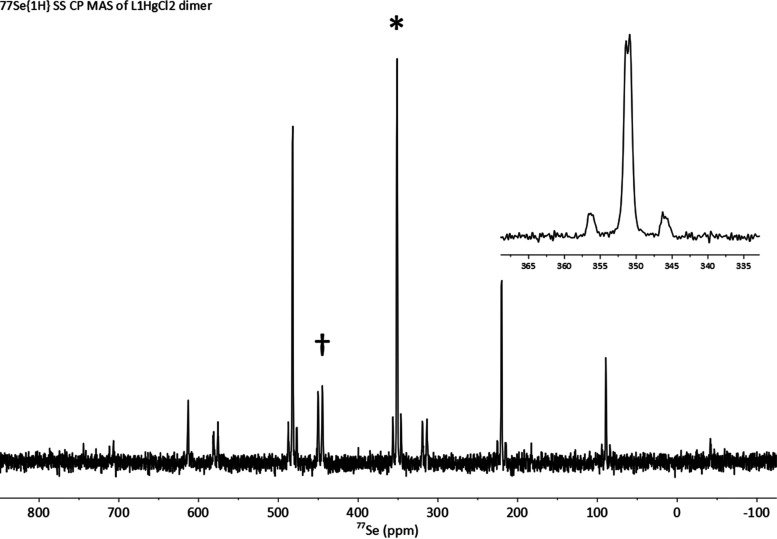
^77^Se{^1^H} SS NMR spectrum of **(L1HgCl**_**2**_**)**_**2**_ recorded at 76.3 MHz. The isotropic peak is located at δ_Se_ = 351.2 ppm and denoted with *. The impurity of **L1** is at δ_Se_ = 447.5 ppm and denoted with †.

To gain further insight into the nature of the Hg···Se interaction, a ^77^Se{^1^H} SS-MAS NMR spectrum of a sample of the dimeric complex **(L1HgCl**_**2**_**)**_**2**_ was acquired (Figure 6). The magnitude of *J*_SeHg_ obtained (785 Hz) showed only a marginal increase when compared with the magnitude observed for the solution of **L1HgCl**_**2**_ in *d*-chloroform (721.3 Hz). The similarity of the two *J*_SeHg_ magnitudes indicates that the mercury is bound relatively loosely to the selenium atom in chloroform solution, i.e., the bonding in the solution is similar to that observed in the crystal of the dimer **(L1HgCl**_**2**_**)**_**2**_. However, in both cases, a significant overlap of the Se and Hg orbitals still takes place to give rise to the observed high magnitudes of *J*_SeHg_. Not many examples of *J*_SeHg_ couplings have been reported in the literature,^31^ particularly those involving selenoethers. Those published span a large range of magnitudes. For example, a large magnitude of ^1^*J*_SeHg_ was reported in the Zintl anion [HgSe_2_]^2–^ (2258 Hz),^32^ while very weak bonding in a Hg(CN)_2_ complex of a crown selenoether (with Hg···Se distances 3.38–3.44 Å obtained from single-crystal X-ray diffraction) resulted in much smaller *J*_SeHg_ 110 and 123 Hz being observed in the ^77^Se CP-MAS SS NMR spectra.^33^ A magnitude particularly similar to that observed by us was recorded in the phosphine selenide complex Cl_2_Hg((Se =)P*n*Bu_3_)_2_ (^1^*J*_SeHg_ 751 ± 10 Hz); however, no structural data were reported for this complex.^34^

The solution state ^31^P{^1^H} and ^77^Se{^1^H} NMR spectra of **L2HgCl**_**2**_ show that increasing the steric bulk and the electron donating ability of the aryl group attached to the selenium atom results in an increase in the magnitudes of *J*_PSe_ (186.9 Hz in **L2HgCl**_**2**_*cf*. 86.7 Hz in **L1HgCl**_**2**_) and *J*_SeHg_ (909 Hz in **L2HgCl**_**2**_ cf. 721 Hz in **L1HgCl**_**2**_). The ^77^Se{^1^H} SS-MAS NMR spectrum of **L2HgCl**_**2**_ corresponds well with the solution state spectra, with only a small increase of the magnitude of *J*_SeHg_ in the solid state to ca. 1040 Hz (cf. 909 Hz in CDCl_3_), indicating that similar Hg–Se interactions exist in both solution and solid-state environments. A Hg–Se distance of 2.9132(5) Å was measured crystallographically in **L2HgCl**_**2**_ (see the structural discussion below). The bulkier **L3HgCl**_**2**_ and **L4HgCl**_**2**_ display *J*_PSe_ values comparable to that seen in **L2HgCl**_**2**_ (Table 2). Unfortunately, a high signal-to-noise ratio in the ^77^Se{^1^H} NMR spectra of **L3HgCl**_**2**_ and **L4HgCl**_**2**_ precluded the observation of ^199^Hg satellites and hence determination of the *J*_SeHg_ for these complexes.

**Figure 7:**
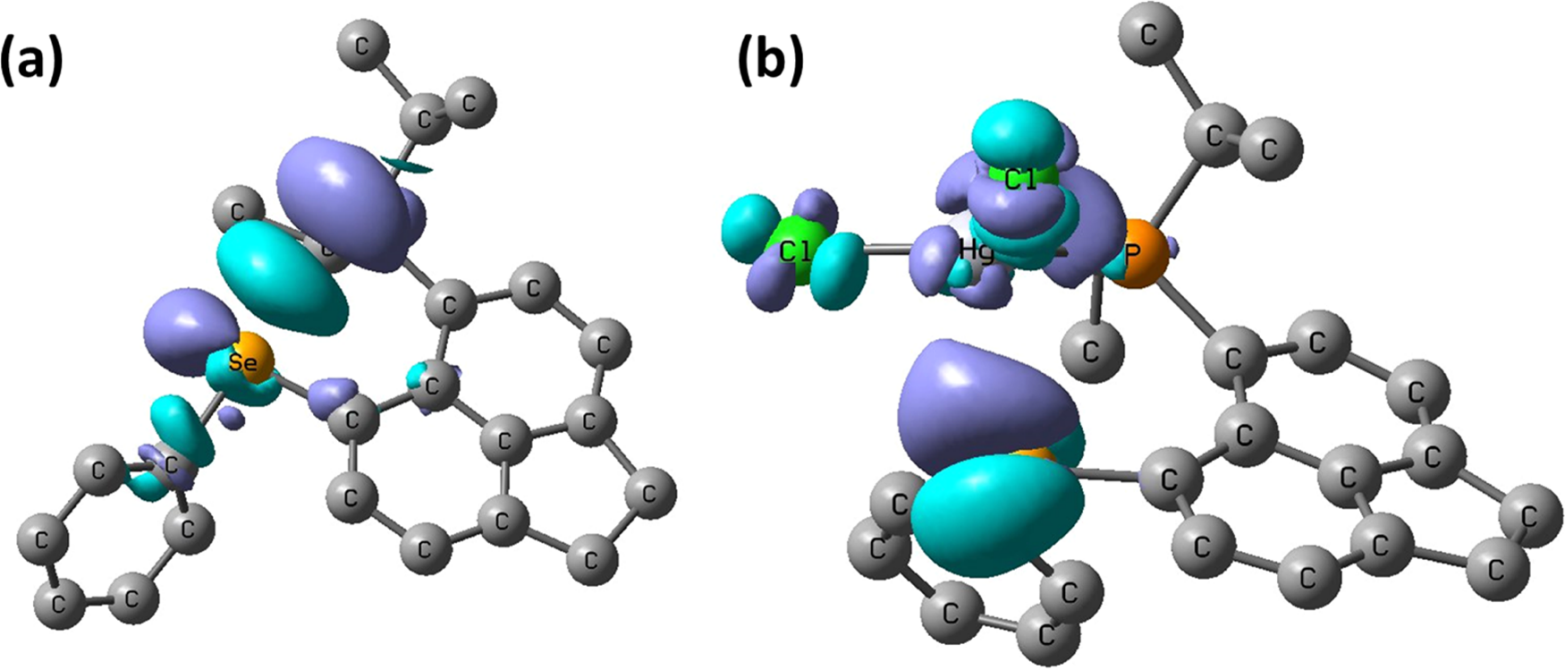
*J*_SeP_ coupling pathways visualized using the coupling deformation density (CDD, quasi-relativistic DFT level, BP86 functional, isosurfaces shown for a given cutoff value); (a) free **L1** (cutoff 0.55 au) and (b) **L1HgCl**_**2**_ (cutoff 0.08 au).

As indicated above, the *J*_PSe_ coupling in **L1** is diminished tremendously upon complexation to Hg (from 452 Hz in **L1** to 87 Hz in **L1HgCl**_**2**_). To gain additional understanding for this change, we performed density functional theory (DFT) calculations of these couplings and analyzed them with the coupling deformation density (CDD) approach.^35^ The heavy-metal complex calculations were performed at a suitable relativistic level (unrestricted 4-component Dirac–Kohn–Sham level, see the SI for details and references). The optimized geometries matched those obtained experimentally (from single-crystal diffraction) rather well. The computed coupling constants *J*_SeHg_, *J*_PHg_, and *J*_SeP_ show agreement only with the trends observed for the experimental ones, with some magnitudes being overestimated and others underestimated (see Table S3 in the SI). However, from the visualization of the corresponding CDD coupling paths (Figure 7), it is clear that in free **L1**, the coupling pathway is mainly through-space (note the large turquoise area between P and Se atoms in Figure 7a) and to a lesser extent along the P–C–C–C–Se bonds of the acenaphthene scaffold (as indicated by the much smaller contributions on these C atoms in Figure 7a). This finding is reminiscent of couplings involving heteroatoms that are formally nonbonded but forced in close proximity. This constraint imposed by the acenaphthene backbone can cause overlap of the lone pairs of the *peri*-atoms, leading to *J* couplings approaching or even exceeding ^1^*J* couplings between the same nuclei when they are covalently bound (see, for example, *J*_TeTe_ in Bühl et al.^36^). In contrast to the through-space *J*_SeP_ coupling in the free ligand, in the **L1HgCl**_**2**_ complex, this coupling is propagated predominantly through the P–Hg (optimized bond length 2.525 Å) and Hg–Se bonds (optimized bond length 2.878 Å). This is shown as the major contributions on the P, Hg, and Se atoms in Figure 7b. There is rather little direct through-space contribution (optimized P···Se distance 3.672 Å) and negligible propagation along the P–C–C–C–Se framework.

In summary, the NMR data indicate a significant interaction between the mercury and the selenoether moiety in these complexes; however, combining the single-crystal structural data with the solution and solid-state NMR highlights that the Hg–Se interaction is rather flexible. This applies, in particular, when the Hg–Se interaction is compared to the Hg–P interaction, which appears to be much more insensitive to the local environment.

**Figure 8:**
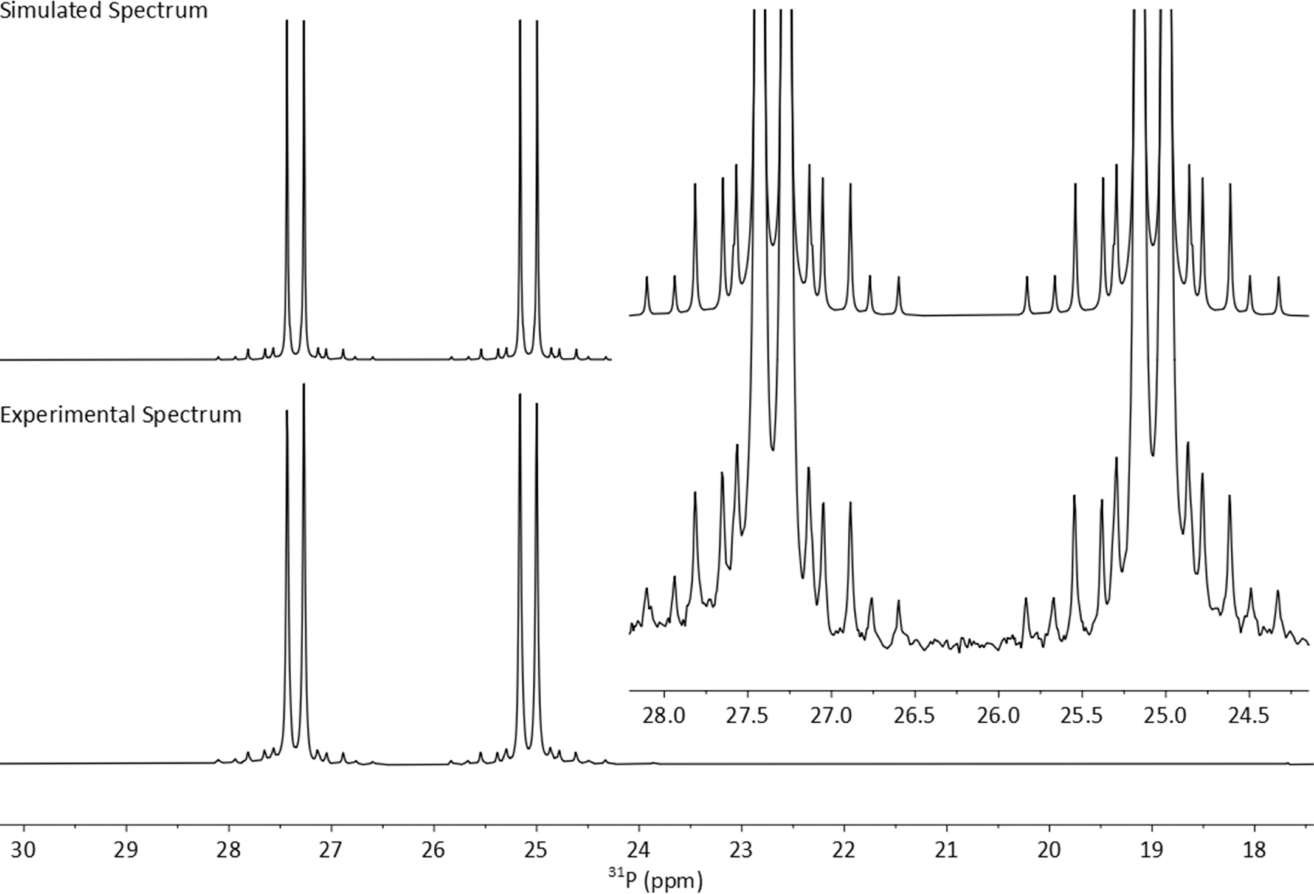
Simulated (top) and measured (bottom) ^31^P{^1^H} NMR spectra (202.5 MHz) of the **[L1**_**2**_**Ag]SbF**_**6**_ complex with expansions of the satellite peaks shown.

Silver has two naturally occurring isotopes, both of which have nuclear spin 1/2 with very similar natural abundancies, ^107^Ag (51.84%) and ^109^Ag (48.16%). Considering this and the presence of other NMR-active nuclei (^31^P and ^77^Se) in our complexes, the ^31^P{^1^H} and ^77^Se{^1^H} NMR spectra of **[L1**_**2**_**Ag]SbF**_**6**_ were expected to be rather complex. The ^31^P{^1^H} NMR spectrum shows two major doublets stemming from ^107^Ag and ^109^Ag isotopomers with NMR-inactive Se atoms. These doublets are both centered at δ_P_ 26.2 ppm, with ^1^*J*_31P–107Ag_ of 426.2 Hz and ^1^*J*_31P–109Ag_ of 494.0 Hz (Figure 8). These *J*_PAg_ magnitudes are significantly larger than those observed in the bis(phosphine) analogue **5** (see Figure 4, ^1^*J*_PAg_ couplings *i*Pr_2_P–Ag 249.1 and 287.5 Hz and Ph_2_P–Ag 172.9 and 200.6 Hz, respectively).^21^

**Figure 9:**
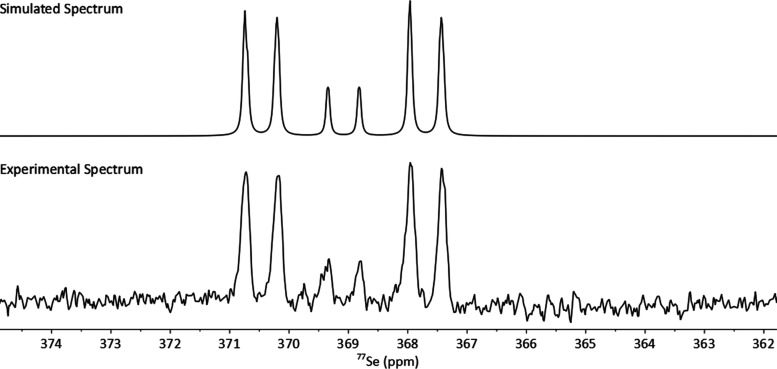
Simulated (top) and measured (bottom) ^77^Se{^1^H} NMR spectra (76.4 MHz) of the **[L1**_**2**_**Ag]SbF**_**6**_ complex.

**Figure 10:**
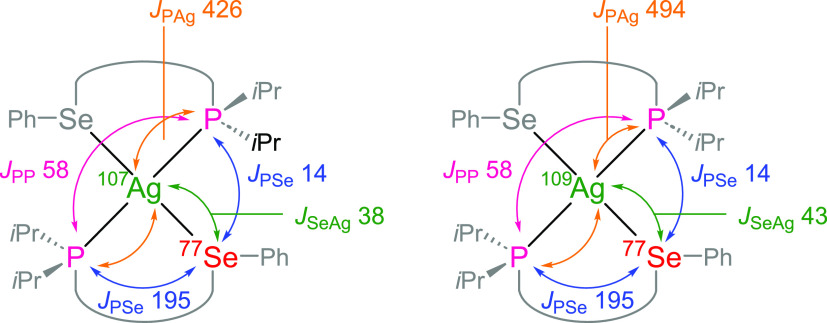
Coupling pathways for the two isotopomers of the [L1_2_Ag]SbF_6_ complex with ^107^Ag and ^109^Ag centers and one ^77^Se (NMR-active) atom; these form the complex satellite pattern. Two additional major isotopomers (not shown) with both Se atoms being NMR-inactive contribute to the major doublets seen in the ^31^P{^1^H} NMR spectra. The acenaphthene scaffold was simplified for clarity. Coupling constants are given in Hz.

For isotopologues with an NMR-active selenium atom (^77^Se, *I* = 1/2, 7.6% abundance), the spin system becomes dramatically more complicated, and a corresponding complex satellite pattern is observed in the ^31^P{^1^H} NMR spectrum (Figure 8). This is located at the heel of the major doublets, with some parts of the signals obscured by the major doublets. By carrying out a simulation of these spin systems, we were able to reproduce the experimental ^31^P{^1^H} and ^77^Se{^1^H} NMR spectra accurately as shown in Figures 8–10, with the latter being a complex multiplet centered at δ_Se_ 369.1 ppm. The spin simulations were also performed for the **[L2**_**2**_**Ag][Al(OC(CF**_**3**_**)**_**3**_**)**_**4**_**]** complex (see Figure S6 and Figure S7 in the SI).

The approximate magnitudes of the ^1^*J*_SeAg_ couplings of 43 and 38 Hz were obtained from the spin simulations of the ^77^Se{^1^H} NMR spectra of the two isotopomers of **[L1**_**2**_**Ag]SbF**_**6**_ with ^109^Ag and ^107^Ag atoms. These magnitudes are seemingly rather small; however, a literature search indicated that the observation of *J*_SeAg_ is rather unusual and seldom reported, as generally selenoether silver complexes give no observable couplings in the ^77^Se{^1^H} NMR spectra (with singlet signals only) even at low temperatures. The lack of observable one-bond Ag–Se couplings has been attributed to fast reversible ligand dissociation due to the extreme lability of the selenoether complexes. This was recorded for both aryl and alkyl selenoethers, such as in the complex [Ag(PhSeCH_2_CH_2_SePh)_2_]BF_4_^37^ and others.^38,39^ Observation of ^1^*J*_SeAg_ in both **[L1**_**2**_**Ag]SbF**_**6**_ and **[L2**_**2**_**Ag][Al(OC(CF**_**3**_**)**_**3**_**)**_**4**_**]** indicates increased chelate stability of the *peri*-P,Se geometry ligands compared to other more flexible geometries.

The ^31^P{^1^H} NMR spectrum of **L1BH**_**3**_ shows a broad singlet at δ_P_ 46.4 ppm with a line width of ca. 230 Hz, while the ^77^Se{^1^H} NMR spectrum shows a broad singlet at δ_Se_ 412.6 ppm with a smaller, yet broadened, line width of 56 Hz. The ^11^B{^1^H} NMR spectrum shows a broad doublet at δ_B_ −41.7 ppm, which allows for determination of approximate ^1^*J*_BP_ coupling of 35 Hz. Broadening of the signals precluded measurement of *J*_PSe_ and *J*_SeB_ couplings.

### Structural Investigations

**Figure 11:**
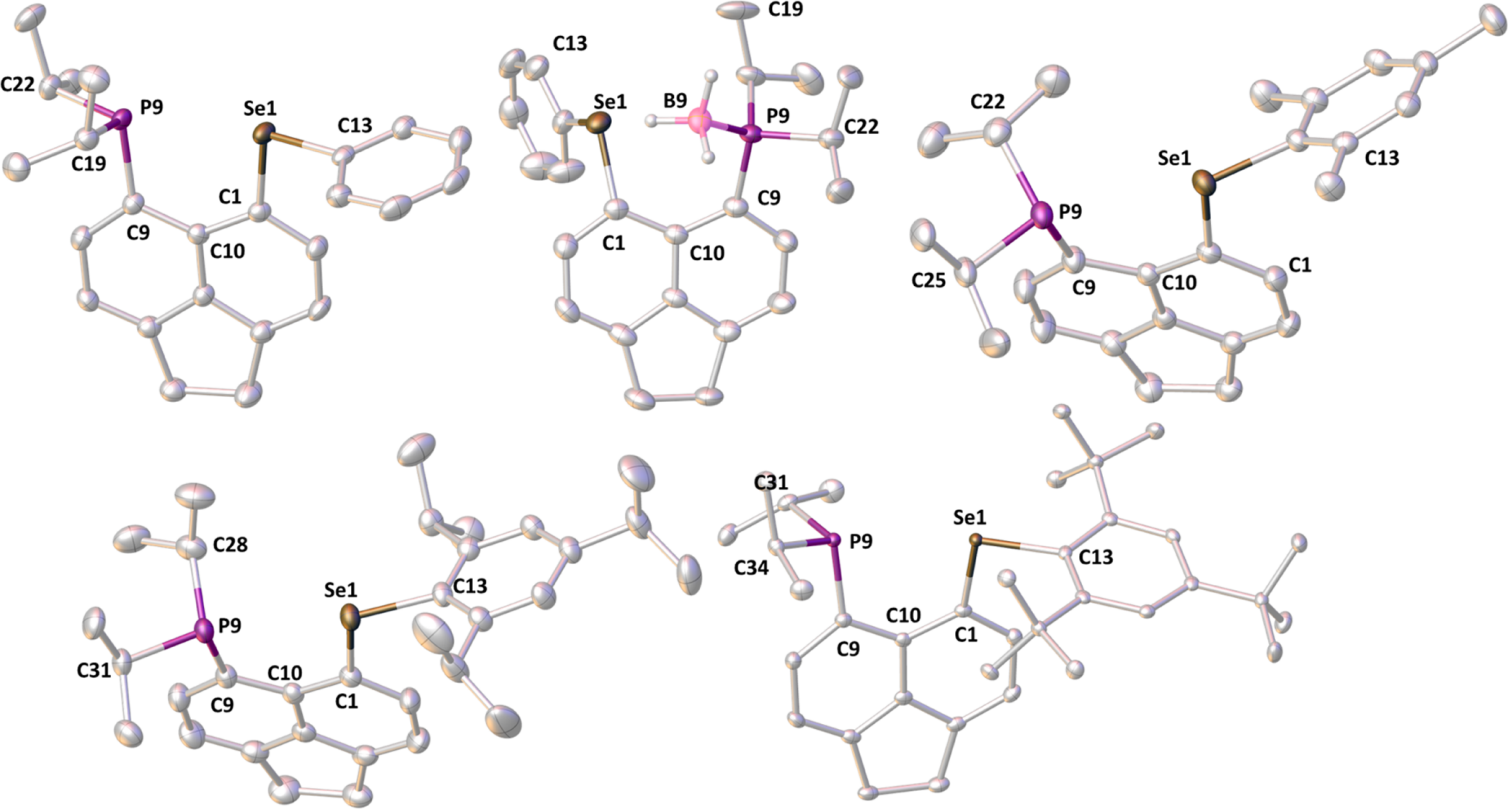
From left to right and top to bottom: molecular structures of **L1**, **L1BH**_**3**_, **L2**, **L3**, and **L4**. Carbon-bound hydrogen atoms are omitted for clarity. Anisotropic displacement ellipsoids are plotted at the 50% probability level.

**Figure 12:**
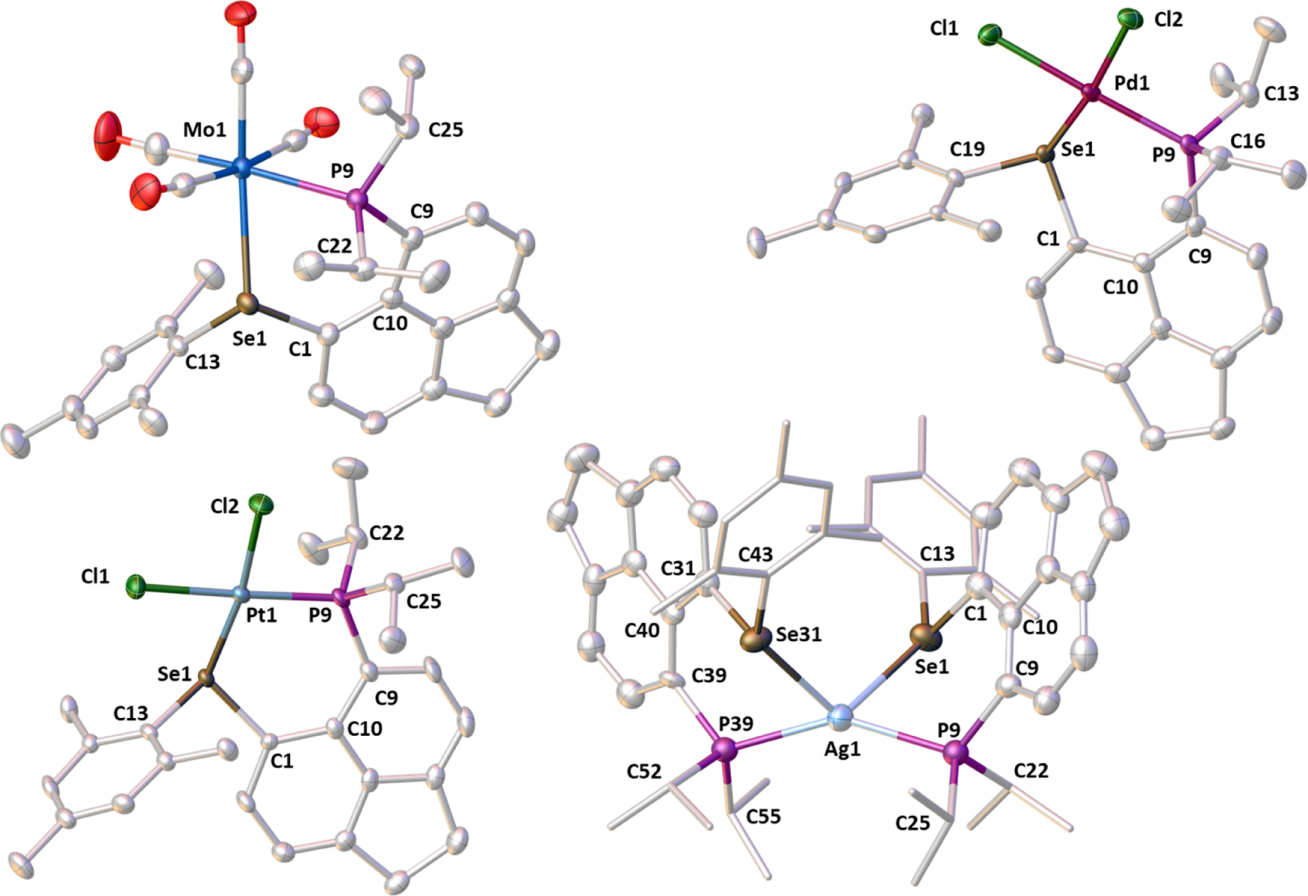
From left to right and top to bottom: molecular structures of **L2Mo(CO)**_**4**_, **L2PdCl**_**2**_, **L2PtCl**_**2**_, and **[(L2)**_**2**_**Ag][Al(OC(CF**_**3**_**)**_**3**_**)**_**4**_**]**. Counterions and hydrogen atoms are omitted for clarity. Anisotropic displacement ellipsoids are plotted at the 50% probability level, and peripheral groups are drawn as sticks only for the cation **[(L2)**_**2**_**Ag]**.

**Figure 13:**
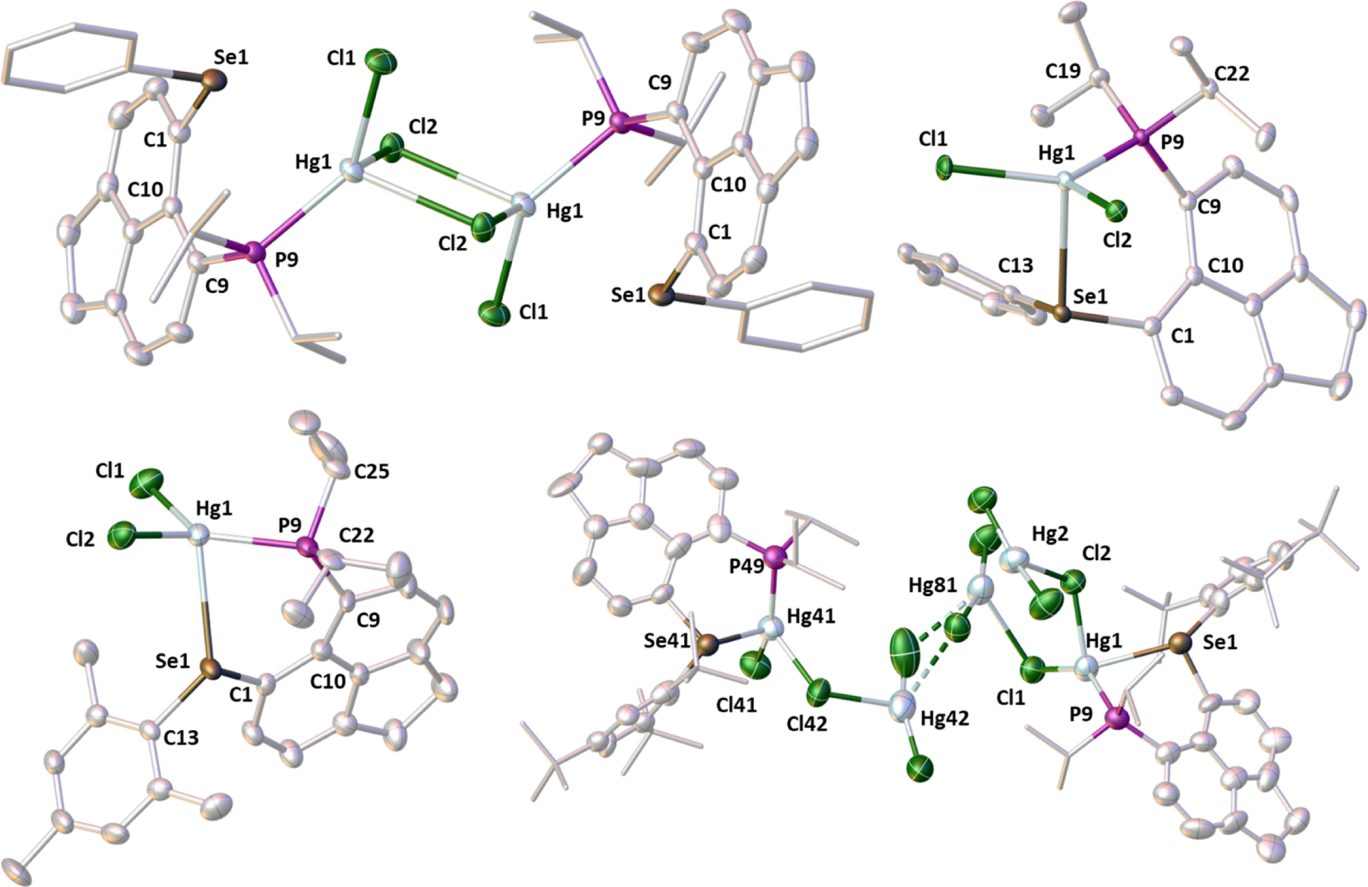
From left to right and top to bottom: molecular structures of **[L1HgCl**_**2**_**]**_**2**_, **L1HgCl**_**2**_, **L2HgCl**_**2**_, and **L4HgCl**_**2**_**·1.5HgCl**_**2**_. Solvating molecules and hydrogen atoms are omitted for clarity. Anisotropic displacement ellipsoids are plotted at the 50% probability level, and peripheral groups are drawn as sticks only.

All new compounds reported in this article (shown in Scheme 3), including ligands **L1**–**L4** but excluding complex **L3HgCl**_**2**_ were subjected to single-crystal X-ray diffraction studies. Selected crystallographic information is presented in Table 3, Figures 11–13, with additional information in the SI. In addition, diffraction data were also collected and solved for the intermediates **2Mes** and **2Tripp** and the side product Mes*SeBu. The data for the latter three compounds are listed in the SI but are not discussed in the main text.

**Table 3:** Selected Bond Distances (Ångströms, Å) and Angles (Degrees, °) for the Ligands and Metal Complexes

Compound	**L1** [DFT]^a^	**L2**	**L3**	**L4**	**L1BH**_**3**_
*peri*-region distances					
P9···Se1	3.055(1) [3.037]	3.135(1)	3.057(1)	3.076(1)	3.440(2)
*peri*-region bond angles					
P···Se–C(Ar)	165.3(1) [155.6]	160.8(1)	168.9(2)	170.0(1)	127.1(2)
splay^b^	12.5(4) [12.2]	14.5(3)	13.2(2)	10.7(2)	18.6(9)
out-of-plane displacements					
P9	0.287 [0.198]	0.299	0.147	0.428	0.571
Se	–0.153 [0.194]	–0.320	–0.210	–0.428	–0.700
*peri*-region torsion angle					
P–C···C–Se	10.9(2) [9.5]	14.7(1)	8.3(1)	20.6(1)	31.1(3)

Compound	**L1Mo(CO)**_**4**_	**L2Mo(CO)**_**4**_	**L1PtCl**_**2**_	**L2PtCl**_**2**_	**L2PdCl**_**2**_	**L4PdCl**_**2**_
*peri*-region distances						
P9···Se1	3.355(1)	3.186(1)	3.474(1)	3.384(1)	3.375(1)	3.257(1)
P9–M	2.5364(5)	2.5530(7)	2.2348(13)	2.2443(15)	2.2629(14)	2.2732(13)
Se1–M	2.6175(3)	2.6420(3)	2.3477(5)	2.3536(6)	2.3586(7)	2.3707(5)
*peri*-region bond angles						
P–M–Se	81.208(12)	75.635(16)	98.57(4)	94.76(4)	93.81(4)	89.04(3)
P···Se–C(Ar)	146.24(1)	163.59(2)	104.6(1)	136.2(1)	136.6(1)	157.2(1)
splay^b^	21.9(2)	16.2(3)	26.4(8)	22.6(8)	21.8(8)	18.8(6)
out-of-plane displacements						
P9	0.064	0.238	0.264	0.215	0.247	0.136
Se	–0.084	–0.206	–0.003	–0.369	–0.356	–0.390
M	1.372	1.569	0.415	0.484	0.520	0.665
*peri*-region torsion angle						
P–C···C–Se	3.6(1)	9.7(1)	5.9(3)	15.0(1)	15.9(1)	14.3(1)

Compound	**L1HgCl**_**2**_ [DFT]^a^	**[L1HgCl**_**2**_**]**_**2**_·2CHCl_3_	**L2HgCl**_**2**_	**[L4Hg**_**2**_**Cl**_**4**_**][L4Hg**_**3**_**Cl**_**6**_**]**^c^	**[(L1)**_**2**_**Ag]SbF**_**6**_·0.5CH_2_Cl_2_^d^	**[(L2)**_**2**_**Ag][Al(OC(CF**_**3**_**)**_**3**_**)**_**4**_**]**^d^
*peri*-region distances						
P9···Se1	3.762(1) [3.672]	3.423(1)	3.396(1)	3.377(5) [3.469(5)]	3.442(2) [3.260(3)]	3.226(2) [3.224(2)]
P9–M	2.4081(5) [2.525]	2.4305(7)	2.4323(11)	2.408(5) [2.418(5)]	2.428(3) [2.453(3)]	2.4621(17) [2.4669(17)]
Se1–M	2.8083(2) [2.878]	3.2100(4)	2.9132(5)	2.878(2) [2.983(2)]	2.8288(14) [2.7537(14)]	2.8806(9) [2.8564(9)]
*peri*-region bond angles						
P–M–Se	91.962(14) [85.4]	73.27(2)	78.33(3)	81.52(13) [76.61(13)]	81.41(7) [77.28(7)]	73.83(4) [74.15(4)]
P···Se–C(Ar)	85.88(7) [80.8]	101.2(1)	172.36(3)	176.7(1) [172.6(1)]	168.7(3) [154.0(4)]	170.8(1) [174.9(1)]
splay^b^	29.7(3) [25.7]	23.3(4)	20(1)	23(3) [23(3)]	21.1(16) [18.5(15)]	17.7(9) [17.4(9)]
out-of-plane displacements						
P9	0.734 [0.764]	0.185	0.539	0.181 [0.092]	0.596 [0.122]	0.258 [0.242]
Se	–0.361 [0.538]	–0.424	–0.215	–0.373 [−0.225]	–0.322 [−0.106]	–0.035 [0.234]
M	1.178 [1.261]	1.683	1.828	1.348 [1.608]	1.601 [1.829]	1.983 [1.819]
*peri*-region torsion angle						
P–C···C–Se	27.3(1) [31.7]	13.3(2)	19.8(2)	15.9(1) [4.9(1)]	23.5(7) [6.2(6)]	7.5(1) [10.8(1)]

a Values in square parentheses in italics are DFT PBE0-D3-optimized parameters.

b Splay angle = sum of the bay region angles −360.

c Values in square parentheses are for the second molecule in the asymmetric unit.

d Values in square parentheses are for the second P,Se-ligand molecule coordinated to silver.

The sum of the van der Waals radii of P and Se (∑*r*_vdW_ 3.85 Å) is much larger than the ideal *peri*-distance of ca. 2.5 Å.^30,40^ Despite incorporation of a large phosphine and a selenoether group in the *peri*-positions, the crystal structures of **L1** to **L4** (Figure 11) show only moderate in-plane and out-of-plane distortions. Interestingly, **L4**, bearing the bulkiest aryl group (Mes*), shows only slightly more pronounced out-of-plane distortions compared to the other three ligands (Table 3). The P···Se distances in **L1**–**L4** are rather similar in each of the ligands and range from 3.055(1) to 3.135(1) Å, i.e., 79 to 81% of ∑*r*_vdW_. On the other hand, these distances are significantly longer than ∑*r*_covalent_, for P and Se which is 2.27(7) Å.^29^ Due to the constrained geometry and mutual orientation of the two substituents as seen in Figure 11, a significant degree of overlap between the phosphorus and the selenium lone pairs is expected, and this is confirmed by large magnitudes of the observed ^4TS^*J*_PSe_, as mentioned in the NMR Spectroscopy section. A quasi-linear P···Se–C_Aryl_ arrangement with angles ranging from 160.8(1) to 170.0(1)° is present in all four ligands. This indicates the presence of a weak attractive chalcogen bond-like *n*(P) → σ*(Se–C_Ar_) orbital interaction.

The structure of **L1BH**_**3**_ (Figure 11 and Table 3) shows that the phosphine borane adduct is formed, with no bonding interaction between the boron and selenium atoms (interatomic separation of 3.256(8) Å).^41^ The splay angle of 18(1)° indicates a moderate amount of strain; in addition, there are significant out-of-plane displacements of the *peri*-atoms (ca. 0.57 and 0.70 Å) from the mean acenaphthene plane, and the P–C···C–Se torsion angle is 31.1(3)°. These suggest that the observed placement of the borane group within the *peri*-gap is due to minimized steric repulsion in such a configuration rather than an attractive interaction between selenium and boron atoms.

The crystal structures of the complexes **L2Mo(CO)**_**4**_, **L2PdCl**_**2**_, **L2PtCl**_**2**_, and **[(L2)**_**2**_**Ag][Al(OC(CF**_**3**_**)**_**3**_**)**_**4**_**]** are shown in Figure 12, and key details are given in Table 3. The structures of the related metal complexes with **L1** and **L4** ligands (**L1Mo(CO)**_**4**_, **L4PdCl**_**2**_, **L1PtCl**_**2**_, and **[(L1)**_**2**_**Ag]SbF**_**6**_) are rather similar. Their key details are displayed in Table 3, and the relevant figures are available in the SI (Figure S10). These complexes display κP,κSe coordination of the phosphino-selenoether ligands to the metals. The P–M distances in all reported complexes indicate tight bonding of the phosphine group to metals, with very little change of the bond lengths as the aryl group bulk on the selenium is varied. The geometries on the metal atoms in this study span (distorted) tetrahedral, square planar, and octahedral.

In both **L1Mo(CO)**_**4**_ and **L2Mo(CO)**_**4**_ the P,Se-ligands coordinate *cis* to the octahedral molybdenum center (Figure 12). The most noticeable changes upon coordination of the Mo(CO)_4_ fragment to **L1** are the increase in P···Se distance by ca. 0.3 Å and widening of the splay angle by ca. 9°. On the other hand, neither of these is changed significantly on coordination of **L2** to make **L2Mo(CO)**_**4**_. The Mo–Se distances of 2.6175(3) (**L1Mo(CO)**_**4**_) and 2.6420(3) Å (**L2Mo(CO)**_**4**_) are similar to those found in previously reported selenoether molybdenum complexes.^42,43^

In the platinum(II) and palladium(II) complexes **L1PtCl**_**2**_, **L2PtCl**_**2**_, **L2PdCl**_**2**_, and **L4PdCl**_**2**_, the metals adopt distorted square planar geometry. The P,Se-ligands adjust their geometry by a slight lengthening of the P···Se distances, ranging from 3.257(1) to 3.474(1) Å, and a moderate widening of the splay angle (ranging from 18.8(6) to 26.4(8)°). The Se–M (M = Pd, Pt) distances change only marginally as the selenium bound aryl group bulk increases; for example, the Se–Pd distance in **L4PdCl**_**2**_ is elongated only slightly when compared to **L2PdCl**_**2**_ (2.3707(5) vs 2.3587(7) Å). The Se–Pt bond lengths in **L1PtCl**_**2**_ (2.3477(5) Å) and **L2PtCl**_**2**_ (2.3536(6) Å) are very similar to those in the related phosphine selenoether complex **3** (2.3727(7) Å, Figure 4).^28^ On a similar note, the Se–Pd distances in **L2PdCl**_**2**_ (2.3587(7) Å) and **L4PdCl**_**2**_ (2.3707(5) Å) are as expected when compared to a related bis(selenoether) complex **6** (Pd–Se 2.3616(9) and 2.3514(9) Å, Figure 4).^44^

**Figure 14:**
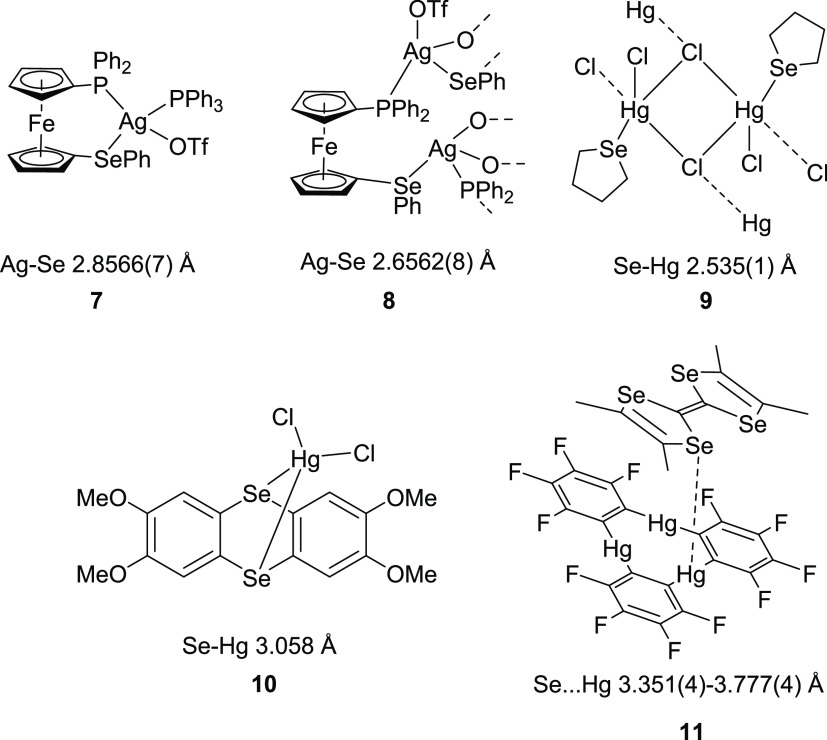
Literature complexes are mentioned in the discussion. Note that **8** is a chain polymer with bridging triflate anions, with each Ag atom coordinated by P, Se, and two O atoms. In **11**, the shortest Hg···Se contact is indicated by a dashed line.

In both silver(I) complexes, **[(L1)**_**2**_**Ag]SbF**_**6**_ and **[(L2)**_**2**_**Ag][Al(OC(CF**_**3**_**)**_**3**_**)**_**4**_**]**, two of the **L1** or **L2** ligands are coordinated to a single Ag atom in a κP,κSe fashion, adopting significantly distorted tetrahedral geometry around the silver atom. The crowding around the metal center results in the geometries of the two acenaphthene ligand molecules being rather different in **[(L1)**_**2**_**Ag]SbF**_**6**_ as indicated by (for example) large differences in P and Se out-of-plane displacements for the two ligands (see Table 3). The Ag–Se distances in **[(L1)**_**2**_**Ag]SbF**_**6**_ and **[(L2)**_**2**_**Ag][Al(OC(CF**_**3**_**)**_**3**_**)**_**4**_**]** (2.7539(16)–2.8806(9) Å) are comparable to those seen in tetrahedral complex **7** (2.8566(7) Å) and slightly elongated compared to that found in complex **8** (2.6562(8) Å) (Figure 14).^45^

An interesting structural variety is observed within the series of the four mercury complexes formed from ligands **L1**, **L2**, and **L4**. The complexes **L1HgCl**_**2**_, **L2HgCl**_**2**_, and **[L4Hg**_**2**_**Cl**_**4**_**][L4Hg**_**3**_**Cl**_**6**_**]** possess a four-coordinate mercury atom with an κP,κSe-bound P,Se-ligand and significantly distorted tetrahedral geometry. On the other hand, in **[L1HgCl**_**2**_**]**_**2**_·2CHCl_3_, the mercury atom is again four coordinate, the P,Se-ligand binding in a κP fashion, but with an additional loose P···Se interaction.

Both **L1HgCl**_**2**_ and **L2HgCl**_**2**_ adopt a monomeric structure with the HgCl_2_ motif spanning the two donor atoms of the P,Se-ligands (Figure 13). Attempts to crystallize **L4HgCl**_**2**_ gave a small amount of crystals that were shown to consist of the desired complex, with additional weakly coordinated HgCl_2_. The coordination geometry is similar to that found in **L1HgCl**_**2**_, with the HgCl_2_ motif bridging P and Se *peri*-atoms in **L4**; the additional weakly coordinated HgCl_2_ molecules form further weakly bridging interactions through the chlorine atoms, forming a structure of **[L4Hg**_**2**_**Cl**_**4**_**][L4Hg**_**3**_**Cl**_**6**_**]** (Figure 13 bottom right). Crystallization of **L1HgCl**_**2**_ from a different solvent (CHCl_3_) afforded crystals which were shown to adopt a dimeric structure **[L1HgCl**_**2**_**]**_**2**_·2CHCl_3_, with the chloride ligands forming Hg–(μCl)_2_–Hg bridges (Figure 13 top left).

Coordination to Hg(II) results in an increase of the P···Se distances in all four complexes (Δ = 0.261–0.707 Å); in the structurally divergent **[L1HgCl**_**2**_**]**_**2**_·2CHCl_3_, the increase was moderate at 0.368 Å. The P–Hg distances in all four complexes are consistent with a strongly coordinated phosphine group.

The varying Hg–Se distances observed within the Hg complexes were partly discussed in the NMR discussion above in connection with the observed *J*_SeHg_ couplings. In the complexes **L1HgCl**_**2**_, **L2HgCl**_**2**_, and **[L4Hg**_**2**_**Cl**_**4**_**][L4Hg**_**3**_**Cl**_**6**_**]**, the Hg–Se distances range from 2.8083(2) to 2.9132(5) Å, while in the complex **[L1HgCl**_**2**_**]**_**2**_·2CHCl_3_, the Hg···Se distance is elongated to 3.2100(4) Å, indicating a significantly weaker interaction.

A comprehensive literature search revealed that flexibility within the Hg–Se distances is a natural feature in selenoether mercury(II) complexes. Selected literature examples also point to a limited correlation between the coordination number of the Hg atom and the Hg–Se bond length. Thus, the Hg···Se distance in the five-coordinated complex **9** (2.535(1) Å)^46^ is much contracted compared to that in the four-coordinate complex **10** (3.058 Å) (see Figure 14).^47^ The weak bonding in the latter example is presumably (at least partially) a result of steric constraints imposed by the specific geometry of this particular bis(selenoether) ligand. Even longer (albeit still sub-van der Waals) Hg···Se distances were found in **11** (3.351(4)–3.777(4) Å), where *o*-phenylene mercury and tetraselenafulvalene components are both planar and form cofacial stacks, although the authors consider these as Hg···Se “contacts” rather than bonds.^48^ No NMR data have been reported for **11** unfortunately to allow comparison of its *J*_SeHg_ couplings with that in **[L1HgCl**_**2**_**]**_**2**_·2CHCl_3_.

Considering the literature precedents as well as our structural and spectroscopic data, it seems appropriate to view the **[L1HgCl**_**2**_**]**_**2**_·2CHCl_3_ complex as κP-bound and to consider the long Hg···Se contact as a weak secondary interaction rather than a standard coordination bond. It is interesting to note that despite this elongated Hg···Se distance, the (ligand-forced) proximity of the Hg and Se atom results in large magnitudes of *J*_SeHg_ in **[L1HgCl**_**2**_**]**_**2**_·2CHCl_3_, as observed in both solid-state NMR (785 Hz) and solution (721 Hz). The ease with which both the monomeric **L1HgCl**_**2**_ and dimeric **[L1HgCl**_**2**_**]**_**2**_·2CHCl_3_ forms were obtained indicates a close to equilibrium process. This somewhat resembles the halide-induced ligand conversion observed in complex **A4** (Figure 1), where addition/removal of chloride led to coordination/decoordination of the selenoether donor atom to the Pt(II) center.^10^ It appears our situation has a lower barrier as simple change of the solvent of crystallization induces the change.

## Conclusions

A series of phosphino and selenoether *peri*-substituted species **L1**–**L4** with varying bulk of the aryl substituent on selenium atom were synthesized. Coordination properties of these species were investigated in reactions with metal motifs Mo(0), Pt(II), Pd(II), Hg(II), and Ag(I) as well as BH_3_. In all but one case, the ligands coordinated in a κP,κSe bidentate manner. The exception was the mercury complex **[L1HgCl**_**2**_**]**_**2**_, which shows a monodentate κP coordination in the solid state with a rather long Se···Hg contact also present. Notwithstanding this, a notable *J*_SeHg_ (≥700 Hz) was observed in the solution and solid-state ^77^Se NMR spectra for this compound.

To provide further insight, we correlated NMR (solution and in some cases solid state) data with structural data as far as possible for this series of complexes.

The *peri*-substitution geometry with the two preorganized donor atoms (P and Se) appears to contribute to the overall fair stability of the studied complexes; all of these are air-stable and, apart from one of the mercury complexes, show no signs of (coordination/decoordination) fluxional behavior in the solution NMR spectra or variability of coordination modes in the solid state (as judged by single-crystal diffraction).

Preference for the formation of six-membered chelate rings (i.e κP,κSe coordination) is seen also in other phosphine-chalcoether peri-substituted ligands, namely, in the Cu^I^, Pt^II^, and Ru^II^ complexes of a P,S ligand (complexes **B2**, Figure 2)^11,14^ and in the Pt^II^ complex of a P,Te ligand, **B7**.^15^ On the other hand, the AuBr complex **B7** showed very elongated Se···Au interaction, consistent with very weak bonding (i.e., κP coordination only).^15^ It appears that the P,Se and P,Te ligands are therefore weaker-binding; however, because of a relatively small number of known metal complexes in each of the P,S, P,Se and P,Te ligand series, we hesitate to postulate a clear pattern in these chalcogeno-phosphine complexes.

Large magnitudes of through-space ^31^P–^77^Se couplings were observed in series **L1–L4** (452–545 Hz). This is due to the forced overlap of the lone pairs in the peri-region. A similar effect is observed in P,Te systems **B4**, with concomitant ^31^P–^125^Te couplings in a range of 1213 to 1357 Hz.^15^ Lack of any NMR-active isotope of sulfur precludes the observation of P–S couplings in the P,S species.

Quasi-linear P···Se–C_ipso_ arrangement with the angles ranging from 160.8(1) to 170.0(1)° is present in all four ligands **L1**–**L4**, and such quasi-linear P···Te–C_ipso_ geometry was also observed for all tellurium ligands **B4**.^15^ This is concomitant with a dative *n*(P) → σ*(Ch–C_Ar_) orbital interaction (i.e., intramolecular chalcogen bond), which, in turn, is believed to contribute to aligning the relevant orbitals to allow for significant through-space couplings mentioned above. By the way of contrast, great variety of geometries with respect to the orientation of the organyl group on the sulfur atom was observed in P,S ligands, with the P···S–C_ipso_ angles ranging from 84 to 165°.^12,13^

## Data Availability

The research data underpinning this publication can be accessed at 10.17630/c62c9350-b027-44f8-b553-b28cd7b4818f.

## Supporting Information Available

This information is available free of charge at the Web site: http://pubs.acs.org/ The Supporting Information is available free of charge at https://pubs.acs.org/doi/10.1021/acs.inorgchem.3c02255.

Full experimental details and analytical data, selected NMR spectra, spin system simulations, X-ray diffraction details and molecular structure figures, and computational details (PDF)
