# Supplementary material for: Phosphine and Selenoether peri-Substituted Acenaphthenes and Their Transition-Metal Complexes: Structural and NMR Investigations
Source: Inorg Chem. 2023 Sep 18;62(39):16084–100. doi: 10.1021/acs.inorgchem.3c02255 (PMC10548420; doi:10.1021/acs.inorgchem.3c02255)
Supplement: Supplementary file 1 — ic3c02255_si_001.pdf [file ic3c02255_si_001.pdf]

Supporting Information

Phosphine and Selenoether peri-Substituted Acenaphthenes and their Transition Metal Complexes: Structural and NMR Investigations

Lutao Zhang, Francesca A. Christie, Anna E. Tarcza, Helena G. Lancaster, Laurence J. Taylor, Michael Bühl, Olga L. Malkina, J. Derek Woollins, Cameron L. Carpenter-Warren, David B. Cordes, Alexandra M. Z. Slawin, Brian A. Chalmers and Petr Kilian**

Contents

Experimental....3
General Considerations....3
NMR Spectroscopy....3
Other Analyses....3
Synthesis of Diselenide Starting Materials ArSeSeAr....3
Dimesityl diselenide (Mes_2Se_2)....3
Bis((2,4,6-triisopropyl)phenyl) diselenide ($Tripp_2Se_2$)....4
Bis((2,4,6-tri-t-butyl)phenyl) diselenide ($Mes^*{}_2Se_2$)....4
Synthesis of Acenap(Br)(SeAr) Compounds....5
Synthesis of Acenap(Br)(SePh), 2Ph....5
Synthesis of Acenap(Br)(SeMes), 2Mes....5
Synthesis of Acenap(Br)(SeTripp), 2Tripp....6
Synthesis of Free Ligands L1-L4....6
Synthesis of Acenap(*i*Pr_2P)(SePh) L1....6
Synthesis of Acenap(*i*Pr_2P)(SeMes) L2....7
Synthesis of Acenap(*i*Pr_2P)(SeTripp) L3....7
Synthesis of Acenap(*i*Pr_2P)(SeMes*) L4 and isolation of a side-product *n*BuSeMes*....8
Synthesis of Metal Complexes....9
Synthesis of L1$PtCl_2$....9
Synthesis of L2$PtCl_2$....9

Synthesis of $L2PdCl_2$ 9
Synthesis of $L4PdCl_2$ 10
Synthesis of $L1Mo(CO)_4$ 10
Synthesis of $L2Mo(CO)_4$ 10
Synthesis of $[(L1)_2Ag]SbF_6$ 11
Synthesis of $[(L2)_2Ag][Al(OC(CF_3)_3)_4]$ 11
Synthesis of $L1HgCl_2$ and $[L1HgCl_2]_2$ 12
Synthesis of $L2HgCl_2$ 12
Synthesis of $L3HgCl_2$ 12
Synthesis of $L4HgCl_2$ 13
Synthesis of $L1BH_3$ 13
Further NMR Spectra and Spin Simulations 14
X-ray Diffraction 19
Computational Details 28
References 32

Experimental

General Considerations

Unless otherwise stated, all experimental procedures were carried out under an atmosphere of dry nitrogen using standard Schlenk techniques or under an argon atmosphere in a Saffron glove box. Dry solvents were used unless otherwise stated and were either collected from an MBraun SPS-800 Solvent Purification System, or dried and stored according to literature procedures.[1] Chemicals were purchased from Acros Organics, Alfa-Aesar, Sigma-Aldrich; or synthesised in-house using reagents from those suppliers. 2-bromo-1,3,5-tri-t-butylbenzene (Mes*Br)[2], and 5-bromo-6-(diispropylphosphino)acenapthene (**1**)[3] were synthesised according to literature procedures. "*In vacuo*" refers to a pressure of *ca.* 2×10^{-2} mbar.

NMR Spectroscopy

All novel compounds were characterised where possible by ^{1}H, $^{31}P\{^{1}H\}$ and $^{77}Se\{^{1}H\}$ NMR spectroscopy including measurement of $^{1}H\{^{31}P\}$, H-H DQF COSY, H-C HSQC, H-C HMBC and H-P HMBC. ^{13}C NMR spectra were recorded using the DEPTQ-135 pulse sequence with broadband proton decoupling. Measurements were performed at 20 °C using a Bruker Avance 300, Bruker Avance II 400 or Bruker Avance III 500 (MHz) spectrometer. For both ^{1}H and ^{13}C NMR, chemical shifts are relative to Me_4Si, which was used as an external standard. The residual solvent peaks were used for calibration ($CHCl_3$ δ_H 7.26, δ_C 77.2 ppm; benzene-d_6, δ_H 7.16 ppm, δ_C 128.1 ppm; dichloromethane-d_2, δ_H 5.32 ppm). The external standards of other nuclei used are as follows: ^{11}B – 15% $BF_3{\cdot}OEt_2$ in $CDCl_3$ (δ_B 0 ppm); ^{19}F − $CFCl_3$ (δ_F 0 ppm); ^{27}Al – 1.1 M $Al(NO_3)_3$ in D_2O {δ_{Al} 0 ppm); ^{31}P – 85% H_3PO_4 in D_2O (δ_P 0 ppm); ^{77}Se – Me_2Se and Ph_2Se_2 (δ_{Se} 0 and 463 ppm respectively); ^{195}Pt – 1.2 M $Na_2[PtCl_6]$ in D_2O (δ_{Pt} 0 ppm); ^{199}Hg – Me_2Hg and Ph_2Hg (δ_{Hg} 0 and −750 ppm respectively). Spin system simulations were performed using MestReNova 12.0.3, 2018.

The Solid-State $^{31}P\{^{1}H\}$ and $^{77}Se\{^{1}H\}$ NMR (SS-MAS NMR) measurements were performed using a Bruker Avance III 400 (MHz) spectrometer, operating at a magnetic field strength of 9.4 T. Experiments were carried out using conventional 4 mm MAS probes, with an MAS rate of 14 kHz for $^{31}P\{^{1}H\}$ and 10 kHz for $^{77}Se\{^{1}H\}$. The $^{77}Se\{^{1}H\}$ CP MAS experiments (using tamped contact pulse durations of 5–8 ms and TPPM ^{1}H decoupling) were carried out at 9.4 T. Chemical shifts are relative to Me_2Se at 0 ppm, using the isotropic resonance of solid H_2SeO_3, at 1288.1 ppm, as a secondary reference. The position of the isotropic resonance within the spinning sideband patterns were unambiguously determined by recording a second spectrum at a different MAS rate.

Other Analyses

Elemental analyses (C, H and N) were performed at London Metropolitan University. High Resolution Mass Spectrometry (APCI) was performed by the EPSRC UK National Mass Spectrometry Facility (NMSF) at Swansea University using a Waters Xevo G2-S mass spectrometer. Electrospray Ionisation (ES) was performed at the University of St Andrews Mass Spectrometry Facility using a Thermo Exactive Orbitrap Mass Spectrometer. Infrared spectra were collected on a Perkin Elmer 2000 NIR FT spectrometer using KBr discs in the range of 4000−400 cm^{-1}. Melting (or decomposition) points were determined by heating solid samples in glass capillaries using a Stuart SMP30 melting point apparatus and are uncorrected.

Synthesis of Diselenide Starting Materials ArSeSeAr

Dimesityl diselenide (Mes_2Se_2)

Synthesis was adapted from the method reported by Wang.[4]

Magnesium turnings (2.19 g, 90 mmol) and a trace amount of iodine were combined under a nitrogen atmosphere. To this, tetrahydrofuran (50 mL) was added. Subsequently, 2-bromomestiylene (11.91 g, 9.0 mL,

60 mmol) was added to the Mg/THF suspension. The mixture was heated to 50 °C to initiate the reaction. The suspension was stirred at 50 °C for 2 hours. Selenium powder (4.77 g, 60 mmol) was added to the mixture and the resulting suspension stirred overnight. The reaction was quenched by addition of 0.1 M aqueous hydrochloric acid (50 mL). This was extracted with diethyl ether (3 × 50 mL). The combined organic fractions were dried over magnesium sulfate. The suspension was filtered to remove any insoluble selenium powder that carried over. The volatiles were removed in vacuo to afford crude dimesityl diselenide as a yellow solid. The sample was recrystallised from boiling ethanol to afford yellow crystals (11.20 g, 94%). **^{1}H NMR:** δ_H (300.1 MHz, $CDCl_3$) 6.84 (4H, s, *m*-CH), 2.27 (6H, s, *p*-CH3), 2.23 (12H, s, *o*-CH_3). **^{13}C DEPTQ NMR:** δ_C (100.7 MHz, $CDCl_3$) 143.8 (s, *ipso*-qC), 139.3 (s, *p*-qC), 129.0 (s, *o*-qC), 128.5 (s, *m*-CH), 24.4 (s, *o*-CH_3), 21.3 (s, *p*-CH_3). **^{77}Se{^{1}H} NMR:** δ_{Se} (76.4 MHz, $CDCl_3$) 369.0 (s).

Bis((2,4,6-triisopropyl)phenyl) diselenide ($Tripp_2Se_2$)

Experimental procedure is identical to Mes_2Se_2 but using the following quantities. Mg powder (0.72 g, 30 mmol), 2-bromo-1,3,5-triisopropylbenzene (8.78 g, 7.0 mL, 30 mmol), selenium (2.37 g, 30 mmol). The product was obtained as orange crystals after recrystallisation from hot ethanol (3.62 g, 43%). **^{1}H NMR:** δ_H (500.1 MHz, $CDCl_3$) 6.90 (4H, s, *m*-CH), 3.53 (4H, hept, $^3J_{HH}$ 7.3 Hz, m-C$\underline{H}$(CH_3)$_2$), 2.82 (2H, hept, $^3J_{HH}$ 7.3 Hz, *p*-C$\underline{H}$(CH_3)$_2$), 1.20 (12H, d, $^3J_{HH}$ 6.9 Hz, *m*-CH(C$\underline{H_3}$)$_2$), 0.99 (24H, d, $^3J_{HH}$ 6.9 Hz, *p*-CH(C$\underline{H_3}$)$_2$). **^{13}C DEPTQ NMR:** δ_C (100.7 MHz, $CDCl_3$) 153.6 (s, *o*-qC), 150.3 (s, *p*-qC), 127.7 (s, *ipso*-qC), 121.7 (s, *m*-CH), 34.3 (s, *m*-$\underline{C}$H(CH_3)$_2$), 33.9 (s, *p*-$\underline{C}$H(CH_3)$_2$), 24.0 (s, *m*-CH($\underline{C}$H$_3$)$_2$), 23.9 (s, *p*-CH($\underline{C}$H$_3$)$_2$). **^{77}Se{^{1}H} NMR** δ_{Se} (95.4 MHz, $CDCl_3$) 357.2 (s). IR (KBr disk) ν_{max}/cm^{-1} 2959s (ν_{C-H}), 1460s, 1102m, 1055m, 877s.

Bis((2,4,6-tri-t-butyl)phenyl) diselenide ($Mes^*{}_2Se_2$)

Synthesis was adapted from the method reported by du Mont.[5]

A solution of Mes*Br (6.28 g, 19 mmol) in tetrahydrofuran and hexane (2:3 v/v, 100 mL) was cooled to −78 °C. To this, a solution of *n*-butyllithium (9 mL, 2.5 M solution in hexane, 21 mmol) was added dropwise over 1 hour. The mixture was stirred for a further 4 hours maintained at −78 °C. The volatiles were removed *in vacuo* and replaced with toluene (70 mL). This new solution was added to a cooled (−78 °C) suspension of selenium powder (1.66 g, 21 mmol) in toluene (10 mL) dropwise. The mixture was allowed to warm to ambient conditions with continuous stirring overnight. The mixture was opened and left under air (with continuous stirring) for a further 24 hours. The volatiles were removed *in vacuo* to afford a crude dark orange solid. This was purified by column chromatography using silica and petroleum ether as eluent (dry loading). The relevant fractions were identified, and volatiles removed *in vacuo* to afford $Mes^*{}_2Se_2$ as an orange solid (5.32 g, 78%). **^{1}H NMR:** δ_H (400.3 MHz, $CDCl_3$) 7.27 (4H, s, *m*-CH), 1.35 (36H, s, *o*-*t*Bu), 1.29 (18H, s, *p*-*t*Bu). **^{13}C DEPTQ NMR:** δ_C (100.7 MHz, $CDCl_3$) 155.9 (s, *ipso*-qC), 150.1 (s, *o*-qC), 150.0 (s, *p*-qC), 119.5 (s, *m*-CH), 35.0 (s, *o*-$\underline{C}$(CH_3)$_3$), 35.0 (s, *p*-$\underline{C}$(CH_3)$_3$), 31.6 (s, *o*-C($\underline{C}$H$_3$)$_3$), 31.3 (s, *p*-C($\underline{C}$H$_3$)$_3$). **^{77}Se{^{1}H} NMR:** δ_{Se} (76.4 MHz, $CDCl_3$) 514.8 (s).

Synthesis of Acenap(Br)(SeAr) Compounds

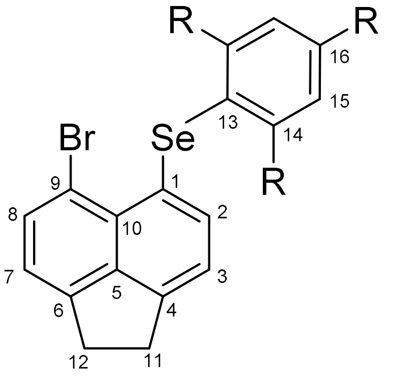

Figure S1: General NMR Numbering scheme for compounds Acenap(Br)(SeAr).

Synthesis of Acenap(Br)(SePh), 2Ph

Synthesis was adapted from the method reported by Woollins.[6]

To a cooled (−78 °C) suspension of 5,6-dibromoacenapthene (5.00 g, 16.0 mmol) in diethyl ether (40 mL), a solution of *n*-butyllithium (6.4 mL, 2.5 M solution in hexane, 16.0 mmol) was added dropwise over one hour. The suspension was stirred for a further hour at this temperature, after which a solution of diphenyl diselenide (5.00 g, 16.0 mmol) in diethyl ether (40 mL) was added over one hour. The resulting mixture was left to warm to ambient conditions with continuous stirring overnight. The work-up was carried out in air. The reaction was washed with aqueous sodium hydroxide (0.1 M, 3 × 50 mL). The organic fraction were dried over magnesium sulfate. The volatiles were removed *in vacuo* to yield crude Acenap(Br)(SePh) as an oil. The crude product was purified by column chromatography on silica gel using CH_2Cl_2:hexane (1:20 v/v). This afforded Acenap(Br)(SePh) as an off-white solid (4.25 g, 68%). Mp 79 °C (decomp.). **^{1}H NMR:** δ_H (500.1 MHz, $CDCl_3$) 7.72 (1H, d, $^3J_{HH}$ 7.4 Hz, H-8), 7.62–7.59 (2H, m, H-15), 7.39–7.33 (3H, m, H-14,16), 7.22 (1H, d, $^3J_{HH}$ 7.4 Hz, H-2), 7.10 (1H, d, $^3J_{HH}$ 7.4 Hz, H-7), 7.01 (1H, d, $^3J_{HH}$ 7.4 Hz, H-3), 3.32–3.26 (4H, m, H-11,12). **^{13}C DEPTQ NMR:** δ_C (125.8 MHz, $CDCl_3$) 146.9 (s, qC-6), 145.8 (s, qC-4), 142.1 (s, qC-5), 135.8 (s, C-15), 134.3 (s, C-8), 133.1 (s, C-2), 132.0 (s, *ipso*-qC-13), 130.3 (s, qC-10), 129.8 (s, C-14), 128.4 (s, C-16), 126.7 (s, qC-1), 120.9 (s, C-3), 120.8 (s, C-7), 115.4 (s, qC-9), 30.2 (s, C-11/12), 30.0 (s, C-11/12). **^{77}Se{^{1}H} NMR:** δ_{Se} (95.4 MHz, $CDCl_3$) 423.7 (s)

Synthesis of Acenap(Br)(SeMes), 2Mes

To a cooled (−78 °C) suspension of 5,6-dibromoacenapthene (0.62 g, 2.0 mmol), a solution of *n*-butyllithium (0.8 mL, 2.5 M solution in hexane, 2.0 mmol) diluted in diethyl ether (10 mL) was added dropwise over 30 minutes. The suspension was stirred for 3 hours at this temperature, after which a solution of dimesityl diselenide (0.79 g, 2.0 mmol) in diethyl ether (10 mL) was added over 30 minutes. The resulting mixture was left to warm to ambient conditions with continuous stirring overnight. The reaction was quenched with distilled water (25 mL) and was extracted with dichloromethane (3 × 25 mL). The combined organic fractions were dried over magnesium sulfate. The volatiles were removed *in vacuo* to yield crude Acenap(Br)(SeMes) as a pale-grey solid. The crude product was purified by column chromatography on silica gel using CH_2Cl_2:hexane (1:20 v/v). This afforded Acenap(Br)(SeMes) as an off-white solid (0.57 g, 66%). Mp 206–208 °C (decomp.). Crystals of **Acenap(Br)(SeMes)** of suitable quality for X-ray diffraction were grown by slow evaporation of a saturated solution in CH_2Cl_2 at ambient conditions. **Elemental Analysis:** Calcd. for $C_{21}H_{19}BrSe$: C, 58.62; H, 4.45. Found: C, 58.41; H, 4.34. **^{1}H NMR:** δ_H (400.3 MHz, $CDCl_3$) 7.71 (1H, d, $^3J_{HH}$ 7.4 Hz, H-8), 7.08 (1H, d, $^3J_{HH}$ 7.4 Hz, H-7), 7.05

(2H, s, H-15), 6.91 (1H, d, $^3J_{HH}$ 7.5 Hz, H-3), 6.75 (1H, d, $^3J_{HH}$ 7.5 Hz, H-2), 3.31–3.21 (4H, m, H-11,12), 2.45 (6H, s, *o*-CH_3), 2.35 (3H, s, *p*-CH_3). **^{13}C DEPTQ NMR:** δ_C (100.7 MHz, $CDCl_3$) 146.9 (s, qC-6), 144.4 (s, qC-13), 144.3 (s, qC-4), 142.5 (s, qC-5), 139.5 (s, qC-16), 133.9 (s, C-8), 130.4 (s, qC-10), 129.2 (s, C-15), 128.1 (s, C-2), 127.2 (s, q-C1), 121.2 (s, C-3), 120.7 (s, C-7), 115.6 (s, qC-9), 30.3 (s, C-11/12), 30.0 (s, C-11/12), 24.2 (s, *o*-CH_3), 21.4 (s, *p*-CH_3). **^{77}Se{^{1}H} NMR:** δ_{Se} (76.4 MHz, $CDCl_3$) 306.4 (s). **MS (ASAP)** m/z (%) 233.0 [42, M ($C_{21}H_{19}{}^{79}Br^{80}Se$)–SeMes + 2H], 310.9 (30, M–Mes), 431.0 (100, M + H). **HRMS (ASAP)** m/z Calcd. for $C_{21}H_{20}{}^{79}Br^{76}Se$: 426.9941, Found: 426.9937 [M+H]. **IR** (KBr disk) ν_{max}/cm^{-1} 2918s ($\nu_{C–H}$), 1597m, 1558m, 1409s, 1250s, 1113m, 1104m, 1019s, 845s, 808m. **FT-Raman** (glass capillary) ν_{max}/cm^{-1} 3204s ($\nu_{C–H}$), 1924m, 1841m, 1708m, 1664s, 1296m, 970m.

Synthesis of Acenap(Br)(SeTripp), 2Tripp

Experimental procedure is identical to Acenap(Br)(SeMes) but using the following quantities. 5,6-dibromoacenapthene (0.62 g, 2.0 mmol), *n*-butyllithium (0.8 mL, 2.5 M solution in hexane, 2.0 mmol) diluted in diethyl ether (10 mL); $Tripp_2Se_2$ (1.12 g, 2.0 mmol). The crude product was purified by column chromatography on silica gel using CH_2Cl_2/hexane (1:10 v/v) as the eluent. This afforded Acenap(Br)(SeTripp) as a light-yellow solid (0.56 g, 54%) Mp. 157.5–159.0 °C. Crystals of **Acenap(Br)(SeTripp)** of suitable quality for X-ray diffraction were grown by slow evaporation of a saturated solution in CH_2Cl_2 at ambient conditions. **^{1}H NMR:** δ_H (400.3 MHz, $CDCl_3$) 7.63 (1H, d, $^3J_{HH}$ 7.4 Hz, H-8), 7.07 (2H, s, H-17), 6.99 (1H, d, $^3J_{HH}$ 7.4 Hz, H-7), 6.84 (1H, d, $^3J_{HH}$ 7.5 Hz, H-3), 6.68 (1H, d, $^3J_{HH}$ 7.5 Hz, H-2), 3.63 (2H, hept, $^3J_{HH}$ 6.9 Hz, *o*-C<u>H</u>($CH_3)_2$), 3.21–3.13 (4H, m, H-11, H-12), 2.90 (1H, hept, $^3J_{HH}$ 6.9 Hz, *p*-C<u>H</u>($CH_3)_2$), 1.25 (6H, d, $^3J_{HH}$ 6.9 Hz, *p*-CH(C<u>H</u>$_3)_2$), 1.08 (12H, d, $^3J_{HH}$ 6.9 Hz, *o*-CH(C<u>H</u>$_3)_2$). **^{13}C DEPTQ NMR:** δ_C (100.7 MHz, $CDCl_3$) 154.2 (s, qC-6), 150.9 (s, qC-4), 146.9 (s, qC-15), 145 (s, qC-16), 142.5 (s, qC-5), 133.8 (s, C-8), 130.2 (s, qC-10), 129.4 (s, C-2), 129.0 (s, qC-1), 127.9 (s, qC-16), 122.5 (s, C-17), 121.0 (s, C-3), 120.6 (s, C-7), 115.7 (s, qC-9), 34.5 (s, *p*-<u>C</u>H($CH_3)_2$), 34.4 (s, *o*-<u>C</u>H($CH_3)_2$), 30.3 (s, C-11/12), 29.9 (s, C-11/12), 24.9 (s, *o*-CH(<u>C</u>H$_3)_2$), 24.2 (s, *p*-CH(<u>C</u>H$_3)_2$). **^{77}Se{^{1}H} NMR:** δ_{Se} (76.4 MHz, $CDCl_3$) 273.0 (s). **MS (ASAP)** m/z (%) 436.2 [10, M ($C_{27}H_{31}{}^{79}Br^{80}Se$) – Br], 515.1 (100, M + H). **HRMS (ASAP):** m/z Calcd. for $C_{27}H_{32}{}^{79}Br^{76}Se$: 511.0880, Found: 511.0877 [M+H]. **IR** (KBr disk) ν_{max}/cm^{-1} 2959s ($\nu_{C–H}$), 1459m, 1410s, 1360m, 1102m, 838s. **FT-Raman** (glass capillary) ν_{max}/cm^{-1} 3176m ($\nu_{C–H}$), 2928m ($\nu_{C–H}$), 1849m, 1801m, 1750s, 1696s, 1321m, 576m ($\nu_{C–Se}$).

Synthesis of Free Ligands L1-L4

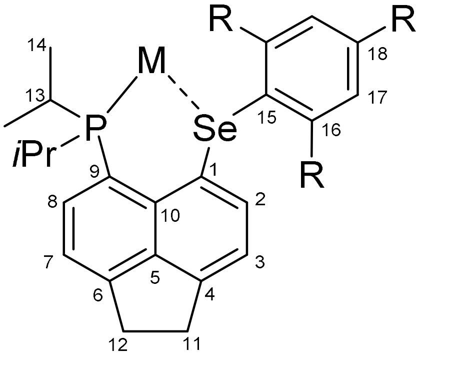

R = H, Me, *i*Pr, *t*Bu

Figure S2: General NMR Numbering scheme for compounds Acenap(iPr$_2$P)(SeAr)

Synthesis of Acenap(*i*Pr$_2$P)(SePh) L1

To a cooled (–78 °C), rapidly stirring solution of **1** (3.08 g, 8.8 mmol) in diethyl ether (80 mL), *n*-butyllithium (3.6 mL, 2.5 M solution in hexane, 8.8 mmol) was added dropwise over one hour. The mixture was allowed to stir for a further hour at this temperature. A solution of diphenyl diselenide (2.76 g, 8.8 mmol) in diethyl ether (40 mL) was added dropwise over 30 minutes with stirring maintained at –78 °C for a further hour before being allowed to warm to ambient temperature overnight. The volatiles were removed in vacuo and replaced with

toluene (80 mL). The suspension was washed with aqueous 0.1 M sodium hydroxide (150 mL) before drying the organic layer over magnesium sulfate. The volatiles were removed once more in vacuo to afford a pale yellow solid. The crude product was recrystallised from CH_2Cl_2/hexane (1:3 v/v) at −20 °C. The resulting precipitate was filtered and washed with ice-cold hexane (3 × 10 mL) affording **L1** as an analytically pure white solid (2.77 g, 74%) (Mp. 107–114 °C). Crystals of **L1** of suitable quality for X-ray diffraction were grown by slow evaporation of a saturated solution in CH_2Cl_2 at ambient conditions. **Elemental Analysis:** Calcd. (%) for $C_{24}H_{27}PSe$ (425.40): C 67.76, H 6.40; Found: C 67.70, H 6.31. **^{1}H NMR:** δ_H (499.9 MHz, $CDCl_3$) 7.73 (1H, dd, $^3J_{HH}$ 7.2, $^3J_{HP}$ 3.4 Hz, H-8), 7.67–7.61 (2H, m, H-17), 7.38–7.31 (4H, m, H-7, 16, 18), 7.20 (1H, d, $^3J_{HH}$ 7.2 Hz, H-2), 7.01 (1H, dt, $^3J_{HH}$ 7.2, $^4J_{HH}$ 1.5 Hz, H-3), 3.43–3.27 (4H, m, H-11,12), 2.27 (2H, dhept, $^3J_{HH}$ 7.0, $^2J_{HP}$ 3.7 Hz, H-13), 1.25 (6H, dd, $^3J_{HP}$ 14.3, $^3J_{HH}$ 7.0, 2 × CH_3, H-14), 1.02 (6H, dd, $^3J_{HP}$ 12.7, $^3J_{HH}$ 7.0 Hz, 2 × CH_3, H-14). **^{13}C DEPTQ NMR:** δ_C (75.5 MHz, $CDCl_3$), 148.4 (s, qC-6), 144.8 (s, qC-4), 140.3 (d, $^3J_{CP}$ 7.9 Hz, qC-5), 136.6 (d, $^2J_{CP}$ 26.8 Hz, qC-10), 136.0 (s, C-16), 136.0 (d, $^{5ts}J_{CP}$ 55.1 Hz, qC-15), 134.4 (s, C-8), 131.9 (s, C-2), 131.8 (s, qC-1), 130.2 (d, $^1J_{CP}$ 24.0 Hz, qC-9), 129.4 (s, C-17), 127.9 (s, C-18), 120.0 (s, C-3), 119.2 (s, C-7), 30.3 (s, C-11/12), 29.5 (s, C-11/12), 26.4 (d, $^1J_{CP}$ 15.3 Hz, C-13), 20.3 (d, $^2J_{CP}$ 11.5 Hz, 2 × CH_3, H-14), 20.1 (d, $^2J_{CP}$ 17.9 Hz, 2 × CH_3, H-14). **^{31}P{^{1}H} NMR:** δ_P (202.5 MHz, $CDCl_3$) −6.5 (s, with ^{77}Se satellites, $^{4ts}J_{PSe}$ 452.2 Hz). **^{77}Se{^{1}H} NMR:** δ_{Se} (95.4 MHz, $CDCl_3$) 425.3 (d, $^{4ts}J_{SeP}$ 452.8 Hz). **IR:** (KBr disc, cm^{-1}) ν = 3037m ($\nu_{Ar–H}$), 2922m ($\nu_{C–H}$), 1881m, 1603s, 1436vs, 1325vs, 1252s, 1038s, 836vs, 740vs, 469s. **HRMS:** (APCI+) *m/z* (%) Calcd. for $C_{24}H_{28}PSe$: 427.1089, found: 427.1089 (100) [M+H].

Synthesis of Acenap(iPr$_2$P)(SeMes) L2

To a cooled (–78 °C), rapidly stirring solution of **1** (0.69 g, 2.0 mmol) in diethyl ether (10 mL), *n*-butyllithium (0.8 mL, 2.5 M solution in hexane, 2.0 mmol) diluted in diethyl ether (10 mL) was added dropwise over one hour. The mixture was allowed to stir for a further hour at this temperature. A solution of dimesityl diselenide (0.79 g, 2.0 mmol) in diethyl ether (40 mL) was added dropwise over 30 minutes with stirring maintained at –78 °C for a further hour before being allowed to warm to ambient temperature overnight. Water (25 mL) was added, and the mixture extracted with dichloromethane (3 × 25 mL). The combined organic fractions were dried over magnesium sulfate. The volatiles were removed once more in vacuo to afford a pale brown solid. The crude product was recrystallised from CH_2Cl_2/hexane (1:3 v/v) affording **L2** as a pale-yellow solid (0.67 g, 72%) (Mp. 133–135 °C). Crystals of **L2** of suitable quality for X-ray diffraction were grown by slow evaporation of a saturated solution in CH_2Cl_2 at ambient conditions. **Elemental Analysis**: Calcd. (%) for $C_{27}H_{33}PSe$ (467.49): C 69.37, H 7.12; Found: C 69.19, H 7.21. **^{1}H NMR:** δ_H (500.1 MHz, $CDCl_3$) 7.62 (1H, dd, $^3J_{HH}$ 7.2, $^3J_{HP}$ 3.3 Hz, H-2), 7.22 (1H, d, $^3J_{HH}$ 7.2 Hz, H-3), 6.92 (2H, s, H-17), 6.84 (1H, d, $^3J_{HH}$ 7.4 Hz, H-7), 6.77 (1H, d, $^3J_{HH}$ 7.4 Hz, H-8), 3.32–3.14 (4H, m, H-11,12), 2.33 (6H, s, *o*-CH_3), 2.25 (3H, s, *p*-CH_3), 2.19 (2H, dhept, $^3J_{HH}$ 7.0, $^2J_{HP}$ 3.3 Hz, H-13), 1.17 (6H, dd, $^3J_{HP}$ 14.1, $^3J_{HH}$ 7.0 Hz, 2 × CH_3, H-14), 0.97 (6H, dd, $^3J_{HP}$ 12.8, $^3J_{HH}$ 7.0 Hz, 2 × CH_3, H-14). **^{13}C DEPTQ NMR:** δ_C (100.7 MHz, $CDCl_3$)148.6 (s, qC-4), 144.3 (s, qC-6), 143.6 (s, *ipso*-qC-15), 140.8 (d, $^3J_{CP}$ 7.8 Hz, qC-5), 138.7 (s, qC-16), 136.6 (d, $^2J_{CP}$ 26.8 Hz, qC-10), 134.2 (s, C-2), 133.7 (s, qC-18), 130.6 (d, $^1J_{CP}$ 24.9 Hz, qC-1), 129.1 (d, $^3J_{CP}$ 2.2 Hz, qC-9), 129.0 (s, C-17), 128.8 (s, C-8), 120.3 (s, C-7), 119.3 (s, C-3), 30.6 (s, C-11/12), 29.7 (s, C-11/12), 26.5 (d, $^1J_{CP}$ 15.7 Hz, C-13), 24.3 (s, *o*-CH_3), 21.4 (s, *p*-CH_3), 20.4 (d, $^2J_{CP}$ 12.1 Hz, 2 × CH_3, C-14), 20.1 (d, $^2J_{CP}$ 17.4 Hz, 2 × CH_3, C-14). **^{31}P{^{1}H} NMR:** δ_P (202.5 MHz, $CDCl_3$) −6.5 (s, with ^{77}Se satellites, $^{4TS}J_{PSe}$ 466.6 Hz). **^{77}Se{^{1}H} NMR:** δ_{Se} (95.4 MHz, $CDCl_3$) 315.7 (d, $^{4TS}J_{SeP}$ 466.4 Hz). **IR:** (KBr disk) ν_{max}/cm^{-1} 2918s (νC–H), 1598m, 1432m, 1322s, 845s (νC–P), 552w (νC–Se). **HRMS:** (ASAP) *m/z* (%) Calcd. for $C_{27}H_{34}PSe$: 463.1623, found: 463.1626. **IR** (KBr disk) ν_{max}/cm^{-1} 2918s ($\nu_{C–H}$), 1598m, 1432m, 1322s, 845s ($\nu_{C–P}$), 552w ($\nu_{C–Se}$). **FT-Raman** (glass capillary) ν_{max}/cm^{-1} 3452s ($\nu_{C–H}$), 3153m, 3083m, 1677w, 678m ($\nu_{C–P}$).

Synthesis of Acenap(iPr$_2$P)(SeTripp) L3

Experimental procedure is identical to **L1** but using the following quantities. **1** (1.54 g, 4.4 mmol) in diethyl ether (50 mL); n-butyllithium (1.8 mL, 2.5 M in hexane, 4.4 mmol); $Tripp_2Se_2$ (2.48 g, 4.4 mmol) in diethyl ether (20 mL); toluene (40 mL). The crude product was purified by column chromatography on silica using DCM/hexane

(1:10) as an eluent. **L3** was obtained as a white solid (3.48 g, 72%). Crystals of **L3** of suitable quality for X-ray diffraction were grown by slow evaporation of a saturated solution in CH_2Cl_2 at ambient conditions. **^{1}H NMR:** δ_H (300.1 MHz, $CDCl_3$) 7.70 (1H, dd, $^3J_{HH}$ 7.1, $^3J_{HP}$ 3.2 Hz, H-8), 7.31 (1H, d, $^3J_{HH}$ 7.2, H-3), 7.11 (2H, s, H-17), 6.94 (1H, d, $^3J_{HH}$ 7.4 Hz, H-7), 6.88 (1H, d, $^3J_{HH}$ 7.4 Hz, H-3), 3.72 (2H, hept, $^3J_{HH}$ 6.8 Hz, *o*-C$\underline{H}$(CH_3)$_2$), 3.39−3.24 (4H, m, H-11,12), 2.96 (1H, hept, $^3J_{HH}$ 6.5 Hz, *p*-C$\underline{H}$(CH_3)$_2$), 2.27 (2H, dhept, $^3J_{HH}$ 6.8, $^2J_{HP}$ 3.3 Hz, H-13), 1.32 (6H, d, $^3J_{HH}$ 6.9 Hz, *p*-CH(C$\underline{H}_3$)$_2$), 1.26 (6H, dd, $^3J_{HP}$ 13.9, $^3J_{HH}$ 6.9 Hz, 2 × CH_3, H-14), 1.20−1.04 (12H, br m, *o*-CH(C$\underline{H}_3$)$_2$), 1.08 (6H, dd, $^3J_{HP}$ 12.9, $^3J_{HH}$ 7.0 Hz, 2 × CH_3, H-14). **^{13}C DEPTQ NMR:** δ_C (100.7 MHz, $CDCl_3$) 153.2 (s, qC-4), 149.8 (s, qC-6), 148.4 (s, qC-15), 143.9 (s, qC-18), 140.6 (d, $^3J_{CP}$ 7.9 Hz, qC-5), 136.0 (d, $^2J_{CP}$ 27.0 Hz, qC-10), 133.8 (s, C-2), 132.4 (s, qC-16), 130.0 (s, C-8), 122.0 (s, C-17), 119.9 (s, C-7), 119.0 (s, C-3), 34.3 (s, *p*-$\underline{C}$H(CH_3)$_2$), 34.1 (s, *o*-$\underline{C}$H(CH_3)$_2$), 30.4 (s, C-11/12), 29.4 (s, C-11/12), 26.2 (d, $^1J_{CP}$ 16.3 Hz, C-13), 24. (s, *o*-CH($\underline{C}H_3$)$_2$), 24.0 (s, *p*-CH($\underline{C}H_3$)$_2$), 20.2 (d, $^2J_{CP}$ 12.4 Hz, 2 × CH_3, C-14), 19.8 (d, $^2J_{CP}$ 17.2 Hz, 2 × CH_3, C-14). **^{31}P{^{1}H} NMR:** δ_P (121.5 MHz, $CDCl_3$) −6.4 (s, with ^{77}Se satellites, $^{4TS}J_{PSe}$ 476.8 Hz). **^{77}Se{^{1}H} NMR:** δ_{Se} (57.3 MHz, $CDCl_3$) 283.8 (d, $^{4TS}J_{SeP}$ 476.8 Hz). **IR**: (KBr disk) ν_{max}/cm^{-1} 2959s (νC–H), 1593m, 1460s, 1381m, 1056m, 840m (νC–P), 421w (νC–Se). **MS** (ASAP) *m/z* (%) 553.3 [100, *M* ($C_{33}H_{45}P_{80}Se$) + H]. **HRMS:** (ASAP) m/z Calcd. for $C_{24}H_{33}PSe$: 547.2562, found: 547.2562 [M+H]. IR (KBr disk) ν_{max}/cm^{-1} 2959s ($\nu_{C–H}$), 1593m, 1460s, 1381m, 1056m, 840m ($\nu_{C–P}$).

Synthesis of Acenap(*i*Pr$_2$P)(SeMes*) L4 and isolation of a side-product *n*BuSeMes*

Experimental procedure is identical to **L1** but using the following quantities. **1** (2.79 g, 8.0 mmol) in diethyl ether (80 mL); n-butyllithium (3.2 mL, 2.5 M in hexane, 8.0 mmol); Mes*$_2$Se$_2$ (5.19 g, 8.0 mmol) in diethyl ether (40 mL); toluene (40 mL). **L4** and ***n*BuSeMes*** were obtained after purification by column chromatography on silica using hexane and then DCM/hexane (1:10) as eluents.

L4 was obtained as a white solid following recrystallisation from toluene (3.50 g, 74%) (Mp. 156.0-158.5 °C). Crystals of **L4** of suitable quality for X-ray diffraction were grown by slow evaporation of a saturated solution in acetonitrile at ambient conditions. **^{1}H NMR:** δ_H (400.1 MHz, $CDCl_3$) 7.70 (1H, dd, $^3J_{HH}$ 7.2, $^3J_{HP}$ 3.2 Hz, H-2), 7.52 (2H, s, H-17), 7.24 (1H, d, $^3J_{HH}$ 6.3 Hz, H-3), 6.79 (1H, d, $^3J_{HH}$ 7.6 Hz, H-7), 5.90 (1H, d, $^3J_{HH}$ 7.6 Hz, H-8), 3.36–3.16 (4H, m, H-11,12), 2.23 (2H, dhept, $^3J_{HH}$ 7.3, $^2J_{HP}$ 2.4 Hz, H-13), 1.51 (18H, s, *o*-C(C$\underline{H}_3$)$_3$), 1.38 (9H, s, *p*-C(C$\underline{H}_3$)$_3$), 1.24 (6H, dd, $^3J_{HP}$ 14.7, $^3J_{HH}$ 6.9 Hz, 2 × CH_3, H-14), 1.00 (6H, dd, $^3J_{HP}$ 12.2, $^3J_{HH}$ 6.8 Hz, 2 × CH_3. H-14). **^{13}C DEPTQ NMR:** δ_C (100.7 MHz, $CDCl_3$) 155.5 (s, qC-6), 150.5 (s, qC-18), 148.6 (s, qC-4), 143.1 (s, qC-16), 140.3 (s, qC-5), 136.1 (s, qC-10), 134.4 (s, C-2), 131.3 (s, qC-15), 129.6 (s, C-8), 123.2 (s, C-17), 119.9 (s, C-7), 118.9 (s, C-3), 39.2 (s, *o*-$\underline{C}$(CH_3)$_3$), 34.8 (s, *p*-$\underline{C}$(CH_3)$_3$), 32.6 (s, *o*-C($\underline{C}H_3$)$_3$), 31.6 (s, *p*-C($\underline{C}H_3$)$_3$), 30.5 (s, C-11/12), 29.4 (s, C-11/12), 26.9 (d, $^1J_{CP}$ 17.2 Hz, C-13), 20.6 (d, $^2J_{CP}$ 19.4 Hz, 2 × CH_3, C-14), 20.3 (d, $^2J_{CP}$ 12.1 Hz, 2 × CH_3, C-14). **^{31}P{^{1}H} NMR:** δ_P (162.0 MHz, $CDCl_3$) −6.0 (s, with ^{77}Se satellites, $^{4TS}J_{PSe}$ 545.0 Hz). **^{77}Se{^{1}H} NMR:** δ_{Se} (76.4 MHz, $CDCl_3$) 378.3 (d, $^{4TS}J_{SeP}$ 545.0 Hz). **MS:** (ASAP) m/z (%) 154.1 (100, Acenaphthene), 595.3 [39, M ($C_{36}H_{51}PSe$) + H]. **HRMS:** (ASAP) m/z Calcd. for $C_{36}H_{52}PSe$: 589.3026, found: 589.3009 [M+H]. FT-Raman (glass capillary) ν_{max}/cm^{-1} 3503s ($\nu_{C–H}$), 2911m ($\nu_{C–H}$), 2238m, 522m ($\nu_{C–P}$), 205s ($\nu_{C–Se}$).

n*BuSeMes was separated as one of the early fractions in the chromatographic purification on silica, using hexane as the eluent. Recrystallisation from hot ethanol gave ***n*BuSeMes*** as colourless crystals (0.72 g, 24%). Mp. 73.0 – 75.0 °C. **^{1}H NMR**: δ_H (400.1 MHz, $CDCl_3$) 7.37 (2H, s, 2 × aryl-CH), 2.46 (2H, t, $^3J_{HH}$ = 7.5 Hz, SeC*H*$_2$), 1.61 (18H, s, *t*Bu *ortho* to Se 6 × C*H*$_3$), 1.58 (2H, m, $^3J_{HH}$ = 7.6 Hz, SeCH$_2$C*H*$_2$), 1.35 (2H, m, $^3J_{HH}$ = 7.3 Hz, SeCH$_2$CH$_2$C*H*$_2$), 1.30 (9H, s, *t*Bu *ortho* to Se 6 × C*H*$_3$), 0.88 (2H, t, $^3J_{HH}$ = 7.3 Hz, CH$_2$C*H*$_3$). **^{13}C{^{1}H} NMR**: δ_C (101 MHz, $CDCl_3$) 154.9 (s, SeC*C* q*C*), 149.5 (s, *para* to Se q*C*), 127.6 (s, *ipso* to Se q*C*), 122.6 (s, *meta* to Se *C*H), 39.4 (s, *t*Bu *ortho* to Se q*C*), 36.0 (s, Se*C*H$_2$), 35.1 (s, *t*Bu *para* to Se q*C*), 32.8 (s, *t*Bu *ortho* to Se *C*H$_3$), 31.5 (s, *t*Bu *para* to Se *C*H$_3$), 31.0 (s, SeCH$_2$*C*H$_2$), 23.4 (s, SeCH$_2$CH$_2$*C*H$_2$), 13.9 (s, CH$_2$*C*H$_3$). **^{77}Se{^{1}H} NMR**: (95 MHz, $CDCl_3$) δ 217.4 (s). **FT-Raman** (glass capillary) ν_{max}/cm^{-1} 3806m, 3698s (νC–H), 1293w, 532m (νC–Se).

Synthesis of Metal Complexes

Synthesis of L1PtCl$_2$

A solution of L1 (200 mg, 470 µmol) in dichloromethane (20 mL) was prepared. With stirring dichloroido(1,5-cyclooctadiene)platinum(II) (176 mg, 470 µmol) was added in one go. The colourless solution was left to stir at ambient conditions overnight before the volatiles were removed *in vacuo* to give a pale-yellow powder, which was subsequently washed with ice-cold hexane (5 mL) to remove the 1,5-cyclooctadiene. The product was obtained as a white powder (219 mg, 67%). Crystals of **L1PtCl$_2$** of suitable quality for X-ray diffraction were grown by slow evaporation of its saturated solution in CH_2Cl_2 at ambient conditions. Mp. 214 °C (decomp.). **^{1}H NMR:** δ_H (300.1 MHz, $CDCl_3$) 8.06 (1H, dd, $^3J_{HH}$ 7.2 Hz, H-2), 7.92 (1H, dd, $^3J_{HP}$ 9.8, $^3J_{HH}$ 7.6 Hz, H-8), 7.52 (1H, d, $^3J_{HH}$ 7.6 Hz, H-7), 7.47 (1H, d, $^3J_{HH}$ 7.2 Hz, H-3), 7.19–7.05 (3H, m, H-16,18), 7.01–6.94 (2H, m, H-17), 3.65–3.46 (5H, m, H-11,12,13a), 2.81 (1H, br dh, $^2J_{HP}$ 11.6, $^3J_{HH}$ 7.0 Hz, H-13b), 1.44 (3H, dd, $^3J_{HP}$ 17.0, $^3J_{HH}$ 7.0 Hz, 1 × CH_3, H-14b), 1.03 (3H, dd, $^3J_{HP}$ 14.1, $^3J_{HH}$ 7.0 Hz, 1 × CH_3, H-14a), 0.80 (3H, dd, $^3J_{HP}$ 18.9, $^3J_{HH}$ 7.0 Hz, 1 × CH_3, H-14a'), 0.53 (3H, dd, $^3J_{HP}$ 18.1, $^3J_{HH}$ 7.0 Hz, 1 × CH_3, H-14b'). **^{13}C DEPTQ NMR**: δ_C (125.8 MHz, $CDCl_3$) 153.3 (s, qC-4), 153.2 (s, qC-6), 140.9 (d, $^3J_{CP}$ 7.2 Hz, qC-5), 138.2 (s, with ^{77}Se satellites, $^2J_{CSe}$ 39.9 Hz, C-2), 135.8 (d, $^2J_{CP}$ 7.6 Hz, qC-10), 135.0 (br s, C-8), 129.6 (s, qC-15), 129.6 (s, C-16), 129.2 (s, C-18), 128.9 (s, C-17), 120.6 (s, C-3), 119.7 (d, $^3J_{CP}$ 8.9 Hz, C-7), 111.0 (d, $^1J_{CP}$ 49.9 Hz, qC-9), 108.0 (d, $^3J_{CP}$ 7.8 Hz, with ^{77}Se satellites, $^1J_{CSe}$ 97.6 Hz, qC-1), 30.6 (s, C-11/12), 30.4 (s, C-11/12), 28.1 (d, $^1J_{CP}$ 36.3 Hz, C-13b), 27.5 (d, $^1J_{CP}$ 35.0 Hz, C-13a), 19.2 (s, 1 × CH_3, C-14a), 18.9 (s, 1 × CH_3, C-14b), 18.2 (s, 1 × CH_3, C-14b'), 16.8 (d, $^2J_{CP}$ 5.7 Hz, C-14a'). **^{31}P{^{1}H} NMR:** δ_P (121.5 MHz, $CDCl_3$) 10.0 (s, with ^{195}Pt satellites, $^1J_{PPt}$ 3528.5 Hz). **^{77}Se{^{1}H} NMR:** δ_{Se} (57.3 MHz, $CDCl_3$) 332.0 (s, with ^{195}Pt satellites, $^1J_{SePt}$ 656.3 Hz). **^{195}Pt{^{1}H} NMR**: δ_{Pt} (64.2 MHz, $CDCl_3$), –4190.0 (d, $^1J_{PtP}$ = 3528.4, with ^{77}Se satellites, $^1J_{PtSe}$ 656.6 Hz). **IR:** (KBr disc, cm^{-1}) ν = 3050m (ν_{C-H}), 2924s (ν_{C-H}), 1601vs, 1440s, 1327m, 1212m, 1037s, 916s, 850vs, 727vs, 629s, 530m. **HRMS** (APCI+) *m/z* (%) Calcd. for $C_{24}H_{27}PSePtCl$: 656.0358, found: 656.0372 (100) [M–Cl].

Synthesis of L2PtCl$_2$

Experimental procedure is identical to **L1PtCl$_2$** but using the following quantities. **L2** (94 mg, 200 µmol), [$PtCl_2$(COD)] (75 mg, 200 µmol), dichloromethane (10 mL). The product was obtained as a white powder (170 mg, 62%). Crystals of **L2PtCl$_2$** of suitable quality for X-ray diffraction were grown by slow evaporation of its saturated solution in CH_2Cl_2 at ambient conditions. **^{1}H NMR:** δ_H (500.1 MHz, $CDCl_3$) 8.07 (1H, dd, $^3J_{HP}$ 10.6, $^3J_{HH}$ 7.5 Hz, H-8), 7.45 (1H, d, $^3J_{HH}$ 7.5 Hz, H-2), 7.29 (1H, d, $^3J_{HH}$ 7.5 Hz, H-7), 7.14 (1H, d, $^3J_{HH}$ 7.5 Hz, H-3), 6.95 (2H, s, H-17), 4.31−4.19 (1H, m, 1 × *i*Pr H-13), 3.47−3.38 (4H, m, H-11,12), 2.65 (6H, s, *o*-CH_3), 2.62−2.56 (1H, m, 1 × *i*Pr H-13) 2.31 (3H, s, *p*-CH_3), 1.61–1.55 (3H, m, 1 × CH_3, H-14), 1.42 (3H, dd, $^3J_{HP}$ 13.5, $^3J_{HH}$ 7.2 Hz, 1 × CH_3, H-14), 1.21 (3H, dd, $^3J_{HP}$ 18.3, $^3J_{HH}$ 6.9 Hz, 1 × CH_3, H-14) 1.14 (3H, dd, $^3J_{HP}$ 18.8, $^3J_{HH}$ 7.1 Hz, 1 × CH_3, H-14). **^{31}P{^{1}H} NMR:** δ_P (202.5 MHz, $CDCl_3$) 10.8 (s, with ^{195}Pt satellites, $^1J_{PPt}$ 3419.2 Hz). **^{77}Se{^{1}H} NMR:** δ_{Se} (95.4 MHz, $CDCl_3$) 307.4 ($^2J_{SeP}$ 6.5 Hz, with ^{195}Pt satellites, $^1J_{SePt}$ 357.3 Hz).

Synthesis of L2PdCl$_2$

Experimental procedure is identical to **L1PtCl$_2$** but using the following quantities. **L2** (94 mg, 200 µmol), [$PdCl_2(NCMe)_2$] (62 mg, 240 µmol), dichloromethane (10 mL). The product was obtained as a yellow powder (120 mg, 93%). Crystals of **L2PdCl$_2$** of suitable quality for X-ray diffraction were grown by layering a saturated solution in CH_2Cl_2 with hexane at ambient conditions. Mp. 198–200 °C (decomp.).**^{1}H NMR:** δ_H (400.3 MHz, $CDCl_3$) 8.09 (1H, dd, $^3J_{HP}$ 9.8, $^3J_{HH}$ 7.6 Hz, H-8), 7.46 (1H, d, $^3J_{HH}$ 7.6 Hz, H-2), 7.28 (1H, d, $^3J_{HH}$ 7.5 Hz, H-7), 7.16 (1H, d, $^3J_{HH}$ 7.5 Hz, H-3), 6.96 (2H, s, H-17), 4.35–4.20 (1H, m, 1 × *i*Pr H-13), 3.49–3.35 (4H, m, H-11,12), 2.64 (6H, s, *o*-CH_3), 2.62−2.56 (1H, m, 1 × *i*Pr H-13) 2.29 (3H, s, *p*-CH_3), 1.65 (3H, dd, $^3J_{HP}$ 17.9, $^3J_{HH}$ 6.5 Hz, 1 × CH_3, H-14), 1.45 (3H, dd, $^3J_{HP}$ 13.7, $^3J_{HH}$ 7.1 Hz, 1 × CH_3, H-14), 1.30 (3H, dd, $^3J_{HP}$ 18.8, $^3J_{HH}$ 6.5 Hz, 1 × CH_3, H-14) 1.19 (3H, dd, $^3J_{HP}$ 19.4, $^3J_{HH}$ 6.8 Hz, 1 × CH_3, H-14). **^{13}C DEPTQ NMR**: δ_C (100.7 MHz, $CDCl_3$) 153.3 (s, qC-4), 150.2 (s, qC-6), 140.5 (d, $^3J_{CP}$ 6.7 Hz, qC-5), 134.6 (s, C-2), 133.6 (s, qC-15), 132.9 (s, *m*-CH), 121.0 (s, C-8), 119.2 (s, C-7), 119.1 (s, C-3), 31.4 (d, $^1J_{CP}$ 29.6 Hz, C-13b), 30.5 (s, C-11/12), 30.0 (s, C-11/12), 28.1 (d, $^1J_{CP}$ 36.3 Hz, C-13b), 27.6 (d, $^1J_{CP}$ 26.8

Hz, C-13a) 25.3 (s, *o*-CH_3), 21.3 (s, *p*-CH_3) 20.3 (s, 1 × CH_3, C-14a), 20.0 (s, 1 × CH_3, C-14b), 19.3 (s, 1 × CH_3, C-14b'), 18.4 (d, $^2J_{CP}$ 8.9 Hz, C-14a'). **$^{31}P\{^1H\}$ NMR:** δ_P (202.5 MHz, $CDCl_3$) 38.1 (s). **$^{77}Se\{^1H\}$ NMR:** δ_{Se} (95.4 MHz, $CDCl_3$) 324.1 (s). **HRMS** (ESI+) m/z Calcd. for $C_{27}H_{33}PSePdCl$ 609.0208, found: 609.0203 [M−Cl].

Synthesis of L4PdCl$_2$

Experimental procedure is identical to **L2PdCl$_2$** but using the following quantities. **L4** (120 mg, 200 µmol), [$PdCl_2(NCMe)_2$] (62 mg, 240 µmol), dichloromethane (2 mL). The product was obtained as a dark orange powder (110 mg, 70%). Crystals of **L4PdCl$_2$** of suitable quality for X-ray diffraction were grown by layering a saturated solution in CH_2Cl_2 with hexane at ambient conditions. Mp. 183−185 °C (decomp.). **^1H NMR:** δ_H (400.3 MHz, $CDCl_3$) 8.07 (1H, dd, $^3J_{HP}$ 9.5, $^3J_{HH}$ 7.6 Hz, H-8), 7.60 (2H, s, H-17), 7.46 (1H, d, $^3J_{HH}$ 7.5 Hz, H-2), 7.18 (1H, d, $^3J_{HH}$ 7.5 Hz, H-7), 6.91 (1H, d, $^3J_{HH}$ 7.5 Hz, H-3), 3.51−3.37 (6H, m, H-11,12,13), 1.63 (18H, s, *o*-$C(CH_3)_3$), 1.44−1.33 (21H, m, 5 × CH_3, *p*-$C(CH_3)$, C-14). **^{13}C DEPTQ NMR**: δ_C (100.7 MHz, $CDCl_3$) 153.2 (s, qC-4), 152.1 (s, qC-6), 149.3 (s, qC-15), 134.0 (s, C-2), 131.7 (s, C-8), 120.8 (s, C-17), 119.1 (s, C-3), 119.0 (s, C-7), 40.3 (s, *o*-$\underline{C}(CH_3)_3$), 34.6 (s, *o*-$C(\underline{C}H_3)_3$), 31.4 (s, *p*-$C(CH_3)_3$), 30.6 (s, C-11/12), 30.0 (s, C-11/12), 29.9 (d, $^1J_{CP}$ 28.0 Hz, C-13), 28.0 (*p*-$\underline{C}(CH_3)_3$) 19.3 (br s, C-14). **$^{31}P\{^1H\}$ NMR:** δ_P (162.0 MHz, $CDCl_3$) 36.4 (s, with ^{77}Se satellites, $^2J_{PSe}$ 24.5 Hz). **$^{77}Se\{^1H\}$ NMR:** δ_{Se} (57.3 MHz, $CDCl_3$) 411.5 (d, $^2J_{SeP}$ 24.5 Hz). **HRMS** (ESI+) m/z Calcd. for $C_{36}H_{51}PSePdCl$ 735.1611, found: 735.1612 [M−Cl].

Synthesis of L1Mo(CO)$_4$

A solution of **L1** (191 mg, 450 µmol) in dichloromethane (20 mL) was prepared. With stirring, a solution of tetracarbonyldipiperidinemolybdenum(0) (170 mg, 450 µmol) in dichloromethane (8 mL) was added dropwise over 10 minutes. The colourless solution was left to stir at ambient conditions overnight before the volatiles were removed *in vacuo* and the residual oil was purified by column chromatography on silica (hexane:ethyl acetate 9:1 v/v). The product was obtained as a pale green powder (126 mg, 44%). Crystals of **L1Mo(CO)$_4$** of suitable quality for X-ray diffraction were grown by slow evaporation of its saturated solution in CH_2Cl_2 at ambient conditions. Mp. 173 °C (decomp.). **Elemental Analysis:** Calcd. (%) for $C_{28}H_{27}O_4PSeMo$ (633.39): C 53.10, H 4.30; Found: C 52.99, H 4.28. **^1H NMR:** δ_H (300.1 MHz, $CDCl_3$) 7.76 (1H, dd~t, $^3J_{HP}$ 7.6, $^3J_{HH}$ 7.6 Hz, H-2), 7.65−7.56 (2H, m, H-17), 7.43−7.39 (4H, m, H-2,16,18), 7.09 (1H, d, $^3J_{HH}$ 7.5 Hz, H-8), 7.52 (1H, d, $^3J_{HH}$ 7.6 Hz, H-7), 7.47 (1H, d, $^3J_{HH}$ 7.2 Hz, H-3), 3.47−3.35 (4H, m, H-11,12), 2.67−2.47 (2H, m, H-13), 1.30−1.21 (12H, m, H-14). **^{13}C DEPTQ NMR**: δ_C (75.5 MHz, $CDCl_3$) 219.7 (d, $^2J_{CP}$ 7.6 Hz, qC-$\underline{C}$O), 214.9 (d, $^2J_{CP}$ 25.6 Hz, qC-$\underline{C}$O), 209.2 (d, $^2J_{CP}$ 9.3 Hz, qC-$\underline{C}$O), 150.7 (s, qC-6), 149.4 (s, qC-4), 141.7 (d, $^3J_{CP}$ 7.1 Hz, qC-5), 137.5 (d $^1J_{CP}$ 7.9 Hz, C-9), 135.2 (s, qC-15), 134.9 (s, C-16), 132.9 (s, C-8), 132.2 (s, C-17), 127.9 (s, C-18), 129.1 (s, C-2), 122.7 (d, $^3J_{CP}$ 2.6 Hz, qC-1), 122.3 (d, $^2J_{CP}$ 18.9 Hz, qC-10), 119.6 (s, C-3), 119.0 (d, $^3J_{CP}$ 5.2 Hz, C-7), 30.1 (s, C-11/12), 29.8 (s, C11/12), 28.0 (d, $^{1f}J_{CP}$ 6.9 Hz, C-13a), 27.8 (d, $^1J_{CP}$ 5.1 Hz, C-13b), 18.7(d, $^2J_{CP}$ 3.0 Hz, 2 × CH_3, C-14), 18.3 (d, $^2J_{CP}$ 5.9 Hz, 2 × CH_3, C-14). **$^{31}P\{^1H\}$ NMR**: δ_P (162.0 MHz, $CDCl_3$) 40.8 (s, with ^{77}Se satellites, $^2J_{PSe}$ 14.7 Hz). **$^{77}Se\{^1H\}$ NMR**: δ_{Se} (76.4 MHz, $CDCl_3$) 394.1 (d, $^2J_{SeP}$ 14.8 Hz). **HRMS** (ES+): m/z (%) Calcd. for $C_{24}H_{28}MoPSe$: 525.0152, found: 525.0137 [M−4CO+H].

Synthesis of L2Mo(CO)$_4$

Experimental procedure is identical to **L1Mo(CO)$_4$** but using the following quantities. **L2** (47 mg, 100 µmol), [$Mo(CO)_4(pip)_2$] (45 mg, 120 µmol), dichloromethane (2 mL). The product was purified on silica using CH_2Cl_2:hexane 1:10 v/v. The product was obtained as green needles after slow diffusion of hexane into a saturated CH_2Cl_2 solution (27 mg, 36%). Mp. 172−175 °C (decomp.). **Elemental Analysis:** Calcd. (%) for $C_{31}H_{33}O_4PSeMo$ (675.347): C 55.12, H 4.92; Found: C 54.97, H 5.05. **^1H NMR:** δ_H (400.3 MHz, $CDCl_3$) 7.71 (1H, dd~t, $^3J_{HP}$ 7.6, $^3J_{HH}$ 7.6 Hz, H-2), 7.39 (1H, d, 3JHH 7.4 Hz, H-7), 7.23 (1H, d, 3JHH 7.4 Hz, H-2), 7.06 (1H, d, 3JHH 7.4 Hz, H-3), 7.01 (2H, s, H-17), 3.47–3.32 (4H, m, H-11,12), 2.63−2.51 (2H, m, H-13), 2.38 (6H, s, *o*-CH_3), 2.36

(3H, s, *p*-CH_3), 1.39 (6H, dd, $^3J_{HP}$ 15.7, $^3J_{HH}$ 6.8 Hz, 2 × CH_3, H14), 1.27 (6H, dd, $^3J_{HP}$ 15.1, $^3J_{HH}$ 6.9 Hz, 2 × CH_3, H-14). **^{13}C DEPTQ NMR**: δ_C (100.6 MHz, $CDCl_3$) 219.9 (d, $^2J_{CP}$ 7.7 Hz, qC-C̲O), 215.2 (d, $^2J_{CP}$ 25.8 Hz, qC-C̲O), 209.3 (d, $^2J_{CP}$ 8.7 Hz, qC-C̲O), 150.3 (s, qC-4), 148.3 (s, qC-6), 140.8 (s, qC-16), 139.5 (s, qC-18), 131.7 (s, C-2), 130.8 (s, C-8), 130.2 (s, C-17), 122.6 (s, qC-9), 119.6 (s, C-7), 118.9 (s, C-3), 30.1 (s, C-11/12), 29.7 (s, C-11/12), 27.7 (d, $^1J_{CP}$ 15.9 Hz, C-13), 23.2 (s, *o*-CH_3), 21.0 (s, *p*-CH_3), 19.3 (d, $^2J_{CP}$ 4.6 Hz, 2 × CH_3, C-14), 17.8 (d, $^2J_{CP}$ 4.8 Hz, 2 × CH_3, C-14). **^{31}P{^{1}H} NMR**: δ_P (162.0 MHz, $CDCl_3$) 42.3 (s, with ^{77}Se satellites, $^2J_{PSe}$ 37.0 Hz). **^{77}Se{^{1}H} NMR**: δ_{Se} (76.4 MHz, $CDCl_3$) 284.7 (d, $^2J_{SeP}$ 37.0 Hz).

Synthesis of $[(L1)_2Ag]SbF_6$

In darkness, under an atmosphere of dry nitrogen, a colourless solution of silver(I) hexafluoroantimonate (81 mg, 0.24 mmol) in dichloromethane (7 mL) was prepared. This was added dropwise to a solution of **L1** (200 mg, 0.47 mmol) in dichloromethane (7 mL) over a few minutes. The solution was left to stir at ambient conditions overnight before being filtered through a bed of celite to give a colourless solution. The volatiles were removed *in vacuo* to afford an air stable white solid of analytical purity (259 mg, 92%) (Mp. 173 °C with decomposition). Crystals of **$[(L1)_2Ag)]SbF_6$** of suitable quality for X-ray diffraction were grown by slow evaporation of its saturated solution in CH_2Cl_2 at ambient conditions. **Elemental Analysis:** Calcd. (%) for $C_{48}H_{54}P_2Se_2AgSbF_6$ (1194.43): C 48.27, H 4.56; Found: C 48.09, H 4.42. **^{1}H NMR:** δ_H (500.1 MHz, $CDCl_3$) 7.73 (1H, dd ~ t, $^3J_{HH} \approx {}^3J_{HP}$ 7.1 Hz, H-8), 7.47 (1H, d, $^3J_{HH}$ 7.1 Hz, H-7), 7.16 (1H, t, $^3J_{HH}$ 7.5 Hz, H-18), 7.08 (2H, br s, H-2,3), 6.87 (2H, t, $^3J_{HH}$ 7.5 Hz, H-17), 6.73 (2H, t, $^3J_{HH}$ 7.5 Hz, H-16), 3.54–3.34 (4H, m, H-11,12), 2.66 (2H, br s, H-13), 1.42–1.26 (6H, m, 2 × CH_3, H-14), 1.24–1.09 (6H, m, 2 × CH_3, H-14). **^{13}C DEPTQ NMR**: δ_C (125.8 MHz, $CDCl_3$) 151.6 (s, qC-6), 148.5 (s, qC-4), 141.7 (br s, qC-5), 135.7 (s, C-8), 134.7 (s, C-2), 134.5 (br s, qC-10), 133.4 (s, C-16), 129.8 (s, C-17), 129.7 (br s, qC-15), 128.8 (s, C-18), 122.6 (s, qC-1), 122.3–122.0 (m, $^1J_{CP}$ = 10.0 Hz, qC-9), 121.2 (s, C-3), 120.2 (s, C-7), 30.3 (s, C-11/12), 29.8 (s, C-11/12), 26.6 (br s, C-13), 21.3 (br s, 2 × CH_3, C-14), 18.9 (br s, 2 × CH_3, C-14). **^{31}P{^{1}H} NMR**: δ_P (202.5 MHz, $CDCl_3$) AA′XX′MM′ spin system (A/A′ = *i*Pr_2P; X = ^{107}Ag; X′ = ^{109}Ag; M/M′ = SeMes) centred at 26.2 ppm ($^1J_{AX}$ 426.2, $^1J_{AX'}$ 494.0 Hz). **^{77}Se{^{1}H} NMR**: δ_{Se} (76.4 MHz, $CDCl_3$) 368.9 (m). **HRMS:** (APCI) *m/z* (%) Calcd. for $C_{48}H_{54}AgP_2Se_2$: 959.1092, found: 959.1087 (55) [M–SbF_6]; Calcd. for $C_{24}H_{27}PSeAg$: 533.0066, found: 533.0053 (100) [M–L–SbF_6].

Synthesis of $[(L2)_2Ag][Al(OC(CF_3)_3)_4]$

In darkness, a suspension of $Ag[Al(OC(CF_3)_3)_4]$ (110 mg, 100 µmol) in dichloromethane (10 mL) was cooled to 0 °C. To this, a solution of L2 (47 mg, 100 µmol) in dichloromethane (20 mL) was added dropwise. The mixture was warmed to ambient conditions and stirred for 48 hours. The volatiles were removed *in vacuo* and the resulting solid washed with ethanol (3 mL) to give an off-white solid (84 mg, 42%). Mp. 120−122 °C (decomp.). Crystals of **$[(L2)_2Ag)](Al(OC(CF_3)_3)_4)$** of suitable quality for X-ray diffraction were grown by slow evaporation of its saturated solution in CH_2Cl_2 at ambient conditions. **^{1}H NMR:** δ_H (500.1 MHz, $CDCl_3$) 7.65 (2H, dd~t, $^3J_{HH} \approx {}^3J_{HP}$ 7.6 Hz, H-8), 7.40 (2H, d, $^3J_{HH}$ 7.3 Hz, H-7), 6.97 (2H, d, $^3J_{HH}$ 7.5 Hz, H-2), 6.77 (4H, s, H-15), 6.74 (2H, d, $^3J_{HH}$ 7.5 Hz, H-3), 3.48–3.31 (8H, m, H-11,12), 2.79 (4H, br s, H-13), 2.23 (12H, s, *o*-CH_3), 2.17 (6H, s, *p*-CH_3), 1.36 (24H, dd, $^3J_{HP}$ 15.9 $^3J_{HH}$ 6.9 Hz, H-14). **^{13}C DEPTQ NMR: δ_C** (125.1 MHz, $CDCl_3$) 151.9 (s, qC-6), 147.0 (s, qC-18), 142.4 (s, qC-16), 141.7 (m, qC-5), 140.4 (s, qC-15), 134.9 (d, $^2J_{CP}$ 3.2 Hz, C-8), 134.7 (m, qC-10), 130.7 (s, C-2), 129.7 (s, C-17), 123.8 (s, qC-1), 121.7 (m, qC-9), 121.5 (s, C-3), 121.4 (q, $^1J_{CF}$ 291.5 Hz, CF_3), 119.9 (s, C-7), 30.5 (s, C-11/12), 29.7 (s, C-11/12), 23.3 (s, CH in *i*Pr), 21.5-21.3 (m, *p*-CH_3 and CH_3 in *i*Pr), 21.1 (s, *o*-CH_3). **^{19}F NMR:** δ_F (376.5 MHz, $CDCl_3$) −75.5 (s). **^{27}Al NMR:** δ_{Al} (104.3 MHz, $CDCl_3$) 34.5 (s). **^{31}P{^{1}H} NMR:** δ_P (202.5 MHz, $CDCl_3$) AA′XX′MM′ spin system (A/A′ = *i*Pr_2P; X = ^{107}Ag; X′ = ^{109}Ag; M/M′ = SeMes) centred at 27.2 ppm ($^1J_{AX}$ 439.0, $^1J_{AX'}$ 506.1 Hz). **^{77}Se{^{1}H} NMR:** δ_{Se} (95.4 MHz, $CDCl_3$) 256.8 (m). **HRMS** (APCI) *m/z* (%) Calcd. for $C_{54}H_{66}AgP_2Se_2$: 1043.2033, found: 1043.2013 [M–anion].

Synthesis of L1HgCl$_2$ and [L1HgCl$_2$]$_2$

To a suspension of **L1** (196 mg, 461 µmol) in ethanol (20 mL) a colourless solution of mercury(II) chloride (125 mg, 461 µmol) in ethanol (8 mL) was added slowly. The suspension was left to stir at ambient conditions overnight and the solid was collected by filtration as a white powder (265 mg, 82%). Crystals of **L1HgCl$_2$** of suitable quality for X-ray diffraction were grown by slow evaporation of a saturated solution of **L1HgCl$_2$** in CH_2Cl_2 at ambient conditions. Crystals of the dimeric structure **[L1HgCl$_2$]$_2$** were obtained in the same way, except from $CHCl_3$. (M.p. 204 °C (decomp.). **Elemental Analysis:** Calcd. (%) for $C_{24}H_{27}PSeHgCl_2{\cdot}CHCl_3$ (816.28): C 36.79, H 3.46; Found: C 37.06, H 3.59. **^{1}H NMR:** δ_H (400.3 MHz, $CDCl_3$) 7.85 (1H, d, $^3J_{HH}$ 7.4 Hz, H-2), 7.74 (1H, dd, $^3J_{HP}$ 11.4, $^3J_{HH}$ 7.4 Hz, H-8), 7.46 (1H, d, $^3J_{HH}$ 7.3 Hz, H-7), 7.39–7.32 (3H, m, H-3,17), 7.30–7.25 (3H, m, H-16,18), 3.52 (4H, br s, H-11,12), 2.85–2.73 (2H, m, H-13) 1.37 (6H, dd, $^3J_{HP}$ 19.1, $^3J_{HH}$ 6.9 Hz, 2 × CH_3, H-14), 1.25 (6H, dd, $^3J_{HP}$ 19.5, $^3J_{HH}$ 7.1 Hz, 2 × CH_3, H-14). **^{13}C DEPTQ NMR**: δ_C (100.7 MHz, $CDCl_3$) 154.5 (s, qC-6), 150.9 (s, qC-4), 142.1 (s, C-2), 142.0 (s, qC-5), 137.9 (s, $^3J_{CHg}$ 85.0 Hz, C-8), 134.2 (d, $^2J_{CP}$ 8.9 Hz, qC-10), 130.6 (s, C-17), 130.0 (s, C-16), 128.2 (s, C-18), 122.1 (s, C-3), 120.3 (d, $^3J_{CP}$ 8.9 Hz, C-7), 118.5 (s, qC-1), 117.1 (d, $^1J_{CP}$ 37.8 Hz, qC-9), 30.5 (s, C-11/12), 30.0 (s, C-11/12), 27.9 (d, $^1J_{CP}$ 21.4, $^2J_{CHg}$ 78.7 Hz, C-13), 19.8 (d, $^2J_{CP}$ 2.3 Hz, 2 × CH_3, C-14), 18.7 (br s, 2 × CH_3, C-14). **^{31}P{^{1}H} NMR**: δ_P (162.0 MHz, $CDCl_3$) 54.0 (s, with ^{77}Se and ^{199}Hg satellites, $^1J_{PHg}$ 6610.7, $^2J_{PSe}$ 86.7 Hz). **^{77}Se{^{1}H} NMR**: δ_{Se} (76.4 MHz, $CDCl_3$) 378.3 (d, $^2J_{SeP}$ 87.5, with ^{199}Hg satellites $^1J_{SeHg}$ 721.3 Hz). **^{77}Se{^{1}H} CP MAS NMR**: δ_{Se} (9.4 T, 10 kHz) 351.2 (d, $^2J_{SeP}$ 37.5 Hz, with ^{199}Hg satellites, $^1J_{SeHg}$ 785.0 Hz). **^{199}Hg{^{1}H} NMR**: δ_{Hg} (89.5 MHz, $CDCl_3$) –621.7 (d, $^1J_{HgP} \approx$ 6800 Hz). **HRMS** (APCI) *m/z* (%) Calcd. for $C_{24}H_{27}ClPSeHg$: 661.0396, found: 661.0386 (40) [M–Cl], Calcd. for $C_{24}H_{28}PSeO_2$: 459.0992, found: 459.0981 (100) [M–$HgCl_2$+O_2H].

Synthesis of L2HgCl$_2$

To a suspension of **L2** (47 mg, 100 µmol) in dichloromethane (1 mL) a colourless solution of mercury(II) chloride (27 mg, 100 µmol) in dichloromethane (1 mL) was added dropwise over 10 minutes. The solution was left to stir overnight. The solution was concentrated and layered with hexane. After 24 hours, white needle-crystals of suitable quality for X-ray diffraction of **L2HgCl$_2$** (58 mg, 78%) were isolated and dried *in vacuo*. **^{1}H NMR:** δ_H (400.3 MHz, $CDCl_3$) 7.67 (1H, dd, $^3J_{HP}$ 11.4, $^3J_{HH}$ 7.4 Hz, H-8), 7.36 (1H, d, $^3J_{HH}$ 7.2 Hz, H-7), 7.02 (1H, d, $^3J_{HH}$ 7.5 Hz, H-3), 6.98 (2H, s, H-17), 6.87 (1H, d, $^3J_{HH}$ 7.5 Hz, H-2), 3.37–3.22 (4H, m, H-11,12), 3.12 (2H, dhept, $^2J_{HP}$ 10.5, $^3J_{HH}$ 6.9 Hz, H-13), 2.39 (6H, s, *o*-CH_3), 2.27 (3H, s, *p*-CH_3), 1.50 (6H, dd, $^3J_{HP}$ 19.1, $^3J_{HH}$ 6.9 Hz, 2 × CH_3, H-14), 1.39 (6H, dd, $^3J_{HP}$ 19.8, $^3J_{HH}$ 7.0 Hz, 2 × CH_3, H-14). **^{13}C DEPTQ NMR:** δ_C (100.7 MHz, $CDCl_3$) 154.2 (d, $^4J_{CP}$ 2.2 Hz, qC-6), 146.9 (s, qC-4), 144.0 (s, q-15), 143 (d, $^3J_{CP}$ 10.2 Hz, qC-5), 141.2 (s, qC-17), 136.6 (s, C-8), 133.8 (d, $^2J_{CP}$ 12.6 Hz, qC-10), 130.1 (s, C-17), 130.0 (s, C-2), 126.7 (d, $^4J_{CP}$ 9.3 Hz, C-16), 123.2 (s, qC-1), 122.3 (s, C-3), 119.9 (d, $^3J_{CP}$ 8.6 Hz, C-7), 117.4 (d, $^1J_{CP}$ 34.5 Hz, qC-8), 30.8 (s, C-11/12), 29.7 (s, C-11/12), 29.2 (d, $^1J_{CP}$ 20.8 Hz, C-13), 24.6 (s, *o*-CH_3), 21.4 (s, *p*-CH_3), 20.4 (d, $^2J_{CP}$ = 2.3 Hz, 2 × CH_3, C-14), 19.1 (d, $^2J_{CP}$ 3.4 Hz, 2 × CH_3, C-14). **^{31}P{^{1}H} NMR:** δ_P (162.0 MHz, $CDCl_3$) 55.8 (s, with ^{77}Se and ^{199}Hg satellites, $^1J_{PHg}$ 6264.3, $^2J_{PSe}$ 186.9 Hz). **^{31}P{^{1}H} CP MAS NMR**: δ_P (9.4 T, 14 kHz) 56.0 (s, with ^{77}Se and ^{199}Hg satellites, $^1J_{PHg}$ 6136.6, $^2J_{PSe}$ 205.0 Hz). **^{77}Se{^{1}H} NMR:** δ_{Se} (95.4 MHz, $CDCl_3$) 279.3 (d, $^2J_{SeP}$ 178.7 Hz, with ^{199}Hg satellites, $^1J_{SeHg}$ 909.3 Hz). **^{77}Se{^{1}H} CP MAS NMR:** δ_{Se} (9.4 T, 10 kHz) 262.8 (d, $^2J_{SeP}$ 205 Hz, $^1J_{SeHg}$ ≈1040 Hz). **IR** (KBr disk) ν_{max}/cm^{-1} 2913m ($\nu_{C–H}$), 2863w, 1603m, 1455m, 1407s, 1303s, 843m (νC–P). **MS** (ASAP) m/z (%) 269.1 (96) [M – $HgCl_2$ – SeMes], 469.1 (100) [M – $HgCl_2$ + H]. **HRMS** (ASAP) *m/z* (%) Calcd. for $C_{27}H_{34}PSe$: 469.1565, found: 469.1560 [M – $HgCl_2$ + H].

Synthesis of L3HgCl$_2$

To a suspension of **L3** (110 mg, 200 µmol) in ethanol (10 mL) a colourless solution of mercury(II) chloride (54 mg, 200 µmol) in ethanol (5 mL) was added dropwise over 10 minutes. The solution was left to stir overnight. The white precipitate was filtered and dried *in vacuo* to afford **L3HgCl$_2$** (120 mg, 73%). **^{1}H NMR:** δ_H (400.3 MHz, $CDCl_3$) 7.71 (1H, dd, $^3J_{HP}$ 11.2, $^3J_{HH}$ 7.5 Hz, H-8), 7.41 (1H, d, $^3J_{HH}$ 7.3 Hz, H-2), 7.17 (2H, s, H-18), 7.11 (1H, d, $^3J_{HH}$ 7.5 Hz, H-7), 6.98 (1H, d, $^3J_{HH}$ 7.5 Hz, H-3), 3.50 (2H, hept, $^3J_{HH}$ 6.8 Hz, *o*-C<u>H</u>(CH_3)$_2$), 3.42–3.30 (4H, m, H-11,12), 3.25–3.15 (2H, m, H-13) 2.96 (1H, hept, $^3J_{HH}$ 6.9 Hz, *p*-C<u>H</u>(CH_3)$_2$), 1.59 (6H, dd, $^3J_{HP}$ 18.8, $^3J_{HH}$ 6.9 Hz, 2 × CH_3, H-

14) 1.50 (6H, dd, $^3J_{HP}$ 19.8, $^3J_{HH}$ 7.1 Hz, 2 × CH_3, H-14), 1.30 (6H, d, $^3J_{HH}$ 6.9 Hz, *p*-CH(C$\underline{H_3}$)$_2$), 1.26–1.11 (12H, br s, *o*-CH(C$\underline{H_3}$)$_2$). **^{31}P{^{1}H} NMR:** δ_P (162.0 MHz, $CDCl_3$) 55.6 (s, with ^{77}Se and ^{199}Hg, $^1J_{PHg}$ 6160.1, $^2J_{PSe}$ 204.3 Hz). **^{77}Se{^{1}H} NMR:** δ_{Se} (57.3 MHz, $CDCl_3$) 246.6 (d, $^2J_{SeP}$ 204.4 Hz).

Synthesis of L4HgCl$_2$

Experimental procedure is identical to **L2HgCl$_2$** but using the following quantities **L4** (120 mg, 200 µmol), $HgCl_2$ (70 mg, 260 µmol), and CH_2Cl_2 (15 mL). The volatiles were removed *in vacuo* to yield **L4HgCl$_2$** as a white powder. The crude material was purified by a slow diffusion of hexane into a saturated CH_2Cl_2 solution of **L4HgCl$_2$**. This yielded 0.050 g of crystalline material. Selected crystals were used for single crystal X-ray diffraction, which revealed these to be **L4HgCl$_2$** cocrystallised with $HgCl_2$ (overall formula **[L4Hg$_2$Cl$_4$][L4Hg$_3$Cl$_6$]**). **Elemental Analysis:** Calcd. (%) for $C_{36}H_{51}PSeHgCl_2\cdot(HgCl_2)_{0.3}$: C 45.67, H 5.43; Found: C 45.51, H 5.15 (indicates some co-crystallisation with $HgCl_2$ is taking place). **^{1}H NMR δ_H** (499.9 MHz, $CDCl_3$) 7.68 (1H, dd, $^3J_{HP}$ 11.8, $^3J_{HH}$ 7.4 Hz, H-8), 7.54 (2H, s, H-17), 7.37 (1H, d, $^3J_{HH}$ 7.3 Hz, H-7), 6.97 (1H, $^3J_{HH}$ 7.6 Hz, H-3), 6.21 (1H, d, $^3J_{HH}$ 7.6 Hz, H-2), 3.42−3.21 (6H, m, H-11,12,13), 1.63−1.53 (12H, m, 4 × CH_3, H-14), 1.48 (18H, s, *o*-C(CH_3)$_3$), 1.37 (9H, s, *p*-C(CH_3)$_3$). **^{13}C DEPTQ NMR**: δ_C (125.7 MHz, $CDCl_3$) 155.5 (s, qC-4), 152.7 (s, qC-6), 136.0 (s, C-8), 131.9 (s, C-2), 124.4 (s, C-17), 122.1 (s, C-3), 119.3 (s, C-7), 39.2 (s, *o*-$\underline{C}$(CH_3)$_3$), 35.5 (s, *p*-$\underline{C}$(CH_3)$_3$), 32.8 (s, *o*-C($\underline{C}H_3$)$_3$), 31.4 (s, *p*-C($\underline{C}H_3$)$_3$), 30.7 (s, C-11/12), 29.5 (d, $^1J_{CP}$ 21.8 Hz, C-13), 29.5 (s, C-11/12), 20.8 (s, 2 × CH_3, H-14), 18.2 (s, 2 × CH_3, H-14). **^{31}P{^{1}H} NMR:** δ_P (162.0 MHz, $CDCl_3$) 59.3 (s, with ^{77}Se and ^{199}Hg satellites $^1J_{PHg}$ 6276.4, $^2J_{PSe}$ 210.8 Hz]. **^{77}Se{^{1}H} NMR** δ_{Se} (95.4 MHz, $CDCl_3$) 325.5 (d, $^2J_{SeP}$ 209.9 Hz). **MS (EI)** *m/z* (%) 831.2 (100, M–Cl). **HRMS (ESI)** *m/z* (%) Calcd. for C36H51PSeHgCl: 831.2288; found: 831.2273 [M−Cl].

Synthesis of L1BH$_3$

To a colourless solution of **L1** (300 mg, 705 µmol) in chloroform (7 mL), a solution of borane dimethylsulfide (0.08 mL, 846 µmol) in chloroform (1 mL) was added dropwise. The mixture was stirred overnight at ambient temperature and then filtered to collect the precipitate which was subsequently washed with ethanol (2 × 8 mL) to afford **L1BH$_3$** as a white powder (214 mg, 73%). M.p. 165 °C (decomp.). Crystals for X-ray diffraction were grown by slow evaporation of a saturated solution of **L1BH$_3$** in CH_2Cl_2 at ambient conditions. **Elemental Analysis:** Calcd. (%) for $C_{24}H_{30}PSeB$ (439.24): C 65.63, H 6.88; Found: C 65.52, H 6.77. **^{1}H NMR:** δ_H (500.1 MHz, $CDCl_3$) 8.57 (1H, br s, H-8), 8.04 (1H, d, $^3J_{HH}$ 7.3 Hz, H-2), 7.37 (1H, d, $^3J_{HH}$ 7.3 Hz, H-7), 7.28 (1H, d, $^3J_{HH}$ 7.3 Hz), 7.17–7.10 (3H, m, H-16,18), 7.02–6.95 (2H, m, H-17), 3.47 (2H, br s, H-13), 3.43 (4H, s, H-11,12), 1.37 (6H, dd, $^3J_{HP}$ 15.0, $^3J_{HH}$ 7.0 Hz, 2 × CH_3, H-14), 1.16–0.34 (9H, br m, 2 × CH_3 (H-14), 1 × BH_3). **^{11}B{^{1}H} NMR:** δ_B (96.3 MHz, $CDCl_3$) –41.7 (br d, $^1J_{BP}$ ≈ 35 Hz). **^{13}C DEPTQ NMR:** δ_C (125.7 MHz, $CDCl_3$) 152.1 (s, qC-6), 150.1 (s, qC-4), 143.5 (s, C-2), 141.2 (d, $^3J_{CP}$ 6.4 Hz, qC-5), 135.8 (br m, qC-2), 129.4 (s, C-16), 129.3 (s, C-17), 126.3 (s, C-18), 121.1 (s, C-3), 120.6 (s, qC-9), 120.2 (s, qC-1), 119.4 (d, $^3J_{CP}$ 13.8 Hz, C-8), 30.0 (s, C-11,12), 25.2 (d, $^1J_{CP}$ 33.2 Hz, C-13), 19.0 (s, 2 × CH_3, C-14), 18.8 (br s, 2 × CH^3, C-14). **^{31}P{^{1}H} NMR:** δ_P (202.4 MHz, $CDCl_3$) 46.5 (br s). **^{77}Se{^{1}H} NMR**: δ_{Se} (76.4 MHz, $CDCl_3$) 412.6 (br s). **HRMS (APCI):** m/z (%) Calcd. for $C_{24}H_{30}PSeB$: 439.1271, found: 439.1267 (100) [M–H], Calcd. for $C_{24}H_{29}PSe$: 427.1094, found: 427.1092 (95) [M–BH_2].

Further NMR Spectra and Spin Simulations

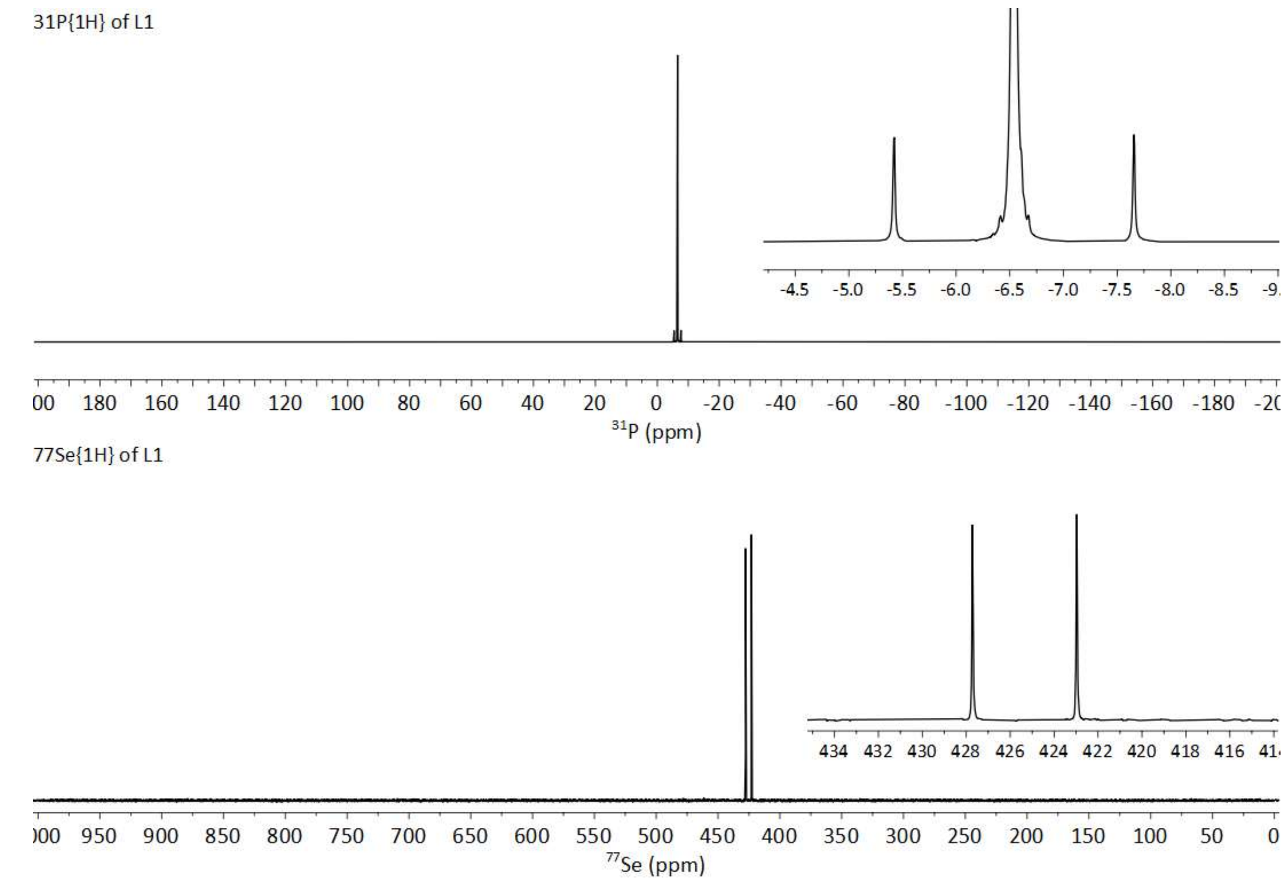

*Figure S3: The $^{31}P\{^1H\}$ (top) and $^{77}Se\{^1H\}$ (bottom) NMR spectra of **L1** recorded at 202.4 and 95.4 MHz, respectively, in $CDCl_3$.*

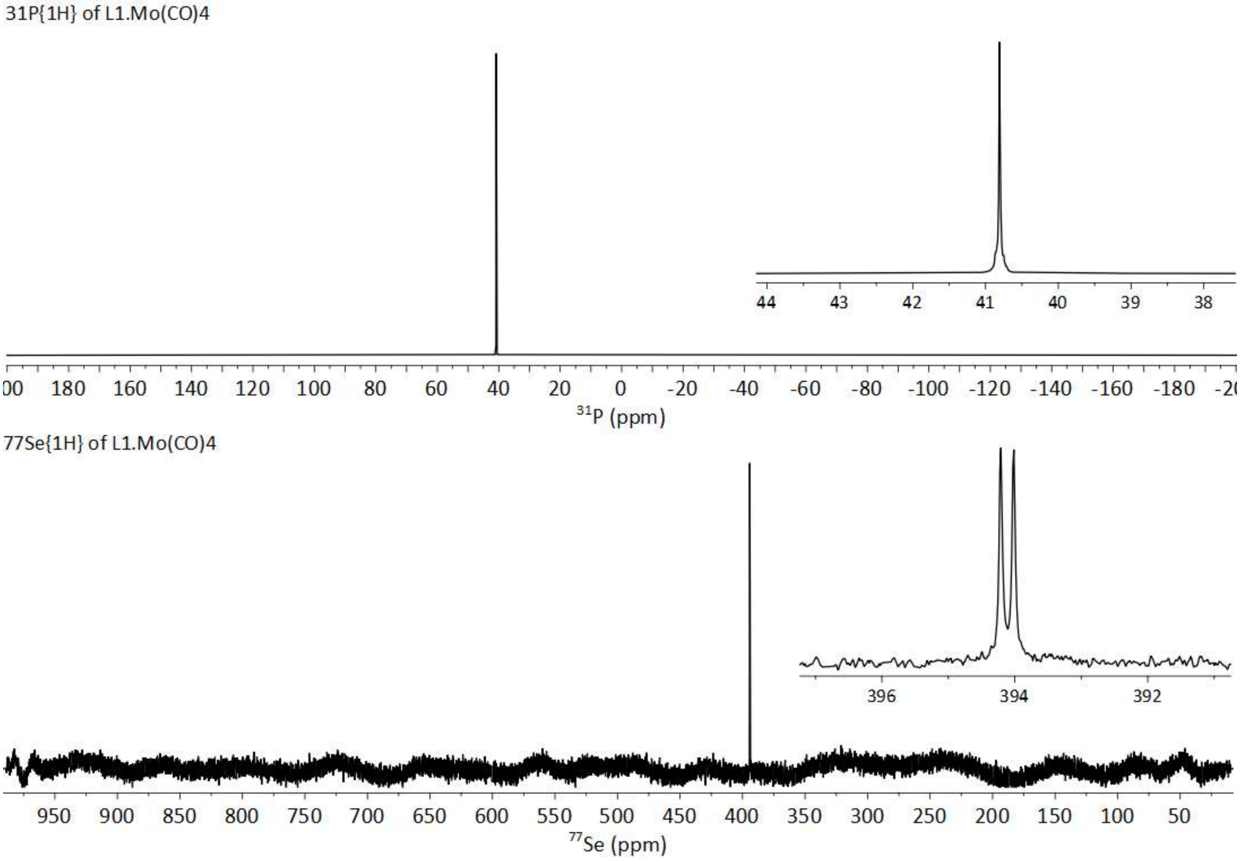

*Figure S4: The $^{31}P\{^{1}H\}$ (top) and $^{77}Se\{^{1}H\}$ (bottom) NMR spectra of **L1Mo(CO)$_4$** with expansions showing J_{SeP} is only observable in the $^{77}Se\{^{1}H\}$ spectrum. Recorded at 121.5 and 76.3 MHz, respectively, in $CDCl_3$.*

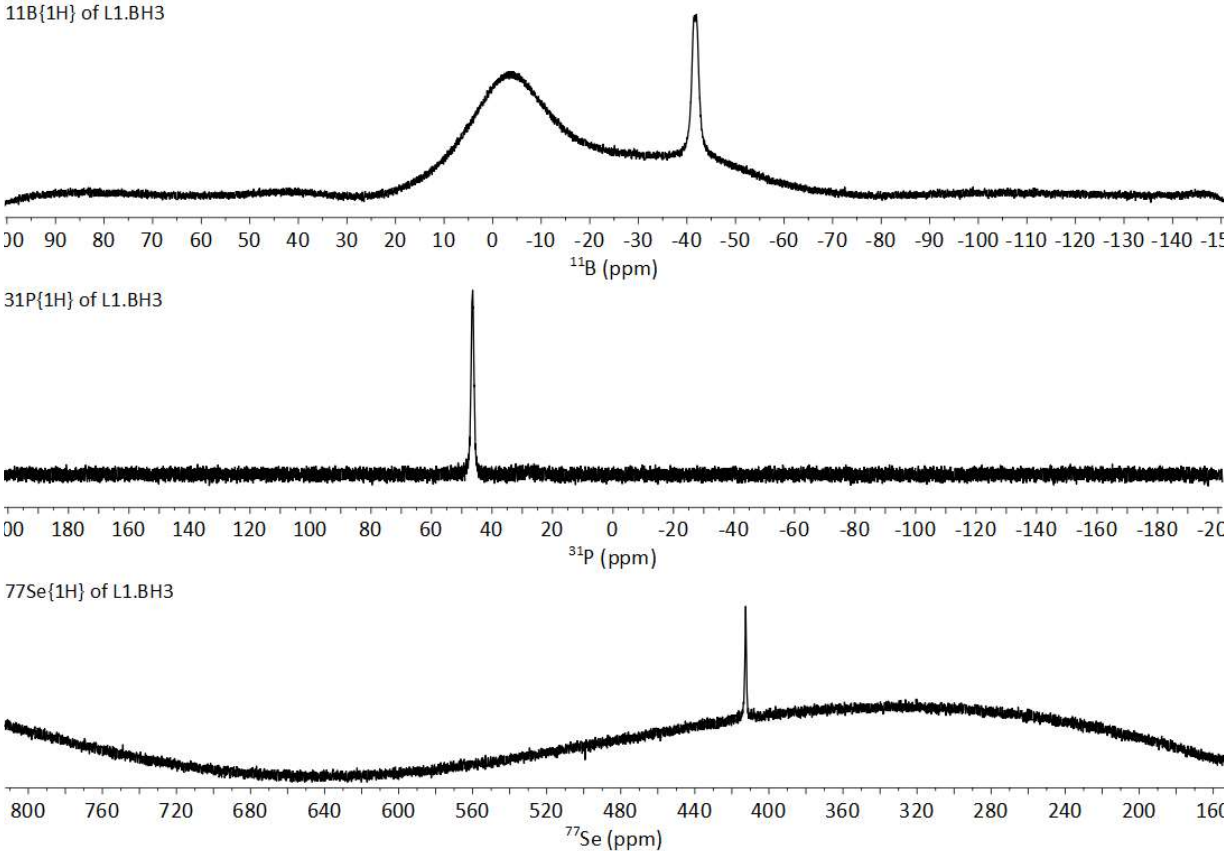

*Figure S5: The $^{11}B\{^{1}H\}$ (top), $^{31}P\{^{1}H\}$ (centre) and $^{77}Se\{^{1}H\}$ (bottom) NMR spectra of **L·BH₃** recorded at 96.3, 202.4 and 76.4 MHz, respectively, in $CDCl_3$.*

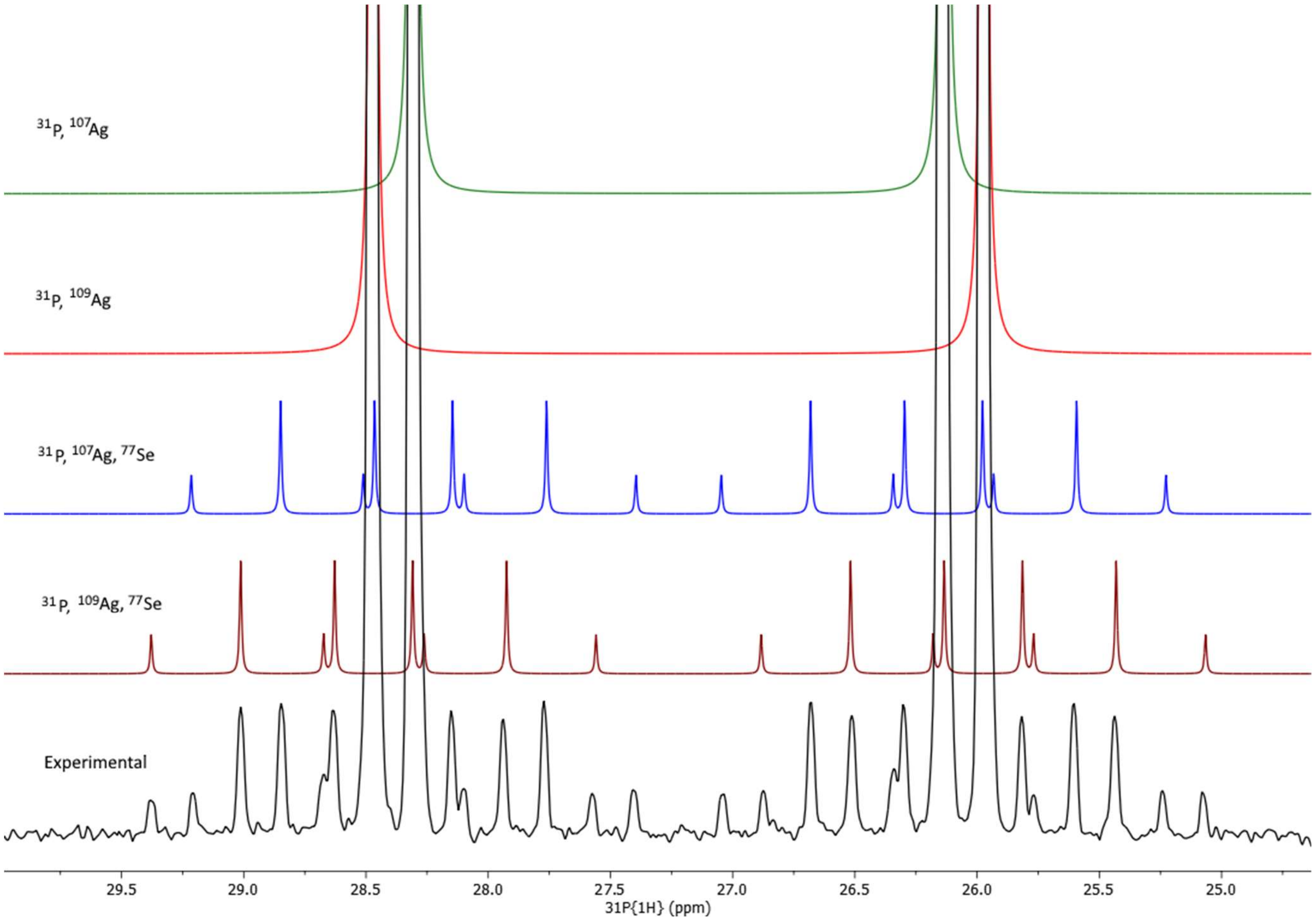

Figure S6: Spin system simulation (top four) and experimental (bottom) $^{31}P\{^{1}H\}$ NMR spectra (202.5 MHz) of $[L2_2Ag][Al(OC(CF_3)_3)_4]$. Four independent simulated spin systems (isotopomers) are shown, these combine to form the overall (simulated) spectrum. Relevant parameters (δ_P and J) extracted from the simulations are shown in Table 2 in the main text.

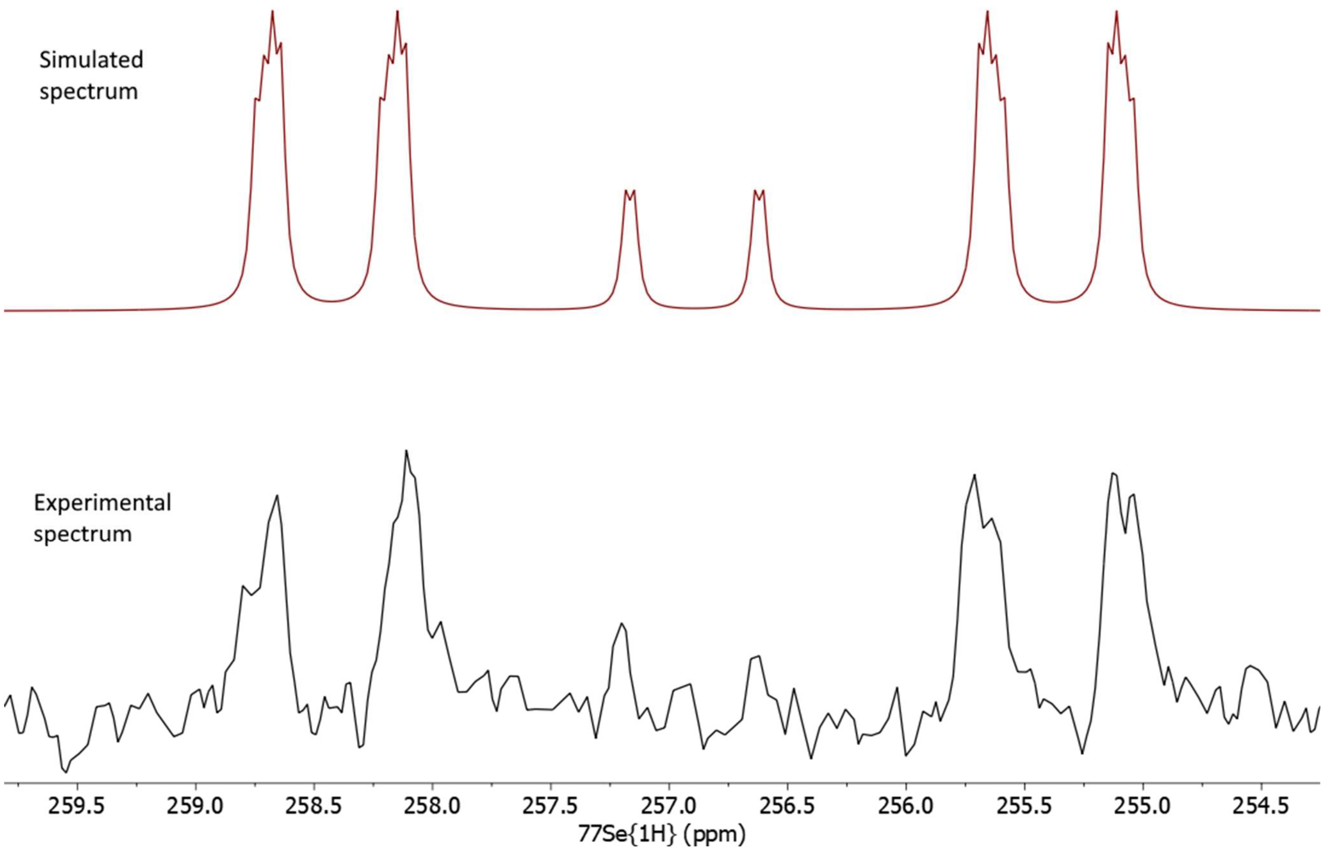

Figure S7: Simulated(top) and measured (bottom) $^{77}Se\{^{1}H\}$ NMR spectra (76.4 MHz) of the $[L2_2Ag][Al(OC(CF_3)_3)_4]$ complex. Low signal to noise ratio of the experimental spectrum results in the simulated J couplings being only approximate.

X-ray Diffraction

X-ray diffraction data for **2Mes** were collected using a Rigaku MM-007HF High Brilliance RA generator/confocal optics with XtaLAB P200 diffractometer [Cu Kα radiation (λ = 1.54187 Å)]. Diffraction data for **2Tripp**, **nBuSeMes***, **L1**, **$L1BH_3$**, **$L1Mo(CO)_4$**, **$[(L1)_2Ag][SbF_6]$**, **$L1HgCl_2$**, **$[L1HgCl_2]_2$**, **L2**, **$L2PdCl_2$**, **$[(L2)_2Ag][Al(OC(CF_3)_3)_4]$**, **$L2PtCl_2$**, **L3**, **L4**, **$[L4Hg_2Cl_4][L4Hg_3Cl_6]$**, and **$L4PdCl_2$** were collected using a Rigaku FR-X Ultrahigh Brilliance Microfocus RA generator/confocal optics with XtaLAB P200 diffractometer [Mo Kα radiation (λ = 0.71073 Å)]. Data for **$L1PtCl_2$**, **$L2Mo(CO)_4$**, **$L2HgCl_2$** were collected using a Rigaku SCXmini CCD system with a SHINE monochromator [Mo Kα radiation (λ = 0.71073 Å)]. Intensity data for all crystals were collected using ω steps accumulating area detector images spanning at least a hemisphere of reciprocal space. Data for all compounds analysed were collected using CrystalClear[7] and processed (including correction for Lorentz, polarization, and absorption) using CrystalClear[7] or CrysAlisPro.[8] Structures were solved by direct (SHELXS,[9] SIR2011[10]), Patterson (PATTY[11]), charge-flipping (Superflip[12]), or dual-space (SHELXT[13]) methods, and refined by full-matrix least-squares against F^2 (SHELXL[14]). Non-hydrogen atoms were refined anisotropically, and hydrogen atoms were refined using a riding model. Crystals of **$L1BH_3$**, **$[(L2)_2Ag]$ $[Al(OC(CF_3)_3)_4]$**, and **$[L4Hg_2Cl_4][L4Hg_3Cl_6]$** were affected by non-merohedric twinning. The structure of **$[L4Hg_2Cl_4][L4Hg_3Cl_6]$** showed high proportions of void space (1426 $Å^3$) and the SQUEEZE[15] routine implemented in PLATON[16] was used to remove the contribution to the diffraction pattern of the unordered electron density in the void spaces. All calculations except SQUEEZE were performed using either the CrystalStructure[17] or the Olex2[18] interface. Despite the structures **2Tripp**, **nBuSeMes***, **L1PtCl2** and **$[L4Hg_2Cl_4][L4Hg_3Cl_6]$** being of less than ideal quality, the structure solutions were clear and unambiguous. Selected crystallographic data are presented in Tables S1-S2 and Figures S8-S10. CCDC 2278198-2278217 contain the supplementary crystallographic data for this paper. The data can be obtained free of charge from The Cambridge Crystallographic Data Centre via www.ccdc.cam.ac.uk/structures.

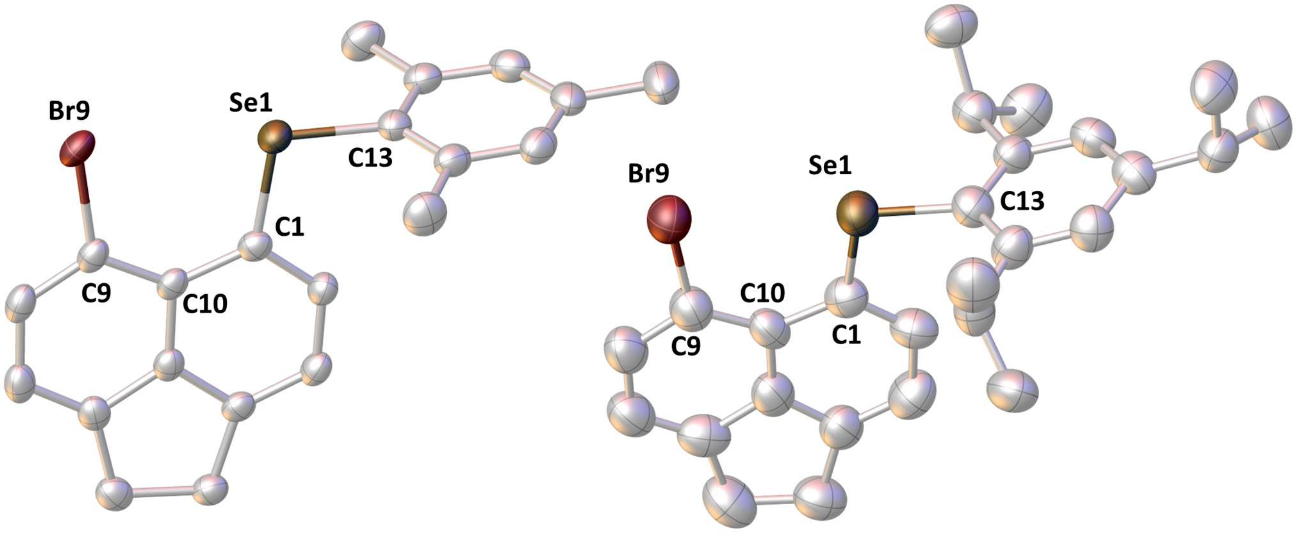

Figure S8: Molecular structures of **2Mes** and **2Tripp**. Hydrogen atoms are omitted for clarity. Anisotropic displacement ellipsoids are plotted at the 50% probability level.

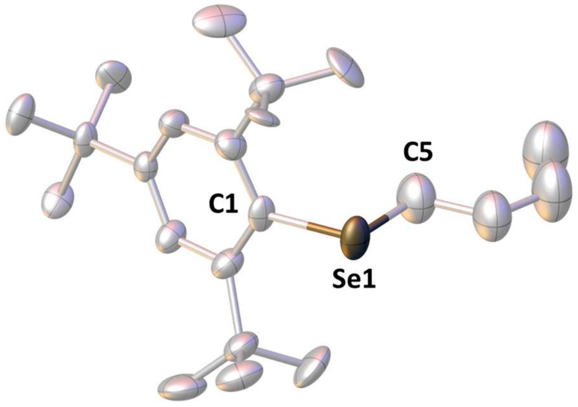

Figure S9: Molecular structure of **nBuSeMes***. Hydrogen atoms are omitted for clarity. Anisotropic displacement ellipsoids are plotted at the 50% probability level.

Table S1 Selected crystallographic data.

	**2Mes**	**2Tripp**	**nBuSeMes***
empirical formula	$C_{21}H_{19}BrSe$	$C_{27}H_{31}BrSe$	$C_{22}H_{38}Se$
fw	430.25	514.41	381.48
crystal description	colourless plate	colourless plate	yellow plate
crystal size [mm^3]	0.20×0.10×0.01	0.07×0.05×0.01	0.18×0.04×0.01
temperature [K]	125	173	173
space group	$P\bar{1}$	$P2_1/c$	*Pnma*
a [Å]	8.7753(3)	17.438(2)	9.2990(5)
b [Å]	14.0389(5)	8.7847(16)	11.4117(8)
c [Å]	15.4921(6)	15.116(2)	20.4210(12)
α [°]	65.895(3)		
β [°]	86.109(3)	95.659(13)	
γ [°]	88.350(3)		
vol [Å]^3	1738.11(12)	2304.3(6)	2167.0(2)
Z	4	4	4
ρ (calc) [g/cm^3]	1.644	1.483	1.169
μ [mm^{-1}]	5.569	3.380	1.733
F(000)	856	1048	816
reflections collected	17964	29591	27084
independent reflections (R_{int})	6853 (0.0288)	5381 (0.1688)	2775 (0.1249)
parameters, restraints	421, 0	268, 0	213, 206
GOF on F^2	1.100	0.950	1.072
R_1 [$I > 2\sigma(I)$]	0.0437	0.0839	0.0886
wR_2 (all data)	0.1246	0.2028	0.2155
largest diff. peak/hole [e/Å^3]	0.96, −1.16	0.78, −0.39	0.44, −0.46

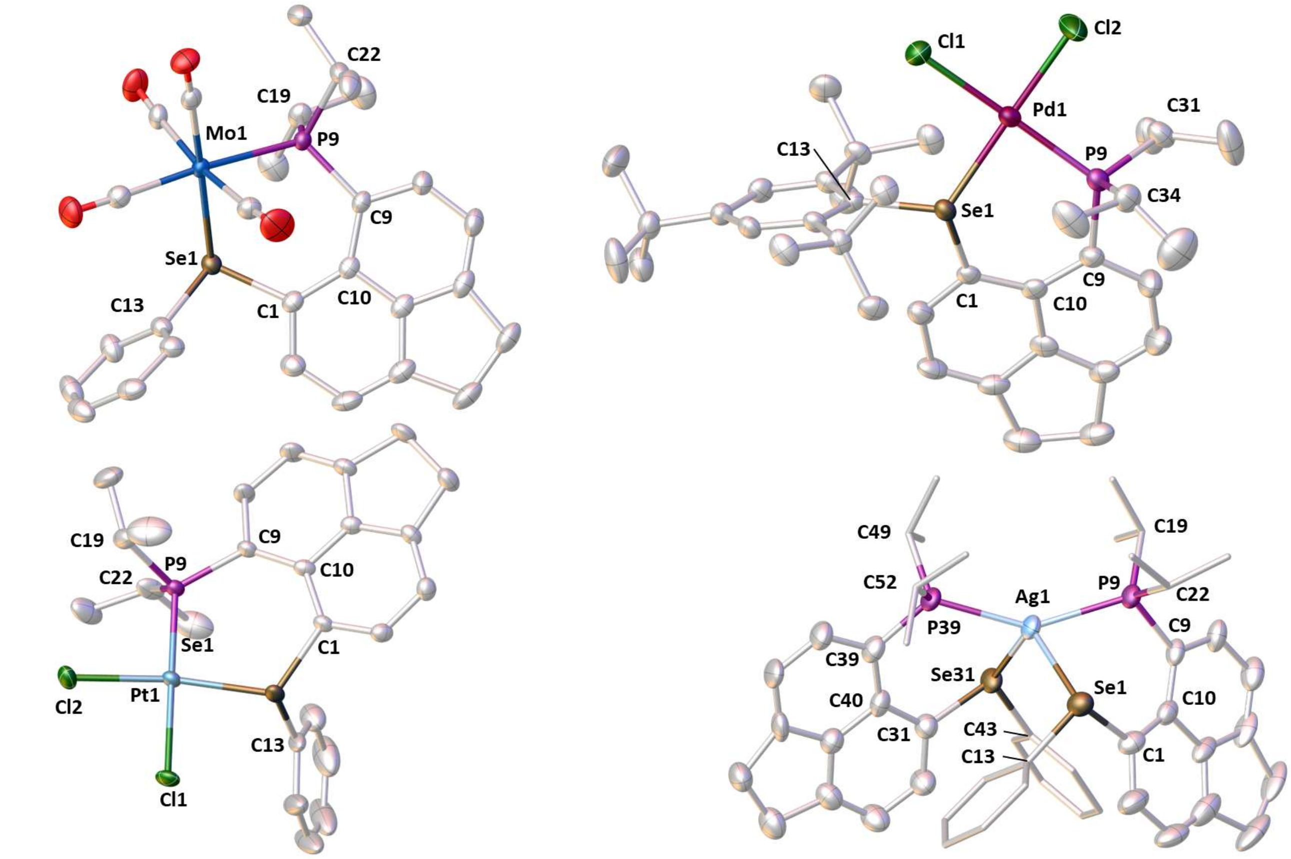

Figure S10 From left to right, top to bottom, molecular structure of **L1Mo(CO)$_4$**, **L4PdCl$_2$**, **L1PtCl$_2$**, and **[(L1)$_2$Ag]SbF$_6$**. Hydrogen atoms, solvating molecules and counter ions are omitted for clarity. Anisotropic displacement ellipsoids are plotted at the 50% probability level.

Table S 2 Selected crystallographic data.

	**L1**	**L2**	**L3**	**L4**
empirical formula	$C_{24}H_{27}PSe$	$C_{27}H_{33}PSe$	$C_{33}H_{45}PSe$	$C_{36}H_{51}PSe$
fw	425.41	467.49	551.65	593.73
crystal description	colourless needle	yellow prism	colourless prism	colourless prism
crystal size [mm^3]	0.25×0.01×0.01	0.27×0.15×0.13	0.30×0.12×0.05	0.12×0.05×0.05
temperature [K]	173	173	173	93
space group	$P2_1/c$	$P\bar{1}$	$P2_1/n$	$C2/c$
a [Å]	5.8906(12)	7.9496(8)	10.6778(4)	42.213(5)
b [Å]	42.014(11)	10.4269(8)	26.5223(7)	8.3981(11)
c [Å]	8.168(2)	14.5488(16)	11.7623(4)	18.361(2)
α [°]		86.379(7)		
β [°]	91.698(7)	89.562(7)	116.714(4)	90.172(4)
γ [°]		74.078(5)		
vol [Å]^3	2020.6(8)	1157.3(2)	2975.5(2)	6509.1(14)
Z	4	2	4	8
ρ (calc) [g/cm^3]	1.398	1.341	1.231	1.212
μ [mm^{-1}]	1.943	1.703	1.335	1.225
F(000)	880	488	1168	2528
reflections collected	23512	14342	38258	45404
independent reflections (R_{int})	3672 (0.0876)	4209 (0.0218)	6949 (0.0280)	5906 (0.0313)
parameters, restraints	239, 0	269, 0	326, 0	344, 0
GoF on F^2	1.064	1.091	1.059	1.071
R_1 [$I > 2\sigma(I)$]	0.0414	0.0222	0.0273	0.0197
wR_2 (all data)	0.0868	0.0611	0.0713	0.0541
largest diff. peak/hole [e/Å^3]	0.35/-0.39	0.48/-0.19	0.37/-0.29	0.38, -0.19

	L1BH$_3$	L1Mo(CO)$_4$	L2Mo(CO)$_4$
empirical formula	$C_{24}H_{30}BPSe$	$C_{28}H_{27}MoO_4PSe$	$C_{31}H_{33}MoO_4PSe$
fw	439.24	633.39	675.47
crystal description	colourless chip	yellow prism	yellow prism
crystal size [mm^3]	0.12×0.07×0.03	0.22×0.12×0.10	0.21×0.11×0.10
temperature [K]	173	173	173
space group	$P2_1/n$	$P2_1/n$	$P2_1/c$
a [Å]	9.481(3)	8.1516(10)	10.2081(7)
b [Å]	12.189(4)	17.8959(18)	18.8288(13)
c [Å]	18.814(5)	18.5744(18)	15.5962(10)
α [°]			
β [°]	97.706(7)	101.536(4)	105.708(6)
γ [°]			
vol [Å]^3	2154.6(11)	2654.9(5)	2885.7(3)
Z	4	4	4
ρ (calc) [g/cm^3]	1.354	1.585	1.555
μ [mm^{-1}]	1.824	1.956	1.805
F(000)	912	1272	1368
reflections collected	23708	32310	24938
independent reflections (R_{int})	3945 (0.0665)	4871 (0.0221)	5281 (0.0740)
parameters, restraints	249, 0	320, 0	350, 0
GoF on F^2	1.195	1.032	0.940
R_1 [$I > 2\sigma(I)$]	0.0662	0.0165	0.0251
wR_2 (all data)	0.1638	0.0425	0.0635
largest diff. peak/hole [e/Å^3]	1.34/-1.45	0.39/-0.28	0.37/-0.42

	L1PtCl$_2$	L2PtCl$_2$	L2PdCl$_2$	L4PdCl$_2$
empirical formula	$C_{24}H_{27}Cl_2PPtSe$	$C_{27}H_{33}Cl_2PPtSe$	$C_{27}H_{33}Cl_2PPdSe$	$C_{36}H_{51}Cl_2PPdSe$
fw	691.41	733.45	644.76	770.99
crystal description	colourless prism	colourless prism	orange prism	orange prism
crystal size [mm^3]	0.51×0.42×0.33	0.09×0.06×0.04	0.09×0.06×0.03	0.06×0.06×0.04
temperature [K]	173	173	93	173
space group	$P2_12_12_1$	$P2_1/n$	$P2_1/n$	$P2_12_12_1$
a [Å]	11.9588(8)	11.2002(2)	11.184(2)	11.8593(3)
b [Å]	13.3191(9)	16.5017(3)	16.370(3)	13.5418(2)
c [Å]	14.9508(10)	14.4032(2)	14.375(3)	21.2990(5)
α [°]				
β [°]		103.844(2)	103.694(4)	
γ [°]				
vol [Å]^3	2381.4(3)	2584.70(8)	2557.0(8)	3420.54(13)
Z	4	4	4	4
ρ (calc) [g/cm^3]	1.928	1.885	1.675	1.497
μ [mm^{-1}]	7.698	7.120	2.435	1.834
F(000)	1328	1424	1296	1584
reflections collected	20502	33023	28639	47940
independent reflections (R_{int})	4304 (0.1032)	5972 (0.0362)	4671 (0.0569)	7801 (0.0815)
parameters, restraints	262, 0	296, 0	296, 0	383, 0
GoF on F^2	0.849	1.149	1.092	1.020
R_1 [$I > 2\sigma(I)$]	0.0184	0.0383	0.0423	0.0374
wR_2 (all data)	0.0433	0.1012	0.1148	0.0783
largest diff. peak/hole [e/Å^3]	0.64/-0.96	3.475/-1.509	1.576/-0.479	1.06, -0.78

	**L1HgCl$_2$**	**[L1HgCl$_2$]$_2$·** 2CHCl$_3$	**L2HgCl$_2$**	**[L4Hg$_2$Cl$_4$][L4Hg$_3$Cl$_6$]**
empirical formula	$C_{24}H_{27}Cl_2HgPSe$	$C_{50}H_{56}Cl_{10}Hg_2P_2Se_2$	$C_{27}H_{33}Cl_2HgPSe$	$C_{72}H_{102}Cl_{10}Hg_5P_2Se_2$
fw	696.91	1632.57	738.99	2544.95
crystal description	colourless prism	colourless prism	colourless prism	colourless prism
crystal size [mm^3]	0.18×0.15×0.15	0.30×0.15×0.15	0.17×0.10×0.05	0.13×0.02×0.02
temperature [K]	93	173	173	173
space group	$P2_1/n$	$P\bar{1}$	$P2_1/c$	$P2_1/c$
a [Å]	8.96592(14)	9.50333(9)	11.6597(8)	11.4428(4)
b [Å]	12.02550(19)	10.59340(10)	12.1924(8)	28.5226(10)
c [Å]	21.9894(3)	14.80860(15)	18.8824(13)	28.3929(11)
α [°]		90.4971(8)		
β [°]	95.0011(14)	103.1590(9)	95.114(5)	90.454(3)
γ [°]		100.4210(8)		
vol [Å]^3	2361.86(6)	1425.79(2)	2673.6(3)	9266.5(6)
Z	4	1	4	4
ρ (calc) [g/cm^3]	1.960	1.901	1.836	1.824
μ [mm^{-1}]	8.378	7.226	7.407	9.419
F(000)	1336	784	1432	4808
reflections collected	65387	48493	22971	120774
independent reflections (R_{int})	5461 (0.0360)	6336 (0.0347)	4904 (0.0932)	21844 (0.1184)
parameters, restraints	262, 0	298, 0	353, 190	901, 296
GoF on F^2	1.115	1.045	0.962	1.026
R_1 [$I > 2\sigma(I)$]	0.0159	0.0249	0.0275	0.1085
wR_2 (all data)	0.0402	0.0657	0.0711	0.3326
largest diff. peak/hole [e/Å^3]	0.68, -1.13	1.88, -2.03	1.45, -1.10	3.27, -0.78

	**$[(L1)_2Ag]SbF_6$ $\cdot 0.5CH_2Cl_2$**	**$[(L2)_2Ag]$ $[Al(OC(CF_3)_3)_4]$**
empirical formula	$C_{48.5}H_{55}AgClF_6P_2SbSe_2$	$C_{70}H_{66}AgAlF_{36}O_4P_2Se_2$
fw	1236.90	2009.95
crystal description	colourless prism	colourless prism
crystal size [mm^3]	0.11×0.08×0.03	0.15×0.15×0.09
temperature [K]	173	173
space group	$P\bar{1}$	$P\bar{1}$
a [Å]	10.4230(19)	14.7108(3)
b [Å]	14.000(3)	15.3368(3)
c [Å]	16.718(3)	18.7187(3)
α [°]	85.018(9)	73.4404(17)
β [°]	79.500(9)	75.4014(15)
γ [°]	87.871(9)	84.5536(15)
vol [Å]^3	2389.0(8)	3916.18(13)
Z	2	2
ρ (calc) [g/cm^3]	1.719	1.704
μ [mm^{-1}]	2.667	1.371
F(000)	1226	2000
reflections collected	40469	120064
independent reflections (R_{int})	8442 (0.0775)	17101 (0.0673)
parameters, restraints	557, 0	1060, 0
GoF on F^2	1.147	1.070
R_1 [$I > 2\sigma(I)$]	0.0658	0.0739
wR_2 (all data)	0.1793	0.2417
largest diff. peak/hole [e/Å^3]	1.04, -1.06	3.53, -1.64

Computational Details

Structures were fully optimised at the PBE0-D3 level of density functional theory[19, 20] including Grimme's three-body dispersion correction[21-23] with Becke-Johnson damping,[24, 25] using a quasi-relativistic effective core potential along with its (6s5p3d) valence basis on Hg,[26] Binning and Curtiss' 962(d) basis on Se,[27] and 6-31G(d) basis elsewhere, together with a fine integration grid (75 radial shells with 302 angular points per shell). Comparable levels have been shown to perform very well for structural parameters of metal complexes from the third transition row;[28] (see, however, below for a discussion of the Hg–P distance). The minimum nature of the stationary points has been verified by evaluation of the harmonic vibrational frequencies, which were all real. These computations have been performed using the Gaussian 09 series of programs.[29]

Using these optimised structures (coordinates are given below), unrestricted four-component calculations were carried out within the Dirac–Kohn–Sham (DKS) framework, as implemented in the ReSpect program with restricted kinetic balanced basis sets and noncollinear spin density.[30-31] The BP86 exchange-correlation functional was employed,[32, 33] together with Dyall's Gaussian-type orbital (GTO) valence double zeta basis sets on Hg and Se,[34, 35] and uncontracted IGLO-II basis for all other atoms.[36] Coupling pathways were visualised using the coupling deformation density (CDD).[37] For visualization a local version of the deMon-NMR program[38-39] as well as GaussView[40] were employed. The computed *J* couplings are collected in Table S3.

Table S3: Computed J-couplings in **L1** and **L1HgCl₂** (in Hz, BP86 level on PBE0-D3 optimised structures).

compound<br>coupling	**L1**<br>J_{SeP}	**L1HgCl₂**<br>J_{SeP}	 J_{SeHg}	 J_{HgP}
calc. (without SO)[a]	473.2	23.0	−1040.6	2236.5
calc. (with SO)	473.6	17.6	−983.0	2181.2
expt.	*452*	*86.7*	*(±)695*	*6610*

[a]Calculated without taking the spin-orbit coupling operator in the ground state Hamiltonian into account.

In order to probe if the rather large deviation between the computed and observed J_{HgP} coupling in **L1HgCl₂** is related to the noticeable overestimation of the Hg–P bond distance in the optimised structure (calc. 2.525 Å, X-ray 2.408 Å, see Table 3 in the main paper), we performed an NMR calculation using the X-ray derived coordinates (after relaxation of the H positions at the PBE0-D3 level). For this structure, J_{HgP} = 3627.8 Hz is obtained (including SO), in slightly better agreement with experiment, but still too low by *ca.* 45%. A more thorough study of effects of basis set and exchange-correlation functional is warranted for this property, but this was deemed outside the scope of this study.

Optimised coordinates (PBE0-D3/SDD/6-31G* level)

```
53
L1
 Se  0.0251738911 5.8122120595 1.9194126611
 P  1.566019132 3.1975721666 1.804321419
 C  3.2968190348 5.9341546259 -0.8170059538
 C  4.4655098306 5.2744766965 -1.2792255179
```

C 2.7312675633 4.0750628506 0.6582931213

C 2.0271777159 7.9554487072 -1.1349599812

C 4.7749806153 4.0282471577 -0.7682275607

C -0.3408582688 7.6367543108 2.5126532382

C 3.1309437321 7.1878126395 -1.4593907311

C 0.6948216777 8.47914699 2.9588413692

C 3.7299578579 2.8991555194 3.7235672922

C 4.2688845322 7.417110993 -2.434793902

C -1.9756407545 9.3283601262 3.129490927

C 0.3914973929 9.74618792 3.4707505051

C 2.4054019596 5.3934741026 0.1640521179

C -1.6766823122 8.0637120892 2.5971484377

C 1.9505314764 1.3732528344 1.4860821769

C 5.163472208 6.1395757283 -2.3106468484

C 1.3001832197 6.250346355 0.5101609951

C 1.1720102759 0.5066384722 2.4889651379

C 1.5410905542 1.0272676901 0.046078887

C 1.1253452886 7.4686380567 -0.1553406727

C 2.3901870275 3.5961278656 3.4733274784

C -0.9442834703 10.1722434927 3.5624034696

C 1.3990825461 3.4054129095 4.6309524416

C 3.8951256843 3.4533213985 0.1866512981

H 1.7332950534 8.1398568615 2.8935882641

H 1.200653008 10.4005892675 3.8121414358

H -1.1778613775 11.1607388294 3.9706046922

H -3.0184087127 9.6568689222 3.1925720456

H -2.4790839565 7.4103338566 2.2395738063

H 2.5729677152 4.6827215602 3.3710034504

H 1.8393907354 3.7744127637 5.5754999632

H 0.466542407 3.9650601401 4.4473450636

H 1.136711692 2.3445879667 4.781682145

H 4.1910656873 3.2787478732 4.6542780283

H 3.6084603854 1.8078399304 3.8426157741

H 4.4412374446 3.0794629059 2.8998522426

H 3.0302498655 1.1703166377 1.6202890475

H 1.5259319899 0.6448484488 3.5232552721

H 0.0932068523 0.7424298479 2.4647811898

H 1.2889190621 -0.5628854259 2.2350987948

H 1.7150566064 -0.0459377558 -0.1541352075

H 0.466903991 1.2304139696 -0.1115850157

H 2.1062956541 1.611454916 -0.6981854016

H 4.1434198864 2.4534928282 0.5540978066

H 5.6648922481 3.475967583 -1.0889502474

H 0.2630904216 8.0878060811 0.1062121089

H 1.8330684148 8.9229111655 -1.6102109071

H 4.8338462878 8.3344451974 -2.1892624973

H 3.8943306122 7.55117286 -3.4656105143

H 6.1909836678 6.3915180922 -1.9919908978

H 5.2595393881 5.6135525895 -3.2774108476

56

L1HgCl2

Hg 5.3656218854 5.8845604306 7.9182054149

Se 3.9753630285 4.2573652356 9.8422329456

Cl 3.7519674014 6.0174283074 6.0905262537

Cl 5.9422479044 7.8071432747 9.3176808889

P 7.1248708371 4.0738354479 7.962708206

C 5.347940244 3.6241720957 11.0239095865

C 4.9602186775 3.6720895952 12.3552929476

H 4.0033908804 4.129623311 12.5894907763

C 5.7129618575 3.1152445407 13.4082781069

H 5.3587352275 3.2010396054 14.4313238547

C 6.8417941729 2.4114524065 13.080770507

C 7.2469722556 2.3507112348 11.7282704808

C 8.387055775 1.5306142608 11.5903154675

C 8.9282950895 1.3476011911 10.343671587

H 9.7870710275 0.7047561308 10.1728392842

C 8.3720620732 2.0772050399 9.2768315092

H 8.8706518276 2.0008589299 8.3136835586

C 7.2711723029 2.9228544624 9.3880611007

C 6.5929539917 3.0138756077 10.651808843

C 7.8037241296 1.629988799 13.9377654452

H 7.2849941311 0.8889088661 14.5554420498

H 8.3315207142 2.2987375991 14.6279477853

C 8.78258377 0.9715763817 12.9320324226

H 9.8291646177 1.194222502 13.1670775599

H 8.6869479854 -0.1208011344 12.9365675271

C 3.6375594695 2.6603460132 8.8306231085

C 2.9907840745 2.8118191366 7.6024577435

H 2.7725112976 3.8013579177 7.2077189363

C 2.6571956656 1.678274241 6.8649629439

H 2.1577371477 1.7983772622 5.9078247802

C 2.9774254267 0.4081260979 7.3377437645

H 2.7235938097 -0.4719855722 6.7540081355

C 3.6282397148 0.270966502 8.5619152228

H 3.880697069 -0.7167119376 8.93821701

C 3.9533177338 1.3931053774 9.3184587379

H 4.4470593858 1.2796971807 10.278264747

C 7.212349055 3.0195488681 6.4246850381

H 8.2772894904 2.7809122859 6.2834466178

C 6.713310033 3.8121481842 5.2150012168

H 7.2392938997 4.7595189454 5.0712102701

H 6.8534958432 3.2119821594 4.3084248389

H 5.6474182127 4.0439116165 5.3096723559

C 6.4134580467 1.7272447039 6.5653583669

H 6.7552708452 1.1022886389 7.3937667702

H 5.3520548688 1.9402382227 6.7190372788

H 6.5040359282 1.1491509611 5.6384859259

C 8.7730555018 4.9418492298 8.0749208178

H 9.5342169833 4.1629232902 7.9226700769

C 8.9233353789 6.004540788 6.9892015419

H 8.9161513826 5.5826848294 5.9804872695

H 8.1324488119 6.7607140236 7.0600207229

H 9.8799831074 6.5224627137 7.1228895642

C 8.9511994167 5.5542124283 9.4634864967

H 8.9209311712 4.7980200637 10.253373114

H 9.9259989991 6.0532629763 9.511198421

H 8.1806804215 6.3064687304 9.6671440565

References

1. Armarego, W. L. F.; Chai, C. L. L., *Purification of Laboratory Chemicals (6th Edition)*. 6th ed.; Elsevier: Burlington.
2. Ditto, S. R.; Card, R. J.; Davis, P. D.; Neckers, D. C., Synthesis and photochemistry of 2,4,6-tri-tert-butylacetophenone. *The Journal of Organic Chemistry* **1979,** *44* (5), 894-896.
3. Chalmers, B. A.; Athukorala Arachchige, K. S.; Prentis, J. K. D.; Knight, F. R.; Kilian, P.; Slawin, A. M. Z.; Woollins, J. D., Sterically Encumbered Tin and Phosphorus peri-Substituted Acenaphthenes. *Inorganic Chemistry* **2014,** *53* (16), 8795-8808.
4. Yu, L.; Wang, J.; Chen, T.; Wang, Y.; Xu, Q., Recyclable 1,2-bis[3,5-bis(trifluoromethyl)phenyl]diselane-catalyzed oxidation of cyclohexene with H2O2: a practical access to trans-1,2-cyclohexanediol. *Applied Organometallic Chemistry* **2014,** *28* (8), 652-656.
5. Mont, W.-W. D.; Kubiniok, S.; Lange, L.; Pohl, S.; Saak, W.; Wagner, I. L., Lithium-(2,4,6-tri-tert-butylphenylselenid); Erzeugung, Struktur und Reaktionen unter Knüpfung von Se –P-, Se – C-, Se – Si-, Se – Sn- und Se – Au-Bindungen. *Chemische Berichte* **1991,** *124* (6), 1315-1320.
6. Aschenbach, L. K.; Knight, F. R.; Randall, R. A.; Cordes, D. B.; Baggott, A.; Buhl, M.; Slawin, A. M.; Woollins, J. D., Onset of three-centre, four-electron bonding in peri-substituted acenaphthenes: a structural and computational investigation. *Dalton Trans* **2012,** *41* (11), 3141-53.
7. CrystalClear-SM Expert v2.1 Rigaku Americas, The Woodlands, Texas USA, and Rigaku Corporation, Tokyo, Japan, 2015.
8. *CrysAlisPro*, v1.171.38.41, v1.171.38.43, v1.171.39.8d, v1.171.40.29a, and v1.171.40.40a; Rigaku Oxford Diffraction, Rigaku Corporation: Oxford, U.K., 2015-2019.
9. Sheldrick, G. M., A short history of SHELX. *Acta crystallographica. Section A, Foundations of crystallography* **2008,** *64* (Pt 1), 112-122.
10. Burla, M. C.; Caliandro, R.; Camalli, M.; Carrozzini, B.; Cascarano, G. L.; Giacovazzo, C.; Mallamo, M.; Mazzone, A.; Polidori, G.; Spagna, R., SIR2011: a new package for crystal structure determination and refinement. *Journal of Applied Crystallography* **2012,** *45* (2), 357-361.
11. P. T. Beuerskens, G. Admiraal, G. Beuerskens, W. P. Bosman, R. de-Gelder, R. Israel, J. M. M. Smits, The DIRDIF-99 program system, Technical Report of the Crystallogrpahy Laboratory, University of Nijmegen, The Netherlands. 1999.
12. Palatinus, L.; Chapuis, G., SUPERFLIP– a computer program for the solution of crystal structures by charge flipping in arbitrary dimensions. *J. Appl. Crystallogr.* **2007,** *40* (4), 786-790.
13. Sheldrick, G. M., SHELXT - Integrated space-group and crystal-structure determination. *Acta Crystallographica Section A* **2015,** *71* (1), 3-8.
14. Sheldrick, G. M., Crystal structure refinement with SHELXL. *Acta Crystallogr. Sect. C-Cryst. Struct. Commun.* **2015,** *71* (1), 3-8.
15. Spek, A., PLATON SQUEEZE: a tool for the calculation of the disordered solvent contribution to the calculated structure factors. *Acta Crystallographica Section C* **2015,** *71* (1), 9-18.
16. Spek, A., Structure validation in chemical crystallography. *Acta Crystallographica Section D* **2009,** *65* (2), 148-155.
17. *CrystalStructure*, 4.2 and 4.3.0; Rigaku Americas, The Woodlands, Texas, USA, and Rigaku Corporation, Tokyo, Japan, 2015-2018.
18. Dolomanov, O. V.; Bourhis, L. J.; Gildea, R. J.; Howard, J. A. K.; Puschmann, H., OLEX2: a complete structure solution, refinement and analysis program. *Journal of Applied Crystallography* **2009,** *42* (2), 339-341.
19. Perdew, J. P.; Burke, K.; Ernzerhof, M., Generalized Gradient Approximation Made Simple. *Physical Review Letters* **1996,** *77* (18), 3865-3868.
20. Adamo, C.; Barone, V., Toward reliable density functional methods without adjustable parameters: The PBE0 model. *J. Chem. Phys.* **1999,** *110* (13), 6158-6170.
21. Grimme, S.; Antony, J.; Ehrlich, S.; Krieg, H., A consistent and accurate ab initio parametrization of density functional dispersion correction (DFT-D) for the 94 elements H-Pu. *The Journal of Chemical Physics* **2010,** *132* (15), 154104.
22. Grimme, S.; Ehrlich, S.; Goerigk, L., Effect of the damping function in dispersion corrected density functional theory. *Journal of Computational Chemistry* **2011,** *32* (7), 1456-1465.

23. Risthaus, T.; Grimme, S., Benchmarking of London Dispersion-Accounting Density Functional Theory Methods on Very Large Molecular Complexes. *Journal of Chemical Theory and Computation* **2013,** *9* (3), 1580-1591.
24. Becke, A. D.; Johnson, E. R., Exchange-hole dipole moment and the dispersion interaction. *The Journal of Chemical Physics* **2005,** *122* (15), 154104.
25. Johnson, E. R.; Becke, A. D., A post-Hartree-Fock model of intermolecular interactions: Inclusion of higher-order corrections. *The Journal of Chemical Physics* **2006,** *124* (17), 174104.
26. Andrae, D.; Häußermann, U.; Dolg, M.; Stoll, H.; Preuß, H., Energy-adjustedab initio pseudopotentials for the second and third row transition elements. *Theor. Chim. Acta* **1990,** *77* (2), 123-141.
27. Binning, R. C.; Curtiss, L. A., Compact contracted basis sets for third-row atoms: Ga–Kr. *Journal of Computational Chemistry* **1990,** *11* (10), 1206-1216.
28. Bühl, M.; Reimann, C.; Pantazis, D. A.; Bredow, T.; Neese, F., Geometries of Third-Row Transition-Metal Complexes from Density-Functional Theory. *J. Chem. Theory Comput.* **2008,** *4* (9), 1449-1459.
29. Frisch, M. J.; Trucks, G. W.; Schlegel, H. B.; Scuseria, G. E.; Robb, M. A.; Cheeseman, J. R.; Scalmani, G.; Barone, V.; Mennucci, B.; Petersson, G. A.; Nakatsuji, H.; Caricato, M.; Li, X.; Hratchian, H. P.; Izmaylov, A. F.; Bloino, J.; Zheng, G.; Sonnenberg, J. L.; Hada, M.; Ehara, M.; Toyota, K.; Fukuda, R.; Hasegawa, J.; Ishida, M.; Nakajima, T.; Honda, Y.; Kitao, O.; Nakai, H.; Vreven, T.; Montgomery Jr., J. A.; Peralta, J. E.; Ogliaro, F.; Bearpark, M. J.; Heyd, J.; Brothers, E. N.; Kudin, K. N.; Staroverov, V. N.; Kobayashi, R.; Normand, J.; Raghavachari, K.; Rendell, A. P.; Burant, J. C.; Iyengar, S. S.; Tomasi, J.; Cossi, M.; Rega, N.; Millam, N. J.; Klene, M.; Knox, J. E.; Cross, J. B.; Bakken, V.; Adamo, C.; Jaramillo, J.; Gomperts, R.; Stratmann, R. E.; Yazyev, O.; Austin, A. J.; Cammi, R.; Pomelli, C.; Ochterski, J. W.; Martin, R. L.; Morokuma, K.; Zakrzewski, V. G.; Voth, G. A.; Salvador, P.; Dannenberg, J. J.; Dapprich, S.; Daniels, A. D.; Farkas, Ö.; Foresman, J. B.; Ortiz, J. V.; Cioslowski, J.; Fox, D. J. *Gaussian 09*, Gaussian, Inc.: Wallingford, CT, USA, 2009.
30. M. Repisky, S. Komorovsky, V. G. Malkin, O. L. Malkina, M. Kaupp, K. Ruud, ReSpect, version 5.0.1, 2018; Relativistic Spectroscopy DFT program, 2018. http://www.respectprogram.org
31. Malkin, E.; Repiský, M.; Komorovský, S.; Mach, P.; Malkina, O. L.; Malkin, V. G., Effects of finite size nuclei in relativistic four-component calculations of hyperfine structure. *J. Chem. Phys.* **2011,** *134* (4), 044111.
32. Becke, A. D., Density-functional exchange-energy approximation with correct asymptotic behavior. *Phys. Rev. A* **1988,** *38* (6), 3098-3100.
33. Perdew, J. P.; Yue, W., Accurate and simple density functional for the electronic exchange energy: Generalized gradient approximation. *Phys. Rev. B* **1986,** *33* (12), 8800-8802.
34. Dyall, K. G., Relativistic Double-Zeta, Triple-Zeta, and Quadruple-Zeta Basis Sets for the 4s, 5s, 6s, and 7s Elements. *J. Phys. Chem. A* **2009,** *113* (45), 12638-12644.
35. Dyall, K. G., Relativistic Quadruple-Zeta and Revised Triple-Zeta and Double-Zeta Basis Sets for the 4p, 5p, and 6p Elements. *Theor. Chem. Acc.* **2006,** *115* (5), 441-447.
36. W. Kutzelnigg, U. Fleischer, M. Schindler, in NMR-Basic Principles and Progress, Vol. 23, Eds. P. Diehl, E. Fluck, H. Günther, R. Kosfeld, J. Seelig (Springer Verlag, Heidelberg, 1990), pp. 165.
37. Malkina, O. L.; Malkin, V. G., Visualization of Nuclear Spin–Spin Coupling Pathways by Real-Space Functions. *Angewandte Chemie International Edition* **2003,** *42* (36), 4335-4338.
38. D. R. Salahub, R. Foumier, P. Mlynarski, I. Papai, A. St-Amant, J. Ushio, in: Density Functional Methods in Chemistry. In Density Functional Methods in Chemistry (Eds: J. K. Labanowski, J. W. Andzelm), Springer Verlag, Berlin, 1991; p 77
39. V. G. Malkin, O. L. Malkina, deMon-NMR program, version 2016, Bratislava
40. GaussView Version 4.1.2, Gaussian, Inc., Wallingford 2006.
